# Cytoarchitectural modifications and antiinflammatory strategies in tendinopathy recovery

**DOI:** 10.1371/journal.pone.0335977

**Published:** 2025-11-03

**Authors:** Marta Ramos-Barbero, Eva E. Rufino-Palomares, Sergio Serrano-Carmona, Cristina E. Trenzado, Khalida Mokhtari, José Antonio Lupiáñez, Amalia Pérez-Jiménez

**Affiliations:** 1 Department of Biochemistry and Molecular Biology I, Faculty of Science, University of Granada, Granada, Spain; 2 Sergio Serrano Fisiomedicina Avanzada, Physiotherapy Clinic, Sevilla, Spain; 3 Department of Cell Biology, Faculty of Science, University of Granada, Granada, Spain; 4 Department of Biology, Faculty of Sciences, Laboratory of Bioresources, Biotechnologies, Ethnopharmacology and Health, Mohammed I University of Oujda, Oujda, Morocco; 5 Department of Zoology, Faculty of Science, University of Granada, Granada, Spain; Universiti Malaya, MALAYSIA

## Abstract

Tendinopathies (TPs) are complex conditions marked by inflammation, pain, and impaired function, often due to tendon overuse. Achilles tendinitis, a prevalent TP, affects both athletes and the general population. Despite available treatments, effective tissue regeneration remains elusive. This study investigates the molecular cytoarchitecture and protein expression in TP-related inflammation and evaluates the therapeutic potential of hydroxytyrosol (HT), maslinic acid (MA), glycine/aspartic acid (AA), and their combination with percutaneous intratissue electrolysis (EPI) in a Wistar rat model of induced TP. Animals received a diet supplemented by incorporating the compounds directly into the chow with MA (0.65 g/kg of diet), HT (3 g/kg of diet), and Gly/Asp (Gly: 28.125 g/kg of diet; Asp: 9.375 g/kg of diet). Tendon samples were collected at different TP phases (I, I-II, II, III). Histological analysis (H&E and Masson’s staining) assessed collagen fiber orientation, fibroblast density, and inflammation. Western blotting quantified inflammatory and apoptotic proteins (GST, Hsp60, JNK, NF-κB, PPAR‐γ, p53), while MDA levels indicated oxidative tissue damage. Results demonstrated that combining EPI with nutritional supplementation significantly improved recovery compared to EPI alone. Among the compounds tested, HT showed the most potent effects, followed by MA, reducing inflammation markers and enhancing tendon regeneration. Additionally, MDA levels significantly decreased in the HT group, indicating reduced oxidative stress. In cases where EPI is contraindicated, nutritional supplementation may serve as a viable non-invasive alternative, promoting faster healing and improved long-term outcomes. These findings highlight the potential of integrating EPI and targeted nutritional strategies to optimize TP treatment.

## 1. Introduction

**Fig 1:**
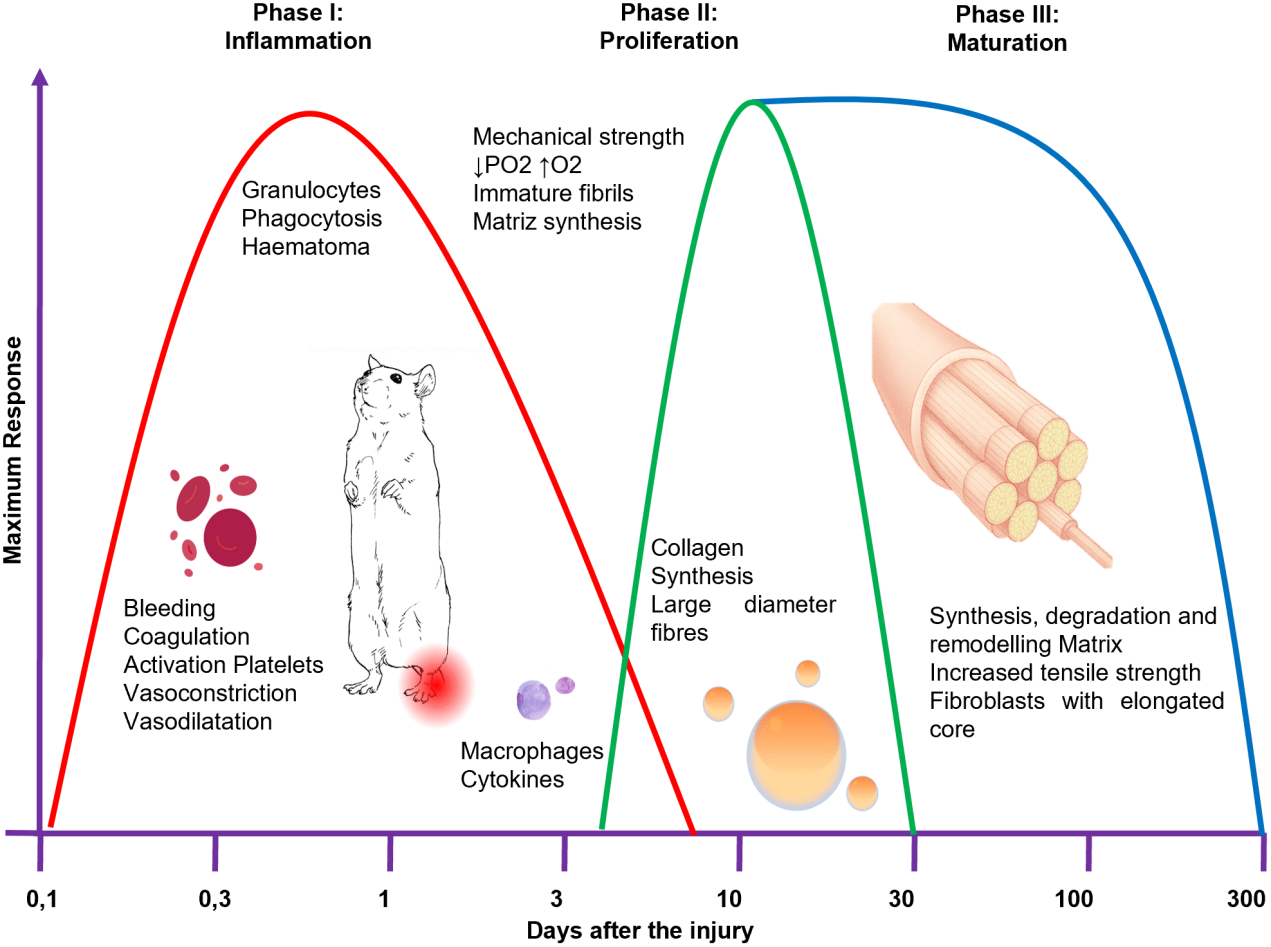
Tendon healing occurs in three consecutive phases: Inflammation (3–7 days), Proliferation (5–21 days), and Remodeling (up to 12 months), involving collagen production, matrix organization, and fiber alignment to restore strength and elasticity (Adapted from [6,7]).

Tendinopathy (TP) is a complex syndrome that can involve inflammation acute or chronic and/or degenerative disorders, although currently there is no consensus. Therefore, TP is considered a multifactorial condition characterized by pain, reduced function, swelling, and tendon deterioration [1,2]. Commonly affected tendons include the rotator cuff, medial and lateral epicondyles of the elbow, patellar tendon, gluteal tendons, and Achilles tendon [3]. Although a general rise is observed, precise incidence rates among subgroups remain unclear [4]. Although the exact incidence among professional and recreational athletes at different anatomical sites remains unclear [5], TP is a widespread condition. TP occurs in three overlapping phases: Inflammation, Proliferation and Maturation. A precise understanding of the phases of tendinopathy is essential for accurate interpretation of the underlying cellular and molecular processes, avoiding analytical biases, enabling proper evaluation of pathogenic mechanisms, and optimizing the design and efficacy of clinical interventions (Fig 1) [6,7]. Inflammatory Phase (3–7 days), characterized by hematoma formation, platelet activation, and macrophage recruitment, which mediate the release of growth factors to initiate extracellular matrix deposition and fibroblast proliferation. Proliferation Phase (days 5–21), during which fibroblasts synthesize collagen, forming immature fibrils that subsequently aggregate into larger fibers to enhance tensile resistance. Remodeling Phase (up to 12 months), defined by increased collagen cross-linking, reorganization of the extracellular matrix, and longitudinal fiber alignment, ultimately optimizing tendon strength and elasticity [6,7].

**Fig 2:**
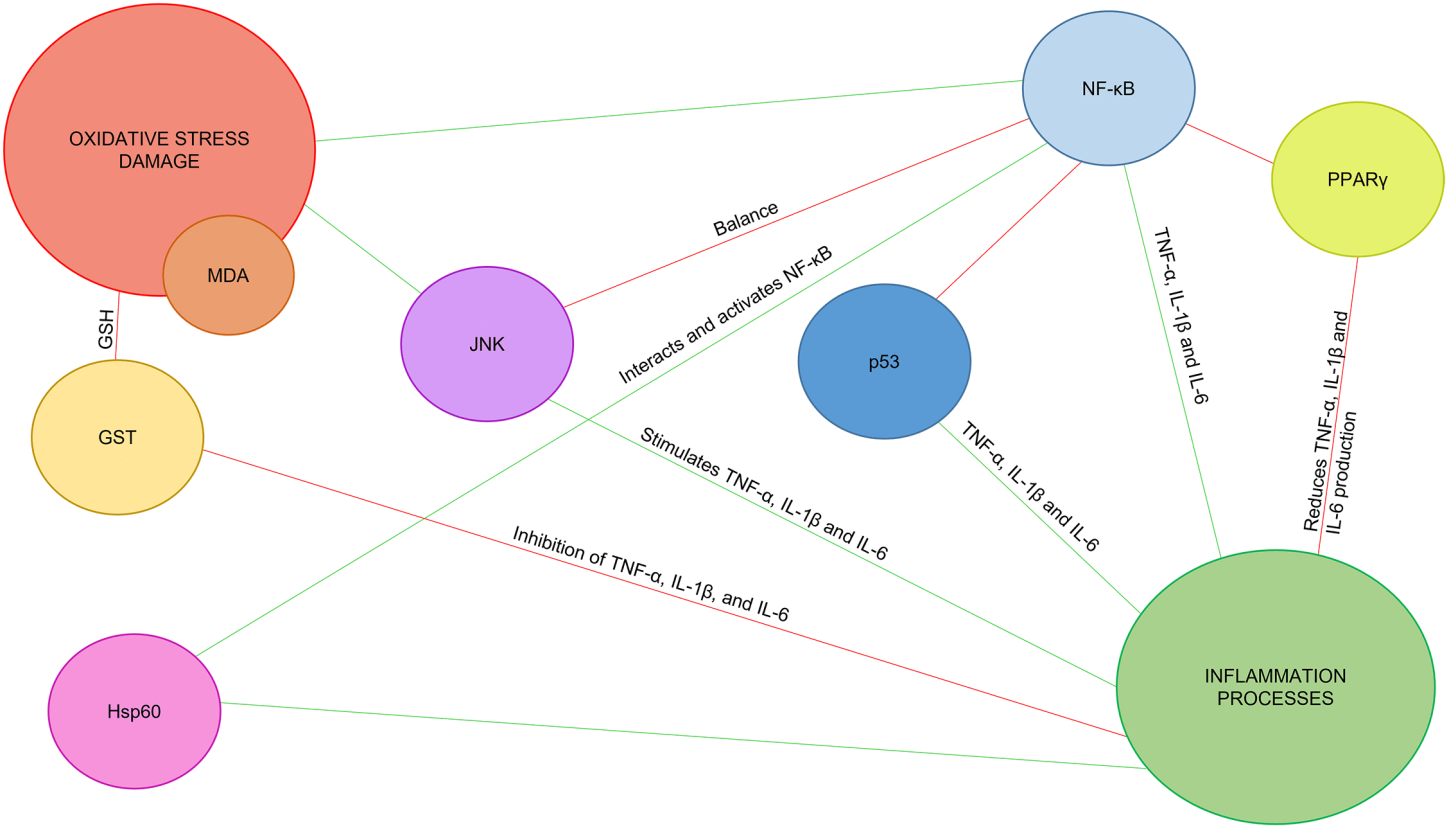
The relationship of the proteins to each other and to the processes of oxidative stress damage and inflammation. Red lines indicate inhibition and green lines indicate promotion or activation. GST: glutathione-S-transferase, Hsp60: Heat shock protein 60, JNK: C Jun N-terminal kinases, NF-κB: Nuclear factor kappa-light-chain-enhancer of activated B cells, PPAR-γ: Peroxisome proliferator-activated receptors, MDA: malondialdehyde, TNF-α: Tumour necrosis factor alpha, IL-1β: Interleukin-1 beta, IL-6: Interleukin-6, GSH: Glutathione and ROS: Reactive oxygen species.

Structurally, tendons are composed primarily of type I collagen and tenocytes. In pathological conditions, tendons experience inflammation, collagen disorganization, and neovascularization, leading to tissue damage and activation of both healing and damaging processes, including the overexpression of disintegrin and metalloproteinase with thrombospondin motifs (MMPs) [8] MMP-1, −2, −8, −9, and −13 contribute to excessive collagen degradation, thereby disrupting tendon structure and function and impairing its regenerative capacity. Specifically, MMP-1, −8, and −13 target fibrillar collagen, which bears mechanical load, potentially promoting tendon tears. In contrast, MMP-2 and −9 degrade network collagens surrounding fibroblasts, influencing cellular morphology and compromising the tendon’s repair mechanisms [9]. In addition to MMPs, the ADAMTS family of proteases is also involved in extracellular matrix remodeling and tendon maintenance. ADAMTS-2 and ADAMTS-14 are key N-propeptidases that process procollagen type I, crucial for collagen fibril formation in tendons. ADAMTS-2 is upregulated in pathological tendons and linked to connective tissue disorders, while ADAMTS-14 has been associated with tendon pathology and osteoarthritis. ADAMTS-5 expression, however, is reduced in Achilles tendinopathy but related to osteoarthritis. Genetic variations in these ADAMTS genes may affect tendon degeneration and disease risk [10]. During the symptomatic phase, the hypovascularity worsens Achilles tendinopathy by promoting the overexpression of endostatin, increasing vascular endothelial growth factor VEGF and MMP production, which weakens the tendon and raises the risk of rupture. Pain in Achilles tendinopathy is linked to high glutamate concentrations, which stimulate sensory nerves and contribute to pain [11,12]. Currently, there is no definitive treatment for TP, with approaches varying based on the type and phase of the disease [13]. Recent treatments for Achilles tendinopathy focus on non-invasive therapies with nutritional factors hydroxytyrosol, maslinic acid, and amino acid, glycine and aspartic acid (HT, MA, AA: Gly + Asp) and minimally invasive techniques like shock waves, electric fields, and intratissue percutaneous electrolysis (EPI) to improve recovery [14]. This study focuses on the expression of markers related to stress, inflammation, and matrix regulation in the Achilles tendon during tendinopathy. Stress markers include glutathione S-transferase (GST) and malondialdehyde (MDA). Inflammatory markers comprise heat shock protein 60 (Hsp60), c-jun N-terminal kinase (JNK), and nuclear factor kappa B (NF-κB). Additionally, markers involved in matrix regulation and apoptosis, such as peroxisome proliferator-activated receptor gamma (PPAR-γ) and tumor protein p53 (p53), are evaluated. Their roles in these processes affect tendon structure and function and understanding them may provide potential targets for therapeutic intervention (Fig 2). GST plays a vital role in detoxification, protecting against oxidative damage by neutralizing reactive oxygen species (ROS) and regulating proinflammatory pathways like NF-κB [15,16]. Hsp60, essential for mitochondrial function, also regulates inflammation and autoimmunity. When released extracellularly, it triggers inflammatory pathways such as activation of toll-like receptor 4 (TLR4), downstream signaling through NF-κB, NOD-, LRR-, and pyrin domain-containing protein 3 (NLRP3) inflammasome, extracellular signal-regulated kinases 1 and 2 (ERK1/2), and JNK pathways, promoting the production of proinflammatory cytokines [17]. JNK, a mitogen-activated kinase family member, regulates inflammation and promotes proinflammatory genes like IL-1, IL-6, and TNF-α [18]. Inhibiting JNK has been associated with increased MMP-2 and MMP-9, influencing tendon inflammation and regeneration by degrading damaged extracellular matrix components (ECM), making JNK inhibition a potential therapeutic strategy for tendon pathologies [19,20]. NF-κB signaling, activated by cytokines and ROS, regulates inflammation and apoptosis, working in conjunction with JNK signaling [21]. PPAR-γ inhibits transcription factors like NF-κB and Activator protein 1, exerting anti-inflammatory effects by reducing the expression of pro-inflammatory genes [22,23]. p53 regulates inflammation and ECM homeostasis, both essential for tendon regeneration. It inhibits MMPs, preventing excessive ECM degradation, and promotes apoptosis in inflammatory cells. This apoptotic process aids tendinopathy resolution by limiting prolonged inflammation and associated tissue damage, thus facilitating tendon repair and restoration of normal structure and function [24]. Additionally, increased tenocyte apoptosis may be an early feature of rotator cuff tendinopathy and could represent a potential therapeutic target [25]. Alongside other oxidative stress-related proteins such as GST, MDA is included as a widely recognized biomarker of lipid peroxidation and an indicator of oxidative damage, providing valuable insights into the underlying biochemical alterations associated with this condition [25].

Standard treatments include pharmacological, physiotherapeutic, and rehabilitation methods [26]. Among these, EPI is a promising treatment that uses ultrasound-guided non-thermal electrochemical ablation to stimulate tendon regeneration by inducing localized inflammation, enhancing oxygen levels, and triggering cellular phagocytosis [6,11]. This treatment increases the expression of proteins like cytochrome C, VEGF, and PPAR-γ while reducing TNF-α and IL-1 levels [27]. A key advantage of EPI is its minimally invasive nature, requiring only a thin needle for current delivery [28]. The combination of EPI with nutraceuticals has been studied for reducing inflammation and promoting tendon regeneration in Wistar rats. HT + EPI and AA + EPI were identified the most effective treatments since reduce the activity of key enzymes related to inflammation and intermediary metabolism as demonstrated by Ramos-Barbero et al. (2024) [29,30]. Given the established roles of inflammation and oxidative stress in tendon degeneration, and the therapeutic potential of EPI combined with nutritional treatments, this study aims to investigate the influence of these nutritional factors (HT, AM and AA), both individually and in combination with EPI, on the cytoarchitecture and molecular response of target proteins involved in the inflammatory process in tendons of Wistar rats with induced tendinopathy.

## 2. Materials and methods

### 2.1. Animals, experimental conditions and sampling

A total of 144 male albino Wistar rats Crl:CD (SD) code (Charles Rives, Laboratories International, Inc., Wilmington, MA, USA), were housed at the Biomedical Instrumentation Center (CIBM), University of Granada, under approved conditions by the Animal Experimentation Ethics Committee of the University of Granada, in accordance with the European Directive 2010/63/EU, with the authorization number 05/07/216/231. Animals were distributed into nine experimental groups: healthy control (C), diseased control (DC), intratissue percutaneous electrolysis (EPI), hydroxytyrosol (HT), maslinic acid (MA), amino acids (AA: glycine + aspartate), EPI combined with hydroxytyrosol (EPI + HT), EPI combined with maslinic acid (EPI + MA), and EPI combined with amino acids (EPI + AA: glycine + aspartate). For each group, 16 animals were assigned, housed in 4 cages with 4 rats per cage. Animals received a diet supplemented by incorporating the nutraceutical compounds directly into the chow. Experimental diets were formulated including nutraceutical factors as described by Ramos-Barbero et al. (2024) [30] and manufactures by ENVIGO RMS Spain S.L. Nutraceutical factors doses were adjusted according to each MA (0.65 g/kg of diet), HT (3 g/kg of diet), Gly (28.125 g/kg of diet), and Asp (9.375 g/kg of diet). Tendinopathy (TP) was induced by injecting 50 µg of collagenase type I into the right Achilles tendon. EPI treatment was administered on day 6 following Ramos-Barbero et al. (2024) protocol [30]. The experiment lasted 41 days.

During the experiment, sampling was performed on key days coinciding with each phase of tendinopathy progression. Sampling points were as follows, considering the day of departure, zero, as the day on which the TP was induced: inflammatory (day 6), transition (day 13), proliferative (day 26), and remodeling (day 40). On day 6, only experimental groups without EPI treatment were sampled (C, DC, HT, MA and AA), whereas the rest of the sampling days all experimental groups were collected. The number of rats sampled was 4 for each treatment at each time point.

Animals were sacrificed with an overdose of sodium pentobarbital (200 mg sodium pentobarbital/kg rat body weight), from the commercial company Eutanax®, which was administered intraperitoneally at a dose that depended on the weight; and the right tendon was removed and immediately frozen in liquid nitrogen and then stored at −80°C until use.

### 2.2. Histological treatment

Tendon tissues were fixed with paraformaldehyde (4%) for 12 hours at 4°C, then dehydrated, cleared, embedded in paraffin, and sectioned into 5 μm thickness using a Leica RM2135 microtome. Four high-power fields per section were randomly selected and the mean value was used. Sections were dried for 24 hours, rehydrated, and stained with haematoxylin and eosin (H&E) and Masson’s stain. Samples were examined using optical microscopy (Leica DM2000) and images captured with a Leica video-camera ICC50 W (software LAS EZ3.4).

### 2.3. Histopathological evaluation

Images obtained from four rats per experimental group, which was randomly selected, were analyzed. The aspects used for qualitative analysis (Table 1) and comparison of samples under the optical microscope were evaluated according to Megías et al. [31] and Fernández-Sarmiento [32].

**Table 1:** Parameter of qualitative analysis.

Parameter	Diseased tissue: Score 0	Partially diseased tissue: Score 1	Healthy tissue: Score 2
**Fibroblast nuclear morphology**	Predominantly oval or rounded nuclei; loss of alignment	Mixed nuclei (oval and elongated), partial alignment	Flattened and elongated nuclei, aligned with collagen
**Fibroblast density**	Low cellularity (<50 fibroblasts/field)	Moderate (50–100 fibroblasts/field)	High cellularity (>100 fibroblasts/field)
**Vascular response**	Absence of visible capillaries or vessels	Sparse or isolated capillaries	Numerous and clearly visible capillaries/vessels
**Tissue inflammation**	No visible inflammatory cells	Scattered macrophages/granulocytes near lesion	Dense clusters of inflammatory cells
**Organization of collagen fibers**	Disorganized, loose, irregularly stained fibers	Partially aligned fibers, some disorganization	Densely packed, parallel, and well-aligned fibers

### 2.4. Tendon protein extraction

Tendons were homogenised in RIPA buffer (150 mM NaCl, 1% Igepal, 0.5% deoxycholic acid, 0.1% sodium dodecyl sulfate (SDS), 50 mM Tris HCl pH 7.5, 0.2 M phenylmethylsulfonyl fluoride, 700 mM sodium orthovanadate (Na_3_VO_4_) and Thermo Scientific® PierceTM inhibitor cocktail, Waltham, MA, USA), and held on ice for 15 min. They were centrifuged at 15,000 × g for 10 min at 4°C, the supernatant was collected and stored at −80°C for further analysis. The quantification of the protein concentration was carried out through the Bradford method [33].

### 2.5. Immunoblot

The expression of GST, Hsp60, JNK, NF-kB, PPAR-γ and p53 proteins was analysed by western blot. The separation of the proteins was performed by a 12% polyacrylamide gel electrophoresis under denaturing conditions (SDS-PAGE) at 100-200V for 1 hour in a Mini-Protean II electrophoresis system (Bio-Rad, Richmond, VA, USA). A standardized load of 30 μg of protein per well was used after denaturing the samples at 95°C for 5 min and adding the loading buffer (0.25 mM Tris-HCl pH 6.8, 10% SDS, 1 M DTT and glycerol). Protein marker IV (VWR, Radnor, PA, USA) was employed as the protein molecular weight marker. The separated proteins were transferred to a Polyvinylidene Fluoride membrane using a semi-dry transfer method (60 mA per gel, 1 hour) using the Transblot Turbo system (BioRad®, Berkeley, CA, USA). Membranes were blocked (TBS, 0.1% Tween-20 and 3% skimmed milk) and were incubated with primary antibodies specific for the target proteins (GST, Hsp60, JNK, NF-kB, PPAR-γ and p53) overnight at 4°C.

The primary antibodies (from Santa Cruz Biotechnology®, Dallas, TX, USA and Sigma-Aldrich, St. Louis, MO, USA) were diluted in blocking buffer TBS, 0.1% Tween-20 and 3% skimmed milk). The membranes were washed with TBS-T (TBS, 0.1% Tween-20) for 5 min three times under stirring, and incubated with the corresponding secondary antibody (from Sigma®, St. Louis, MO, USA for 1 h at room temperature, repeating the previous washing process later). Dilutions and specifications of antibodies are showed in Table 2. The secondary antibodies have the enzyme horseradish peroxidase (HRP) coupled, which allows chemiluminescence to be detected in the presence of the protein band due to its ability to oxidize the luminol solution which containing 1 ml solution A (200 mL 0.1 M Tris-HCl pH 8.6, 50 mg luminol), 100 µL solution B (11 mg p-hydroxy coumaric acid, 10 mL DMSO) and 0.3 µL H_2_O_2_ (30%). Molecular Imager® ChemiDoc™ XRS+ Imaging System from Bio-Rad (Bio-Rad Laboratories, Inc., California, USA) was used to view the lanes and bands. The program used to analyze the images obtained was Image Lab Software (Bio-Rad®, for PC 6.1 version). The quantification of several protein levels by densitometric analysis is shown in bar graphs. The results are expressed as arbitrarian units (A.U.) of expression compared to actin.

**Table 2:** Description of the antibodies (IgG) used in the Western blot analysis.

Protein	Primary Antibody	Secondary Antibody	Catalog number
**GST**	Rabbit anti-GST antibody (1:1000)	Anti-rabbit antibody (1:5000)	G7781 (Sigma Aldrich)
**Hsp60**	Goat anti-Hsp60 (K-19) antibody (1:1000)	Anti-goat antibody (1:5000)	Sc-1722 (Santa Cruz Biotechnology, INC.)
**JNK**	Goat anti-JNK (Thr 183/Tyr 185) antibody (1:500)	Anti-goat antibody (1:5000)	Sc-12882 (Santa Cruz Biotechnology, INC.)
**NF-kB**	Rabbit anti-NF-kB p65 (A) antibody (1:500)	Anti-rabbit antibody (1:5000)	Sc-109 (Santa Cruz Biotechnology, INC.)
**PPAR-γ**	Mouse anti-PPAR-γ (B-5) antibody (1:1.000)	Anti-mouse antibody (1:5000)	Sc-271392 (Santa Cruz Biotechnology, INC.)
**p53**	Rabbit anti-p53 (FL393) antibody (1:500)	Anti-rabbit antibody (1:5000)	Sc-6243 (Santa Cruz Biotechnology, INC.)
**Actin**	Mouse anti-actin (C4) antibody (1:1.000)	Anti-mouse antibody (1:5000)	Sc-47778 (Santa Cruz Biotechnology, INC.)

### 2.6. Lipid peroxidation level

Lipid peroxidation levels in the tendon were determined based on the thiobarbituric acid reacting substance. In presence of thiobarbituric acid, lipoperoxides react producing a coloured substance which was measured as described by the method Buege and Aust (1978) [34] and modified by Pérez-Jiménez et al. (2009) [35]. A standard curve was generated using known concentrations of MDA ranging from 0 to 40 µmol/mg. The results were expressed as µmol MDA per gram of tendon.

### 2.7. Statistical analysis of the results

Cohen’s kappa coefficient (κ) is a statistical measure used to assess the level of agreement between observers or measurement method, comparing the observed agreement (Po) with the expected agreement by chance (Pe). In this study, two independent observers performed the evaluations following a standardized protocol, and both were blinded to the group assignments and treatment conditions to prevent bias. Generally, κ > 0.75 indicates substantial agreement, κ between 0.40 and 0.75 is moderate, and κ < 0.40 is poor [36].

Results are expressed as the mean ± standard error of the mean (SEM). Since the assumptions of normality and homogeneity of data were not met; non-parametric statistical tests were applied. Differences between multiple groups were assessed using the Kruskal-Wallis test, followed by a stepwise step-down comparisons method. A p-value < 0.05 was considered statistically significant. The statistical analyses were performed using SPSS version 25.0 (IBM Corp., Armonk, NY, USA).

## 3. Results

### 3.1. Cytoarchitecture analysis

**Fig 3:**
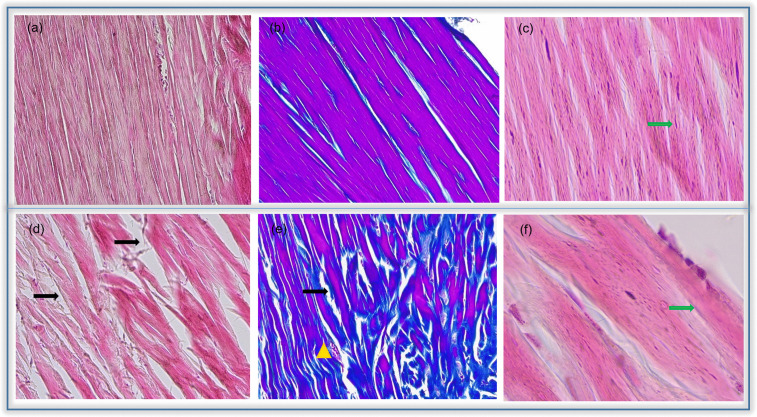
Achilles tendon micrograph. Comparison between Phase III of healthy control group (C) and Phases I and III of diseased control group (DC). Green arrows indicate nucleus of fibroblast. Black arrows indicate separation between fibres of collagen. Yellow triangles indicate vascularised areas by the presence of blood vessels. Sections in paraffin (5 μm), Haematoxylin-eosin (a, c, d, f) and Masson (b, e) staining original magnification x20 (a, b, d, e) and x100 (c, f).

**Fig 4:**
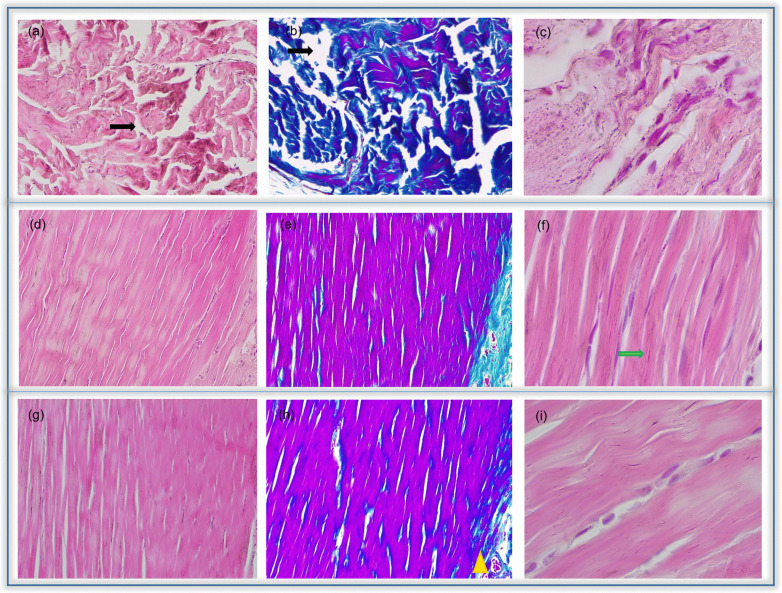
Achilles tendon micrograph: Comparison between Phase I-II EPI. Green arrows indicate the nucleus of fibroblast. Black arrows indicate separation between fibres of collagen. Yellow triangles indicate vascularised areas by the presence of blood vessels. Sections in paraffin (5 μm), haematoxylin-eosin (a, c, d, f, g, i) and Masson (b, e h) staining. Original magnification x20 (a, b, d, e, g, h) and x100 (c, f, i).

Cytoarchitecture results showed the histological progression of each TP phase under different treatments (Fig 3 and 4). The level of agreement between observers was high, with κ = 0.869 for fibroblast nuclear morphology, κ = 0.929 for fibroblast density, κ = 0.906 for vascular response, κ = 0.917 for tissue inflammation, and κ = 0.892 for collagen fiber organization.

Haematoxylin-eosin and Masson staining were performed to compare the healthy control (C) and disease control (DC) (Fig 3). In C group, fibroblasts had elongated nuclei and well-organized collagen fibers (Fig 3a–c). The DC group showed ovoid nuclei, disorganized fibers, inflammation, vascular network expansion, and collagen separation (Fig 3d–f). Fibroblast density was higher in C group across all phases, while DC group showed low fibroblast density in the early phases of TP, followed by a gradual increasing in proliferation as phases TP progressed (Fig 3). Masson’s staining revealed arteriole dilation in DC group and compact, parallel collagen fibers in C group (Fig 3b, e). Structural damage in DC group included higher cellular density near damaged areas (Fig 3d–f).

Fig 4 showed a haematoxylin-eosin and Masson staining between the EPI, HT and EPI + HT groups. In earlier Phases, the EPI group exhibited a higher concentration of adjacent tissue compared to the C group and revealed increased inflammation, with compacted collagen fibers interspersed with large voids (Fig 4a). In Phase II and III, neovascularization was evident and fibroblast density was similar to that of the C group in all phases (Fig 4a, b). Collagen fibers in the EPI group appeared disorganized in Phases I and II (Fig 4a, b) but showed partial reorganization by Phase III. Regarding fibroblast nuclei, the EPI group displayed oval-shaped nuclei in Phases I and II (Fig 4c), which progressively elongated and became more diffusely distributed in Phase III. The EPI + HT and EPI + MA groups showed greater tendon structure and organization. Comparing the DC group with those treated with nutritional factors showed an increase of vascularization and irregular connective tissue in adjacent areas. (Fig 4e). In phase II, an increase in fibroblast density was observed in all groups, with the HT group showing the highest levels, the most elongated nuclei and greater vascularization with an increased density of arterioles (Fig 4d–f). Masson’s staining revealed collagen fibres disorganization across treatments in Phases I and II, showing the HT group the highest organization and compactness, especially in Phase III (Fig 4d).

When comparing individual treatments (HT, MA, AA) with their EPI-combined treatments (EPI + HT, EPI + MA, EPI + AA), the EPI + HT group demonstrated the greatest improvement in tendinopathy-associated histological parameters compared to HT group and EPI group (Fig 4) these being in increased collagen fiber compaction (Fig 4g), enhanced vascularization (Fig 4h) and a greater prevalence of oval-shaped nuclei (Fig 4i).

### 3.2. Protein expression levels

#### 3.2.1. Modulation of GST.

**Fig 5:**
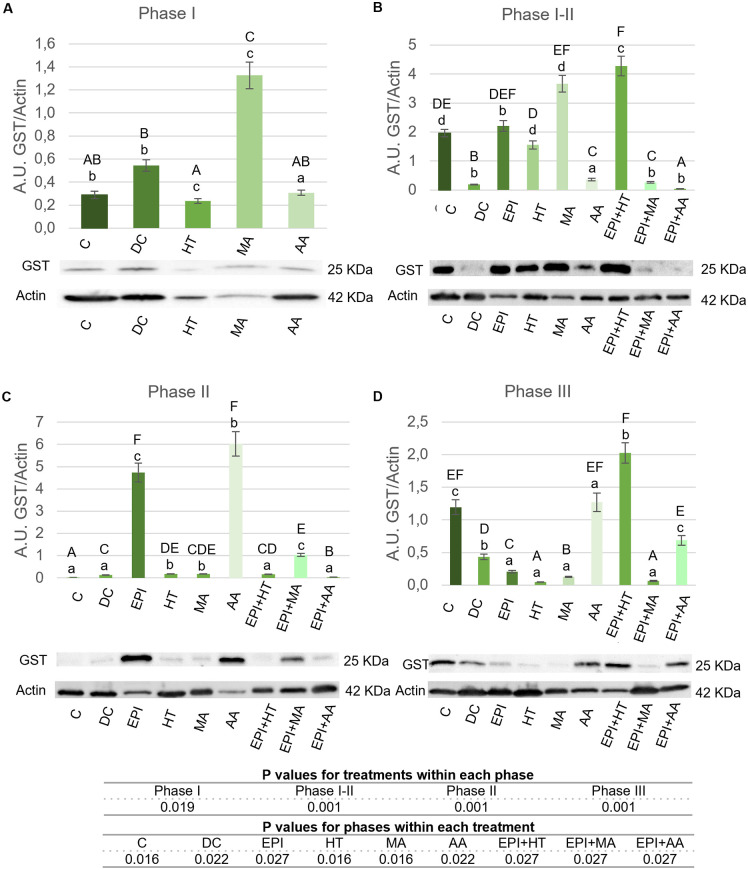
GST expression levels in rat tendon treated with the different nutritional factors, EPI, and combination of both. A: Levels in the first phase of tendinopathy, inflammation phase. B: Expression levels in the first-second phase of tendinopathy, transition phase. C: Expression levels in the second phase, proliferation phase. D: Levels in the third phase of tendinopathy, regeneration phase. C: healthy control, DC: disease control, EPI: intratissue percutaneous electrolysis, HT: hydroxytyrosol, MA: maslinic acid, AA: amino acid. Values are mean ± SEM (n = 3) Capital letters correspond to significant differences (p < 0.05) for effect of treatments within the same phase of tendinopathy. Lower case letters correspond to significant differences (p < 0.05) for the effect of phases within a treatment. P values for statistical analysis are presented in the table under figure.

The GST expression results for different treatments are shown in Fig 5. In Phase I (Fig 5A), GST was significantly increased in the MA group, with no significant changes in the HT and AA groups compared to the C group. In Phase I-II (Fig 5B), GST significantly increased in the EPI + HT group. In phase II, all experimental groups showed a significant increase compared to the C group (Fig 5C). The group that exhibited the greatest increase was the AA treatment In Phase III (Fig 5D), all groups except AA, EPI + HT and EPI + AA exhibited lower GST expression compared to the C group, with EPI + HT showing the highest levels. The AA group had the most significant increase in GST expression during Phase II, followed by the EPI + HT group in Phase I-II and the EPI group in Phase II.

#### 3.2.2. Modulation of Hsp60.

**Fig 6:**
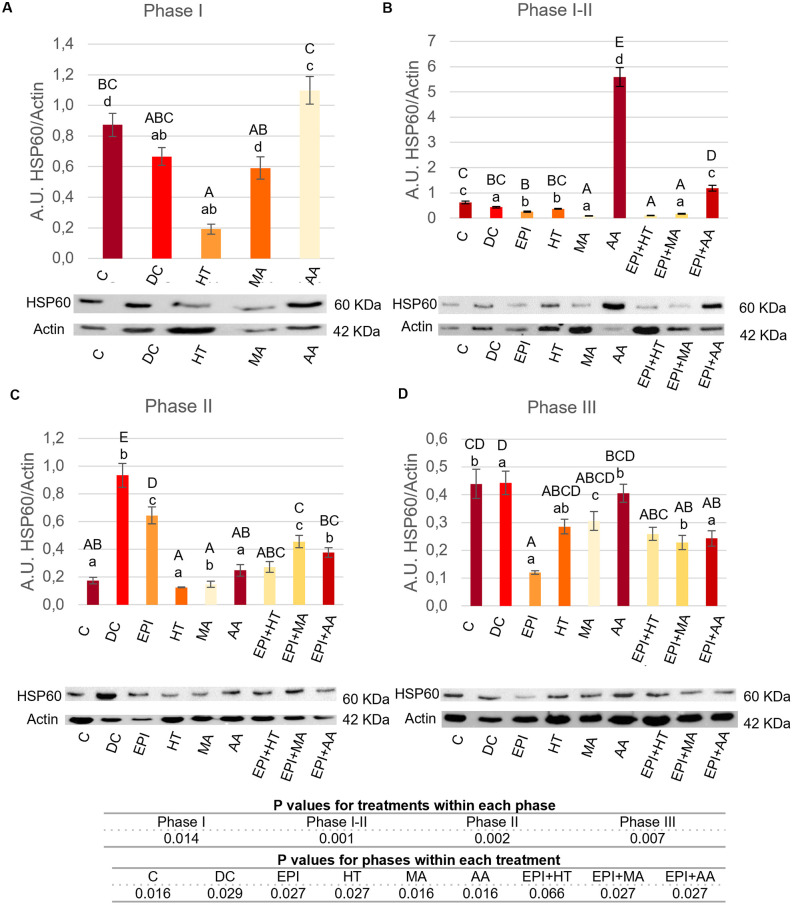
Hsp60 expression levels in rat tendon treated with the different nutritional factors, EPI, and combination of both. A: Levels in the first phase of tendinopathy, inflammation phase. B: Expression levels in the first-second phase of tendinopathy, transition phase. C: Expression levels in the second phase, proliferation phase. D: Levels in the third phase of tendinopathy, regeneration phase. C: healthy control, DC: disease control, EPI: intratissue percutaneous electrolysis, HT: hydroxytyrosol, MA: maslinic acid, AA: amino acid. Values are mean ± SEM (n = 3). Capital letters correspond to significant differences (p < 0.05) for effect of treatments within the same phase of tendinopathy. Lower case letters correspond to significant differences (p < 0.05) for the effect of phases within a treatment. P values for statistical analysis are presented in the table under figure.

In Fig 6 are shown the results for Hsp60 protein expression between different treatments. In Phase I (Fig 6A), the AA group showed the most value of Hsp60 expression, while the HT group exhibited a significant decrease compared to the C groups. The MA group had expression levels similar to the C and DC groups.

In Phase I-II (Fig 6B), the AA group showed the most significant increase in expression, followed by the EPI + AA group. EPI, MA, HT, EPI + MA, EPI + HT treatments showed decreased expression compared to the C groups. Additionally, MA, EPI + HT and EPI + MA groups showed decreased expression with respect to DC treatment.

In Phase II (Fig 6C), Hsp60 expression increased in EPI and EPI + MA groups compared to the C group. All treatments were lower than the DC group.

In Phase III (Fig 6D), EPI, EPI + MA and EPI + AA treatments exhibited a significantly lower expression than the C and DC groups.

#### 3.2.3. Modulation of JNK.

**Fig 7:**
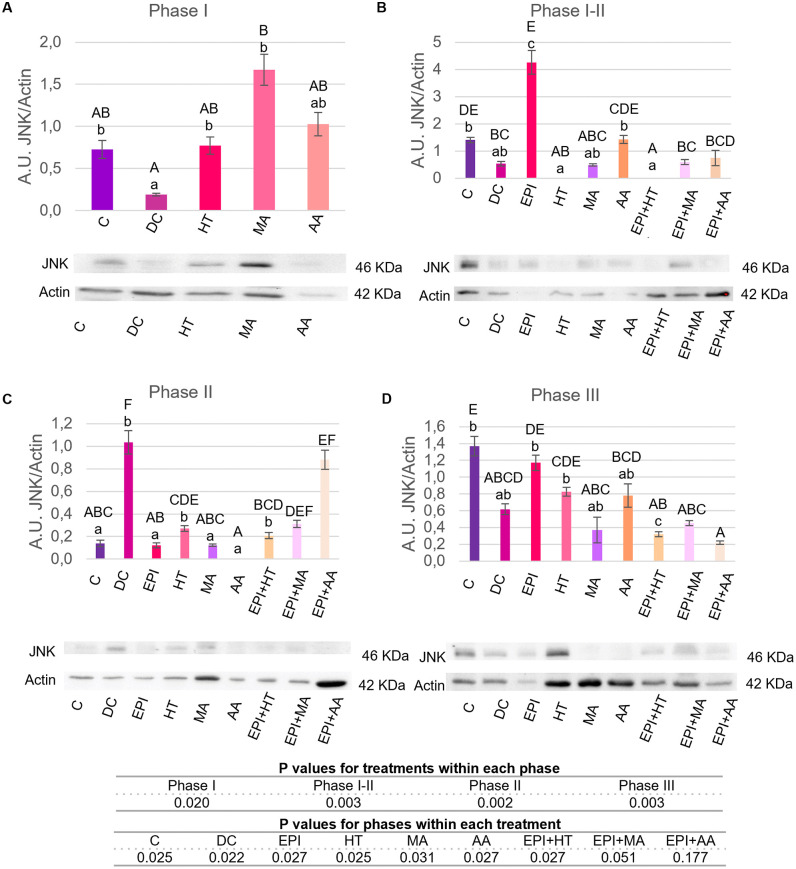
JNK expression levels in rat tendon treated with the different nutritional factors, EPI, and combination of both. A: Levels in the first phase of tendinopathy, inflammation phase. B: Expression levels in the first-second phase of tendinopathy, transition phase. C: Expression levels in the second phase, proliferation phase. D: Levels in the third phase of tendinopathy, regeneration phase. C: healthy control, DC: disease control, EPI: intratissue percutaneous electrolysis, HT: hydroxytyrosol, MA: maslinic acid, AA: amino acid. Values are mean ± SEM (n = 3). Capital letters correspond to significant differences (p < 0.05) for effect of treatments within the same phase of tendinopathy. Lower case letters correspond to significant differences (p < 0.05) for the effect of phases within a treatment. P values for statistical analysis are presented in the table under figure.

The results for JNK protein expression across treatment groups are shown in Fig 7. In Phase I (Fig 7A), MA group exhibited an increase in the JNK expression respect to DC group.

In Phase I-II (Fig 7B), HT, MA, EPI + HT, EPI + MA groups showed a reduction in JNK expression respect to C group, although EPI showed a significant increase compared to C and DC groups.

In Phase II (Fig 7C), the EPI + MA and EPI + AA groups showed an increased JNK expression compared to the C group- The rest of the treatments had lower levels than the DC group, which had the highest expression.

In Phase III (Fig 7D), all treatments exhibited decreased expression compared to the C group, except for the EPI and HT groups. Significant differences were observed in Phase I for the MA group and in Phase I-II for the EPI group.

#### 3.2.4. Modulation of NF-κB.

**Fig 8:**
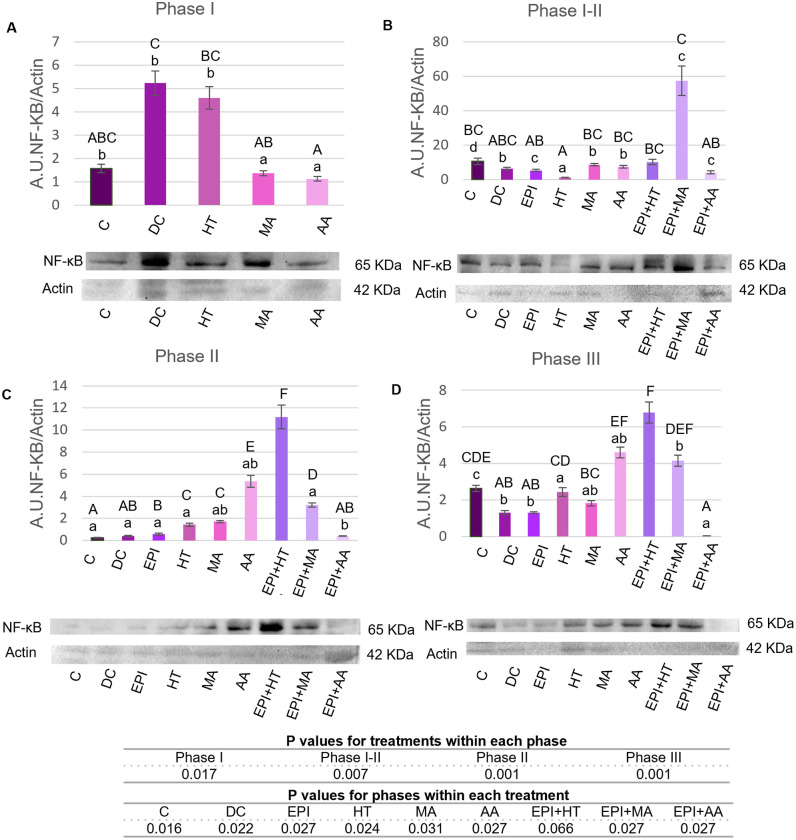
NF-κB expression levels in rat tendon treated with the different nutritional factors, EPI, and combination of both. A: Levels in the first phase of tendinopathy, inflammation phase. B: Expression levels in the first-second phase of tendinopathy, transition phase. C: Expression levels in the second phase, proliferation phase. D: Levels in the third phase of tendinopathy, regeneration phase. C: healthy control, DC: disease control, EPI: intratissue percutaneous electrolysis, HT: hydroxytyrosol, MA: maslinic acid, AA: amino acid. Values are mean ± SEM (n = 3). Capital letters correspond to significant differences (p < 0.05) for effect of treatments within the same phase of tendinopathy. Lower case letters correspond to significant differences (p < 0.05) for the effect of phases within a treatment. P values for statistical analysis are presented in the table under figure.

In Fig 8 are shown the results for NF-κB protein expression between different treatments. In Phase I (Fig 8A), the DC group had the highest NF-κB expression. The MA and AA groups had significantly reduced expression compared to the DC group, similar to the C group.

In Phase I-II (Fig 8B), the HT group showed lower NF-κB expression levels compared to the C group.

In Phase II (Fig 8C), NF-κB expression was upregulated in the HT, MA, AA, EPI + HT, and EPI + MA groups compared to the C and DC groups, with the highest expression in the EPI + HT group.

In Phase III (Fig 8D), NF-κB was decreased in the EPIand EPI + AA groups compared to the C group. The HT, AA, EPI + HT, and EPI + MA groups showed significant increases in NF-κB expression compared to the DC group. NF-κB in the HT group decreased progressively.

#### 3.2.5. Modulation of PPAR-γ.

**Fig 9:**
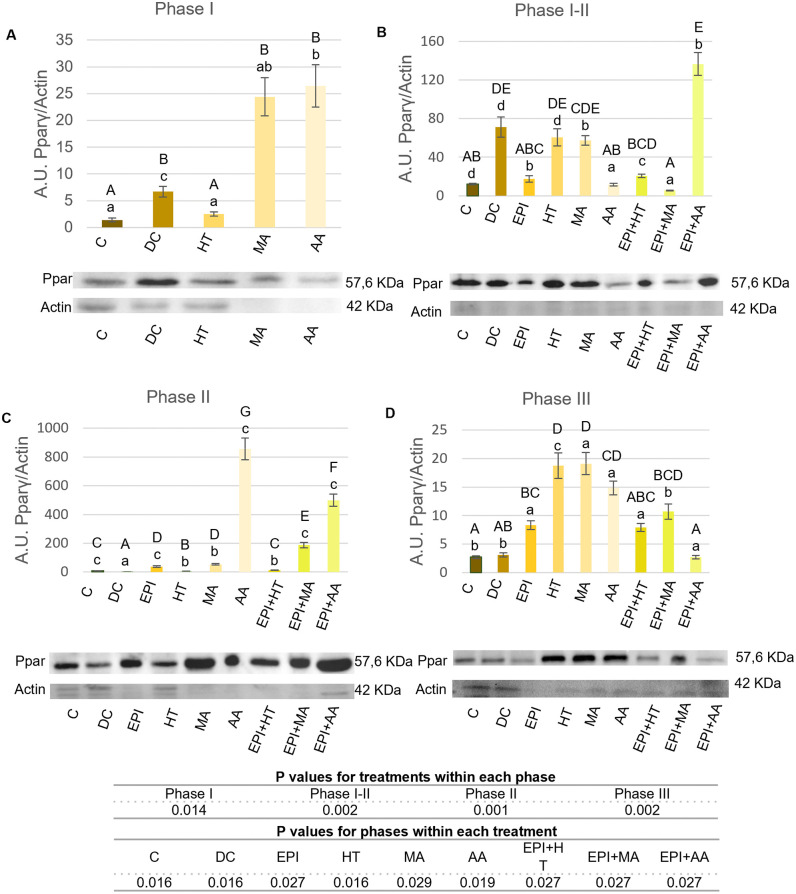
PPAR‐γ expression levels in rat tendon treated with the different nutritional factors, EPI, and combination of both. A: Levels in the first phase of tendinopathy, inflammation phase. B: Expression levels in the first-second phase of tendinopathy, transition phase. C: Expression levels in the second phase, proliferation phase. D: Levels in the third phase of tendinopathy, regeneration phase. C: healthy control, DC: disease control, EPI: intratissue percutaneous electrolysis, HT: hydroxytyrosol, MA: maslinic acid, AA: amino acid. Values are mean ± SEM (n = 3). Capital letters correspond to significant differences (p < 0.05) for effect of treatments within the same phase of tendinopathy. Lower case letters correspond to significant differences (p < 0.05) for the effect of phases within a treatment. P values for statistical analysis are presented in the table under figure.

The PPAR-γ expression results are presented in Fig 9.

In Phase I (Fig 9A), the AA group exhibited the most significant increase in PPAR-γ expression, followed by the MA group. In Phase I-II (Fig 9B), the EPI + AA group showed an increase in the expression compared to the C group. The HT and MA groups showed higher expression than the C group.

In Phase II (Fig 9C), EPI, MA, AA, EPI + MA, and EPI + AA groups showed increased expression compared to the C and DC groups, with the AA group exhibiting the highest increase. In Phase III (Fig 9D), all treated groups, except EPI + HT and EPI + AA, showed increased expression compared to the C group.

Moreover, the HT group showed increased expression in both Phase I-II and Phase III, the MA group in Phase I-II and II, and the AA group in Phase II. The EPI group had higher expression in Phase II, but lower in Phase III (Fig 9).

#### 3.2.6. Modulation of p53.

**Fig 10:**
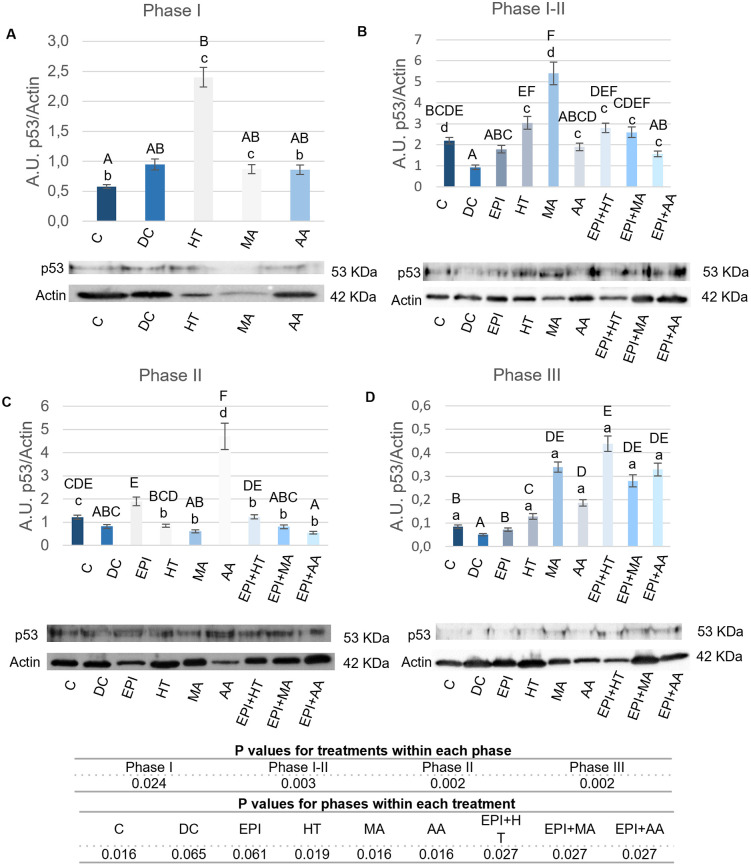
p53 expression levels in rat tendon treated with the different nutritional factors, EPI, and combination of both. A: Levels in the first phase of tendinopathy, inflammation phase. B: Expression levels in the first-second phase of tendinopathy, transition phase. C: Expression levels in the second phase, proliferation phase. D: Levels in the third phase of tendinopathy, regeneration phase. C: healthy control, DC: disease control, EPI: intratissue percutaneous electrolysis, HT: hydroxytyrosol, MA: maslinic acid, AA: amino acid. Values are mean ± SEM (n = 3). Capital letters correspond to significant differences (p < 0.05) for effect of treatments within the same phase of tendinopathy. Lower case letters correspond to significant differences (p < 0.05) for the effect of phases within a treatment. P values for statistical analysis are presented in the table under figure.

The results of p53 protein expression across different phases of TP are shown in Fig 10. In Phase I (Fig 10A), all groups treated with nutritional factors displayed increased expression compared to the C group with the significant values in the HT group.

In the Phase I-II (Fig 10B), the groups treated with HT and MA, both alone and combined with EPI, exhibited higher expression compared to the DC group, with MA showing the greatest differences.

In the Phase II (Fig 10C), only the AA group had higher expression compared to the C group. EPI and EPI + HT groups showed significantly increased values compared to DC group.

In the Phase III (Fig 10D), all treated groups, except EPI, showed higher p53 expression than both the C and DC groups. EPI group only was significantly increased compared to DC group.

Significant differences were observed in p53 expression in Phase I-II for HT, MA, EPI + MA, EPI + HT and EPI + AA, and in Phase II for AA. (Fig 10).

#### 3.2.7. MDA levels.

**Fig 11:**
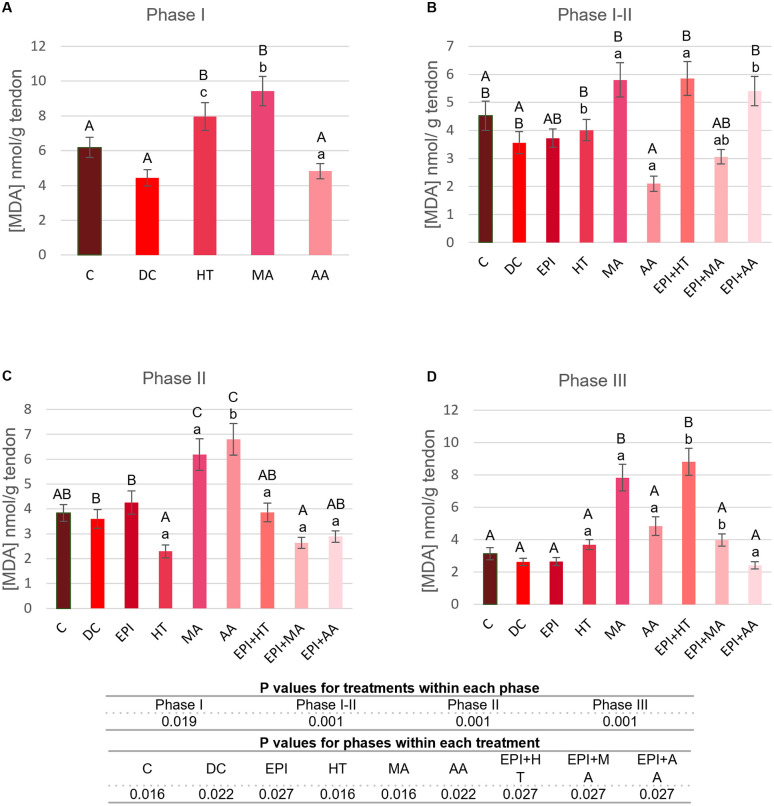
MDA concentration in rat tendon treated with the different nutritional factors, EPI, and combination of both. A: Concentration in the first phase of tendinopathy, inflammation phase. B: Concentration in the first-second phase of tendinopathy, transition phase. C: Concentration in the second phase, proliferation phase. D: Concentration in the third phase of tendinopathy, regeneration phase. C: healthy control, DC: disease control, EPI: intratissue percutaneous electrolysis, HT: hydroxytyrosol, MA: maslinic acid, AA: amino acid. Values are mean ± SEM (n = 3). Capital letters correspond to significant differences (p < 0.05) for effect of treatments within the same phase of tendinopathy. Lower case letters correspond to significant differences (p < 0.05) for the effect of phases within a treatment. P values for statistical analysis are presented in the table under figure.

The changes in MDA concentration across the different phases of TP are shown in Fig 11. In Phase I (Fig 11A), a significant increase in MDA was observed in the HT and MA groups, with MA showing a significant increase compared to the C group.

In Phase I-II (Fig 11B), none of the experimental groups showed significant changes in MDA compared to C and DC groups. no changes were observed in.

In Phase II (Fig 11C), the MA and AA groups showed a significant increase in MDA compared to both the C and DC groups. Conversely, a decrease was noted in the HT and EPI + MA groups with respect to DC group.

In Phase III (Fig 11D), MDA increased in the MA and EPI + HT groups relative to the C and DC groups.

Overall, the MA, HT and EPI + AA groups showed a decrease in MDA concentration in the later phases of TP (Fig 11).

## 4. Discussion

The present study provides novel insights into the pathophysiology and treatment of Achilles tendinopathy by demonstrating marked alterations in tendon cytoarchitecture and differential expression of key inflammatory and oxidative stress markers, such as GST, Hsp60, JNK, NF-κB, PPAR‐γ, p53, and MDA, between healthy and pathological tendons, as well as throughout the progression of the disease. Given that TP is a challenging clinical condition with considerable impact on both work capacity and quality of life [37], there is a pressing need to identify effective therapeutic strategies and characterize the molecular changes that occur during tendon degeneration. In this context, the present work evaluates the anti-inflammatory potential of several treatments in a rat model of induced TP, including intratissue percutaneous electrolysis (EPI), a diet enriched with hydroxytyrosol (HT), maslinic acid (MA), glycine and aspartic acid (AA), and the combined application of these nutritional interventions with EPI. The outcomes support the relevance of these approaches in modulating inflammation, oxidative stress, and tissue integrity during the different stages of TP.

With regard to EPI, an increase in GST, NF-κB and PPAR‐γ were observed in the transition (I-II) and proliferation (II) phases and a decrease of GST and NF-κB in the last phase (III) of the TP. An increase in GST expression matches with the expected biochemical response to pH imbalance and oxidative stress. The ionic instability introduced by EPI technique leads to the formation of sodium hydroxide, which raises the local pH and elevates oxygen pressure [27]. This alteration in the microenvironment stresses the cells, prompting an oxidative response that GST plays a critical role in managing. In this study, GST expression is elevated in response to the oxidative stress induced by the technique. As TP progresses, oxidative stress decreases aligning with expected changes during tissue repair. The least need for GST activity mirrors the decline in inflammation [2]. Thus, the observed changes in GST levels are consistent with the initial oxidative challenge and the subsequent resolution phase in tissue healing. With regard to the analysis of cytoarchitecture by HE and Masson’s trichrome staining’s, it has been found that there is increased inflammation, characterized with the presence of cells such as macrophages and granulocytes, and moreover a higher tissue vascularization [27]. Although the EPI technique produces an increase in anti-inflammatory and angiogenic molecular mechanisms in collagenase-induced tendon injury in rats [27,28], this occurs immediately after applying the technique, subsequently decreasing as the phases of TP progress. However, previous studies have shown a decrease in pro-inflammatory proteins in the intermediary metabolism once TP moves to late phases, indicating a more effective decrease in inflammation in the EPI group throughout the disease [29,30].

Studies have observed that when this galvanic current like EPI is applied directly under the injured tissue, a massive migration of epithelial cells occurs in response to the inflammation. Under normal conditions, NF-κB is inactive due to the action of the specific inhibitor IkappaB kinase (IκB). Inflammatory stimuli have been shown to cause recruitment of the IκB kinase complex, leading to phosphorylation and subsequent degradation of IκB, allowing nuclear translocation of NF-κB enabling the transcription of proinflammatory genes [38,39]. Results found in this study are consistent with initial NF-κB increase and subsequent decrease over the course of TP phases, showing an increase in inflammation and subsequent recovery from injury.

On the other hand, Hsp60 protein only showed an increased expression effect in the inflammation phase of TP, with low levels of this protein when the transition and regeneration phases were studied with respect to the EPI group. These align with the known functions of Hsp60, which is primarily involved in the degradation and clearance of misfolded proteins, chaperone-mediated autophagy, protein quality control mechanisms, cellular signaling, and the formation of the stress response [40]. Furthermore, Hsp60 has been described as a novel molecular link in inflammation, it can be released into the extracellular space and activate pro-inflammatory pathways such as NF-κB, thereby amplifying the initial response. At the intracellular level, it protects tenocytes against oxidative stress and contributes to maintaining protein homeostasis, thus promoting tissue regeneration [41]. This function of Hsp60 is consistent with the findings of this study, where increased in inflammatory phase and progressively decreases in the subsequent phases.

JNK expression, on the other hand, showed an increase in the Phase I-II. These data are in line with the function of this protein, since an excessive increase in this protein can lead to degeneration of the tendon matrix by activation of MMPs [39]. Therefore, the rapid action of JNK in early phases of inflammation will help the process of apoptosis and inflammation, but in later phases, it needs to be reduced to avoid further damage, so that the disease can progress properly towards tissue regeneration. Therefore, fine control of JNK activity could limit tissue damage without impeding the necessary initial inflammatory response, thereby promoting effective tendon repair. Treatments used in this study, combining EPI with nutritional factors, seems to adjust JNK expression in a way that maintains a healthy balance between inflammation and tissue repair [20].

Looking at the HT group, an increase in JNK, p53, NF-κB and PPAR‐γ expression in late phases. However, expression decreases for GST and Hsp60 proteins. HT did not reduce JNK levels as the phases of the disease progress. The JNK effect may be due to the large number of pathways in which this protein participates, MMP-1 activation, extracellular matrix degradation, collagen synthesis, and tendon degeneration [42].

The combined treatment of HT and EPI, showed increases in GST, NF-κB and PPAR‐γ and p53 expression in late phases. These results fit perfectly with the pro-inflammatory role of the EPI technique and the anti-inflammatory role of HT, these anti-inflammatory effects have been demonstrated in various diseases [43–45]. Except for p53, which is involved in apoptosis and is normally present during the course of the disease, GST and Hsp60 are present at higher levels in the transition and proliferation phases, probably due to the role of percutaneous electrolysis in the treatment, increasing localised inflammation and, therefore, inflammatory proteins. These results may indicate that the percentage of EPI participation in the combined treatment is higher than that of HT. This coincides with previous studies assessing liver metabolite alteration in rats with induced TP and concluding that both single treatment with EPI and combined treatment with HT reduces pro-inflammatory enzyme activity [30]. Previous studies have associated increased NF-κB expression and nuclear accumulation in rotator cuff tissue samples in early-stage tendinopathies [46]. Treatments with EPI and HT showed commendable fibre compaction, even larger than the previous group, indicating early tendon recovery due to the anti-inflammatory effects of HT after acute inflammation of the EPI technique. The anti-inflammatory benefits of this phenol have been demonstrated in the reduction of arterial inflammation and microcalcification in the elderly [47].

Further analysis comparing HT alone versus HT combined with EPI reveals that the combined treatment yields enhanced fiber density indices and mitigates inflammation from the Phase II, though inflammation intensifies during the Phase I. This aligns with the roles of platelets in growth factor and cytokine release, where a robust inflammatory response is crucial for subsequent tendon repair. EPI aims to accelerate inflammation to reverse injury, while HT serves as an anti-inflammatory component [27,48].

In the MA group, an increase in GST, JNK, NF-κB, PPAR‐γ and p53 expression is seen in the early phases of TP. Notably, NF-κB, PPAR‐γ and p53 expression levels are also elevated in the late remodelling or maturation phase with respect to this protein. Again, the combination of a nutritional factor with the EPI technique is associated with increased levels of inflammatory protein expression in the inflammatory phase. The role of EPI is precisely to increase the recruitment of pro-inflammatory proteins for an earlier tendon recovery. However, p53 is a pro-apoptotic protein, so its levels increase at the onset of injury. Other authors have seen similar results in tendon injuries, as well as the fact that apoptosis is accompanied by increased cell proliferation in the tendon, suggesting that cell death may be related to ongoing injury repair [49]. PPARs inhibit the activity of transcription factors such as NF-κB, key to the expression of pro-inflammatory genes, decreasing the production of cytokines and inflammatory mediators, and therefore their high presence indicated that the treatment is not reducing inflammation, with these proteins having to act. Its activation promotes the polarization of macrophages toward a reparative phenotype. At the tissue level, it supports tenocyte survival and functionality, promoting collagen synthesis and regeneration. Moreover, it regulates the expression of matrix metalloproteinases and their inhibitors, contributing to an orderly remodeling of the extracellular matrix that restores tendon architecture and function, as it regulates heat shock and oxidative stress proteins, participates in DNA repair, and controls chaperones and proteasomes, among other regulatory subunits, to maintain protein homeostasis [50]. Histological evaluation displayed in this group an increased fibroblast density and collagen fibres compaction in a short period. However, they showed inflammation at later phases, indicating inadequate anti-inflammatory function. The group treated with EPI together with MA showed a compaction of collagen fibres after injury and a more pronounced inflammatory response that is subsequently reduced due to the association of the inflammatory effect of EPI and the anti-inflammatory effect of MA separately. As shown in a recent study, ingested MA not only reduces fatigue and muscle soreness in athletes, but also reduces inflammation and oxidative stress in skeletal muscle [51].

In contrast, the HT group showed a more structured collagen fiber arrangement, reduced inflammation, and an increase in fibroblast density, suggesting enhanced tissue repair. These findings align with the Western blot results, supporting the hypothesis that HT promotes tendon regeneration. Its antioxidant and anti-inflammatory properties may contribute to modulating extracellular matrix remodeling and reducing oxidative damage, as previously reported in studies on skeletal muscle recovery and inflammation control.

By other hand, AA group had elevated GST, NF-κB, PPAR‐γ and p53 expression levels in late phases, and increased Hsp60 expression from Phase I through the transitional phase to Phase II. This indicated that amino acids do not exert an anti-inflammatory function; as inflammatory proteins are still present. Glycine has been shown to modulate the inhibition of TNF-α, which in turn regulates cell healing, proliferation and differentiation, and Interleukin IL-1-β, which mediates healing, growth and differentiation [52]. Aspartic acid is involved in matrix synthesis and degradation. It has been investigated how a small amount of this amino acid in its D-form indicates rapid protein turnover [53]. Some authors have previously seen that JNK and p53 protein increases when an induced tendon injury occurs, causing long-term degenerative changes [44]. Previous studies measuring inflammatory enzymes in the liver metabolism of rats with induced TP when treated with AA revealed no significant alterations in protein or lipid metabolism, but a decrease in carbohydrate metabolism [29]. This treatment only produced slight improvements in fibroblast density and fibre compaction compared to the EPI alone group, although less than the two previous treatments. Thus, both combination and individual therapies using nutritional factors offer promising prognoses against TP.

Finally, the group treated with the combination of AA and EPI presented low levels of GST, Hsp60, JNK expression in late phases of TP. These low levels are interpreted as indicative of inflammation resolution, since these proteins are involved in cellular stress response and inflammatory processes. In contrast, PPAR‐γ and p53 levels were elevated in all phases, suggesting that despite the reduction in inflammatory markers, tissue remodeling and regulated apoptosis processes, essential for tendon repair, are still ongoing. Similar data have been found in previous studies where EPI treatment combined with AA revealed anti-inflammatory effects of carbohydrate metabolism enzymes only in the late phase of TP, but this effect was similar to that revealed by EPI treatment alone [30]. This group showed improvements compared to the EPI group, though to a lesser extent than the previous two groups. Notably, they experienced acute inflammation that persisted until the end of the remodeling phase. Recent systematic reviews and meta-analyses suggest that EPI is effective in reducing pain associated with tendinopathies, highlighting its potential as a minimally invasive treatment [54–56]. These results have also been found in reviews of skeletal muscle and pain reduction when patients were treated with EPI [57].

Increased p53 expression may be attributed to a higher number of apoptotic cells in the tendon. Studies have shown elevated apoptosis in patellar cells of patients with patellar TP compared to C and DC groups, which could explain the rise in p53 levels at the later stages of the disease. This aligns with the known roles of p53 in tissue homeostasis: by promoting apoptosis of damaged or dysfunctional tenocytes, p53 modulates the inflammatory response by limiting the release of pro-inflammatory signals reducing the transcription of cytokine-encoding genes and repressing the activity of other enzymes such as NF-κB. Furthermore, this selective removal of compromised cells facilitates tissue regeneration through the survival and proliferation of healthy tenocytes, as it halts the cell cycle of damaged tenocytes, ensuring that only functional cells proliferate and thus contribute to tendon regeneration. Additionally, p53 regulates ECM remodeling by controlling the turnover and function of tenocytes responsible for collagen synthesis, thus promoting orderly collagen reorganization and restoring tendon structure and function [58,59].

Therefore, this study demonstrated, together with previous studies [29,30], the effectiveness of non-invasive treatments, such as the oral administration of HT, MA and AA groups, both alone and in combination with EPI, as a possible replacement for current ineffective treatments, which have been evaluated as insufficient and that they entail a long rehabilitation period [60].

An elevated MDA is, therefore, a marker of significant cellular damage due to oxidative stress. Thus, the high level of MDA in the HT group and its subsequent decrease indicate a process of damage reversal. However, this did not occur in the group treated with MA, which maintains MDA levels throughout the entire process [26]. Elevated levels of MDA as a result of lipid peroxidation can promote an inflammatory response and thus the activation of all proteins involved in this process, either directly such as GST, or indirectly such as Hsp60, JNK, p53, PPAR-γ y NF-κB.

According to our cytoarchitecture research, employing EPI alone for Achilles tendon TP treatment yields suboptimal outcomes, whereas its adjunctive use with HT, MA, and AA forms an effective therapeutic combination. For patients for whom EPI are medically inadvisable, isolated nutritional treatments may represent a viable option. Presently, there exists no standardized protocol for individuals unsuitable for surgery, relying instead on non-surgical treatments like NSAIDs, which may be counterproductive due to adverse local effects and limited efficacy [61,62]. The administration of HT and MA offers a promising alternative therapy, aiming to supplant ineffective non-invasive treatments and invasive surgical procedures such as percutaneous tenotomies, open debridement, and tendon stripping, which entail prolonged rehabilitation periods and fail to fully restore tendon strength [30].

NF-κB, Hsp60, and JNK are more associated with the activation of inflammation, while PPAR-γ and GST are linked to down-regulating and resolving inflammation. The balance between these factors is crucial for maintaining homeostasis and preventing chronic inflammation from leading to tissue damage. Additionally, several in vitro studies highlight the significant role of oxidative stress in TP pathogenesis, with oxidative damage contributing to the overproduction of ROS and mitochondrial dysfunction in tendon cells, which are key factors in the development of TP [63,64].

## 5. Conclusion

This study has enhanced the understanding of Achilles tendinopathy treatment by identifying key differences in tendon morphology and protein expression between healthy and pathological tendons, as well as across different phases of tendinopathy. It appears that the EPI technique increases early oxidative stress and inflammation, thereby initiating healing; however, these responses must diminish to allow tissue regeneration. Combining EPI with nutritional factors such as HT and MA suggests that it accelerates recovery and enhances tissue regeneration. HT showed the strongest effect, seemingly reducing oxidative markers over time, whereas MA exerted limited effects on oxidative stress and inflammation. There is evidence that combined treatments with EPI, HT, MA, and AA induce an initial inflammatory response but subsequently promote repair by balancing oxidative stress and reducing inflammation in later stages, which may represent a promising therapeutic approach for achieving more effective treatments and shorter recovery times.

## Supporting information

S1 File S1_raw_images_FINAL. File containing the original Western blot images supporting the results presented in the manuscript.
(PDF)

S2 File Supporting information_Zenodo. Dataset containing the raw image analysis data from Western blot experiments and measurements of lipid oxidative damage, deposited in Zenodo.
(ZIP)

## References

[1] Millar NL, Silbernagel KG, Thorborg K, Kirwan PD, Galatz LM, Abrams GD, et al. Tendinopathy. Nat Rev Dis Primers. 2021;7(1):1. doi: 10.1038/s41572-020-00234-1 33414454

[2] Lui PPY, Zhang X, Yao S, Sun H, Huang C. Roles of Oxidative Stress in Acute Tendon Injury and Degenerative Tendinopathy-A Target for Intervention. Int J Mol Sci. 2022;23(7):3571. doi: 10.3390/ijms23073571 35408931 PMC8998577

[3] Hoffmann A, Gross G. Tendon and ligament engineering in the adult organism: mesenchymal stem cells and gene-therapeutic approaches. Int Orthop. 2007;31(6):791–7. doi: 10.1007/s00264-007-0395-9 17634943 PMC2266662

[4] Park S-H, Lee HS, Young KW, Seo SG. Treatment of Acute Achilles Tendon Rupture. Clin Orthop Surg. 2020;12(1):1–8. doi: 10.4055/cios.2020.12.1.1 32117532 PMC7031433

[5] Magra M, Maffulli N. Nonsteroidal antiinflammatory drugs in tendinopathy: friend or foe. Clin J Sport Med. 2006;16(1):1–3. doi: 10.1097/01.jsm.0000194764.27819.5d 16377967

[6] Abate M, Silbernagel KG, Siljeholm C, Di Iorio A, De Amicis D, Salini V, et al. Pathogenesis of tendinopathies: inflammation or degeneration?. Arthritis Res Ther. 2009;11(3):235. doi: 10.1186/ar2723 19591655 PMC2714139

[7] Hope M, Saxby TS. Tendon healing. Foot Ankle Clin. 2007;12(4):553–67, v. doi: 10.1016/j.fcl.2007.07.003 17996614

[8] Riley GP, Curry V, DeGroot J, van El B, Verzijl N, Hazleman BL, et al. Matrix metalloproteinase activities and their relationship with collagen remodelling in tendon pathology. Matrix Biol. 2002;21(2):185–95. doi: 10.1016/s0945-053x(01)00196-2 11852234

[9] Davis ME, Gumucio JP, Sugg KB, Bedi A, Mendias CL. MMP inhibition as a potential method to augment the healing of skeletal muscle and tendon extracellular matrix. J Appl Physiol (1985). 2013;115(6):884–91. doi: 10.1152/japplphysiol.00137.2013 23640595 PMC3764625

[10] Taye N, Karoulias SZ, Hubmacher D. The “other” 15-40%: The Role of Non-Collagenous Extracellular Matrix Proteins and Minor Collagens in Tendon. J Orthop Res. 2020;38(1):23–35. doi: 10.1002/jor.24440 31410892 PMC6917864

[11] Alfredson H, Thorsen K, Lorentzon R. In situ microdialysis in tendon tissue: high levels of glutamate, but not prostaglandin E2 in chronic Achilles tendon pain. Knee Surg Sports Traumatol Arthrosc. 1999;7(6):378–81. doi: 10.1007/s001670050184 10639657

[12] Alfredson H, Ljung BO, Thorsen K, Lorentzon R. In vivo investigation of ECRB tendons with microdialysis technique--no signs of inflammation but high amounts of glutamate in tennis elbow. Acta Orthop Scand. 2000;71(5):475–9. doi: 10.1080/000164700317381162 11186404

[13] Augustyn D, Paez A. The effectiveness of intratissue percutaneous electrolysis for the treatment of tendinopathy: a systematic review. S Afr J Sports Med. 2022;34(1):v34i1a12754. doi: 10.17159/2078-516X/2022/v34i1a12754 36815929 PMC9924571

[14] Loiacono C, Palermi S, Massa B, Belviso I, Romano V, Gregorio AD, et al. Tendinopathy: Pathophysiology, Therapeutic Options, and Role of Nutraceutics. A Narrative Literature Review. Medicina (Kaunas). 2019;55(8):447. doi: 10.3390/medicina55080447 31394838 PMC6723894

[15] Qin J, Wang C, Zhou X. Glutathione regulates CIA-activated splenic-lymphocytes via NF-κB/MMP-9 and MAPK/PCNA pathways manipulating immune response. Cell Immunol. 2024;405–406:104866. doi: 10.1016/j.cellimm.2024.104866 39250860

[16] Lv N, Huang C, Huang H, Dong Z, Chen X, Lu C, et al. Overexpression of Glutathione S-Transferases in Human Diseases: Drug Targets and Therapeutic Implications. Antioxidants (Basel). 2023;12(11):1970. doi: 10.3390/antiox12111970 38001822 PMC10668987

[17] Singh MK, Shin Y, Han S, Ha J, Tiwari PK, Kim SS, et al. Molecular Chaperonin HSP60: Current Understanding and Future Prospects. Int J Mol Sci. 2024;25(10):5483. doi: 10.3390/ijms25105483 38791521 PMC11121636

[18] Sánchez-Vera I, Saura-Esteller J, Núñez-Vázquez S, Cosialls AM, Ghashghaei O, Lavilla R, et al. The prohibitin-binding compound fluorizoline induces the pro-inflammatory cytokines interleukin-8 and interleukin-6 through the activation of JNK and p38 MAP kinases. Biochem Pharmacol. 2023;218:115860. doi: 10.1016/j.bcp.2023.115860 37884196

[19] Li Y, You L, Nepovimova E, Adam V, Heger Z, Jomova K, et al. c-Jun N-terminal kinase signaling in aging. Front Aging Neurosci. 2024;16:1453710. doi: 10.3389/fnagi.2024.1453710 39267721 PMC11390425

[20] Jiang K, Li Y, Xiang C, Xiong Y, Jia J. TGF-β3 regulates adhesion formation through the JNK/c-Jun pathway during flexor tendon healing. BMC Musculoskelet Disord. 2021;22(1):843. doi: 10.1186/s12891-021-04691-x 34592976 PMC8485513

[21] Taniguchi K, Karin M. NF-κB, inflammation, immunity and cancer: coming of age. Nat Rev Immunol. 2018;18(5):309–24. doi: 10.1038/nri.2017.142 29379212

[22] Huang S, Jin Y, Zhang L, Zhou Y, Chen N, Wang W. PPAR gamma and PGC-1alpha activators protect against diabetic nephropathy by suppressing the inflammation and NF-kappaB activation. Nephrology (Carlton). 2024;29(12):858–72. doi: 10.1111/nep.14381 39229715 PMC11579552

[23] Borsting E, Cheng VP-C, Glass CK, Vallon V, Cunard R. Peroxisome proliferator-activated receptor-γ agonists repress epithelial sodium channel expression in the kidney. Am J Physiol Renal Physiol. 2012;302(5):F540-51. doi: 10.1152/ajprenal.00306.2011 22169011 PMC3353644

[24] Zhou M, Liu B, Ye H-M, Hou J-N, Huang Y-C, Zhang P, et al. ROS-induced imbalance of the miR-34a-5p/SIRT1/p53 axis triggers chronic chondrocyte injury and inflammation. Heliyon. 2024;10(11):e31654. doi: 10.1016/j.heliyon.2024.e31654 PMC11140697 38828289

[25] Lundgreen K, Lian Ø, Scott A, Engebretsen L. Increased levels of apoptosis and p53 in partial-thickness supraspinatus tendon tears. Knee Surg Sports Traumatol Arthrosc. 2013;21(7):1636–41. doi: 10.1007/s00167-012-2226-9 23052118

[26] Xiang L, Deng H, Zhou S. Effects of TNF-α on Behaviour and Inflammation in Rats with Rotator Cuff Injury through NGF. Discov Med. 2024;36(185):1241–9. doi: 10.24976/Discov.Med.202436185.114 38926110

[27] Abat F, Valles SL, Gelber PE, Polidori F, Stitik TP, García-Herreros S, et al. Molecular repair mechanisms using the Intratissue Percutaneous Electrolysis technique in patellar tendonitis. Rev Esp Cir Ortop Traumatol. 2014;58(4):201–5. doi: 10.1016/j.recot.2014.01.002 24821478

[28] Mattiussi G, Moreno C. Treatment of proximal hamstring tendinopathy-related sciatic nerve entrapment: presentation of an ultrasound-guided “Intratissue Percutaneous Electrolysis” application. Muscles Ligaments Tendons J. 2016;6(2):248–52. doi: 10.11138/mltj/2016.6.2.248 27900300 PMC5115258

[29] Ramos-Barbero M, Rufino-Palomares EE, Serrano-Carmona S, Hernández-Yera M, García-Salguero L, Lupiáñez JA, et al. Effect of Nutraceutical Factors on Hepatic Intermediary Metabolism in Wistar Rats with Induced Tendinopathy. Int J Mol Sci. 2024;25(1):629. doi: 10.3390/ijms25010629 38203800 PMC10779845

[30] Ramos-Barbero M, Pérez-Jiménez A, Serrano-Carmona S, Mokhtari K, Lupiáñez JA, Rufino-Palomares EE. The Efficacy of Intratissue Percutaneous Electrolysis (EPI®) and Nutritional Factors for the Treatment of Induced Tendinopathy in Wistar Rats: Hepatic Intermediary Metabolism Effects. Int J Mol Sci. 2024;25(13):7315. doi: 10.3390/ijms25137315 39000426 PMC11242821

[31] Megías M, Molist P, Pombal MA. Atlas of plant and animal histology. Histological techniques. 2019. http://mmegias.webs.uvigo.es/6-tecnicas/1-introduccion.php

[32] Fernández Sarmiento J. Evaluación de la reparación tendinosa tras la aplicación de plasma rico en factores de crecimiento en un modelo experimental de rotura de tendón de aquiles en oveja. Serv Publicaciones la Univ Córdoba. 2012;1(1):1–475.

[33] Bradford MM. A rapid and sensitive method for the quantitation of microgram quantities of protein utilizing the principle of protein-dye binding. Analytical Biochemistry. 1976;72(1–2):248–54. doi: 10.1016/0003-2697(76)90527-3 942051

[34] Buege JA, Aust SD. Microsomal lipid peroxidation. Methods Enzymol. 1978;52:302–10. doi: 10.1016/s0076-6879(78)52032-6 672633

[35] Pérez-Jiménez A, Hidalgo MC, Morales AE, Arizcun M, Abellán E, Cardenete G. Use of different combinations of macronutrients in diets for dentex (Dentex dentex): effects on intermediary metabolism. Comp Biochem Physiol A Mol Integr Physiol. 2009;152(3):314–21. doi: 10.1016/j.cbpa.2008.11.002 19049895

[36] Cohen J. A coefficient of agreement for nominal scales. Educ Psychol Meas. 1960;20:37–46.

[37] Albers IS, Zwerver J, Diercks RL, Dekker JH, Van den Akker-Scheek I. Incidence and prevalence of lower extremity tendinopathy in a Dutch general practice population: a cross sectional study. BMC Musculoskelet Disord. 2016;17:16. doi: 10.1186/s12891-016-0885-2 26759254 PMC4711046

[38] Marzagalli M, Battaglia S, Raimondi M, Fontana F, Cozzi M, Ranieri FR, et al. Anti-Inflammatory and Antioxidant Properties of a New Mixture of Vitamin C, Collagen Peptides, Resveratrol, and Astaxanthin in Tenocytes: Molecular Basis for Future Applications in Tendinopathies. Mediators Inflamm. 2024;2024:5273198. doi: 10.1155/2024/5273198 39108992 PMC11303056

[39] Kumar A, Takada Y, Boriek AM, Aggarwal BB. Nuclear factor-kappaB: its role in health and disease. J Mol Med (Berl). 2004;82(7):434–48. doi: 10.1007/s00109-004-0555-y 15175863

[40] Habich C, Sell H. Heat shock proteins in obesity: links to cardiovascular disease. Horm Mol Biol Clin Investig. 2015;21(2):117–24. 10.1515/hmbci-2014-0040 25781556

[41] Chebotareva N, Bobkova I, Shilov E. Heat shock proteins and kidney disease: perspectives of HSP therapy. Cell Stress Chaperones. 2017;22(3):319–43. doi: 10.1007/s12192-017-0790-0 28409327 PMC5425374

[42] Wang F, Murrell GAC, Wang M-X. Oxidative stress-induced c-Jun N-terminal kinase (JNK) activation in tendon cells upregulates MMP1 mRNA and protein expression. J Orthop Res. 2007;25(3):378–89. doi: 10.1002/jor.20294 17106880

[43] Chen C, Ai Q, Wei Y. Hydroxytyrosol protects against cisplatin-induced nephrotoxicity via attenuating CKLF1 mediated inflammation, and inhibiting oxidative stress and apoptosis. Int Immunopharmacol. 2021;96:107805. doi: 10.1016/j.intimp.2021.107805 34162164

[44] Mokhtari K, Pérez-Jiménez A, García-Salguero L, A Lupiáñez J, Rufino-Palomares EE. Unveiling the Differential Antioxidant Activity of Maslinic Acid in Murine Melanoma Cells and in Rat Embryonic Healthy Cells Following Treatment with Hydrogen Peroxide. Molecules. 2020;25(17):4020. doi: 10.3390/molecules25174020 32899159 PMC7504795

[45] Zhang X, Cao J, Zhong L. Hydroxytyrosol inhibits pro-inflammatory cytokines, iNOS, and COX-2 expression in human monocytic cells. Naunyn Schmiedebergs Arch Pharmacol. 2009;379(6):581–6. doi: 10.1007/s00210-009-0399-7 19198806

[46] Abraham AC, Shah SA, Golman M, Song L, Li X, Kurtaliaj I, et al. Targeting the NF-κB signaling pathway in chronic tendon disease. Sci Transl Med. 2019;11(481):eaav4319. doi: 10.1126/scitranslmed.aav4319 30814338 PMC6534967

[47] Zoubdane N, Abdo R-A, Nguyen M, Bentourkia M, Turcotte EE, Berrougui H, et al. High Tyrosol and Hydroxytyrosol Intake Reduces Arterial Inflammation and Atherosclerotic Lesion Microcalcification in Healthy Older Populations. Antioxidants (Basel). 2024;13(1):130. doi: 10.3390/antiox13010130 38275655 PMC10812987

[48] Robles-Almazan M, Pulido-Moran M, Moreno-Fernandez J, Ramirez-Tortosa C, Rodriguez-Garcia C, Quiles JL, et al. Hydroxytyrosol: Bioavailability, toxicity, and clinical applications. Food Res Int. 2018;105:654–67. doi: 10.1016/j.foodres.2017.11.053 29433260

[49] Lundgreen K, Lian Ø, Scott A, Engebretsen L. Increased levels of apoptosis and p53 in partial-thickness supraspinatus tendon tears. Knee Surg Sports Traumatol Arthrosc. 2013;21(7):1636–41. doi: 10.1007/s00167-012-2226-9 23052118

[50] Fu Q, Shen N, Fang T, Zhang H, Di Y, Liu X, et al. ACT001 alleviates inflammation and pyroptosis through the PPAR-γ/NF-κB signaling pathway in LPS-induced alveolar macrophages. Genes Genomics. 2024;46(3):323–32. doi: 10.1007/s13258-023-01455-w 37831404

[51] Shirai T, Myoenzono K, Kawai E, Yamauchi Y, Suzuki K, Maeda S, et al. Effects of maslinic acid supplementation on exercise-induced inflammation and oxidative stress in water polo athletes: A randomized, double-blind, crossover, and placebo-controlled trial. J Int Soc Sports Nutr. 2023;20(1):2239196. doi: 10.1080/15502783.2023.2239196 37498159 PMC10375926

[52] Vieira CP, De Oliveira LP, Da Ré Guerra F, Marcondes MCC, Pimentel ER. Green Tea and Glycine Modulate the Activity of Metalloproteinases and Collagen in the Tendinitis of the Myotendinous Junction of the Achilles Tendon. Anat Rec (Hoboken). 2016;299(7):918–28. doi: 10.1002/ar.23361 27121758

[53] Thorpe CT, Streeter I, Pinchbeck GL, Goodship AE, Clegg PD, Birch HL. Aspartic acid racemization and collagen degradation markers reveal an accumulation of damage in tendon collagen that is enhanced with aging. J Biol Chem. 2010;285(21):15674–81. doi: 10.1074/jbc.M109.077503 20308077 PMC2871433

[54] Gómez-Chiguano GF, Navarro-Santana MJ, Cleland JA, Arias-Buría JL, Fernández-de-Las-Peñas C, Ortega-Santiago R, et al. Effectiveness of Ultrasound-Guided Percutaneous Electrolysis for Musculoskeletal Pain: A Systematic Review and Meta-Analysis. Pain Med. 2021;22(5):1055–71. doi: 10.1093/pm/pnaa342 33155055

[55] Asensio-Olea L, Leirós-Rodríguez R, Marqués-Sánchez MP, de Carvalho FO, Maciel LYS. Efficacy of percutaneous electrolysis for the treatment of tendinopathies: A systematic review and meta-analysis. Clin Rehabil. 2023;37(6):747–59. doi: 10.1177/02692155221144272 36583575

[56] Ferreira MHL, Araujo GAS, De-La-Cruz-Torres B. Effectiveness of Percutaneous Needle Electrolysis to Reduce Pain in Tendinopathies: A Systematic Review With Meta-Analysis. J Sport Rehabil. 2024;33(5):307–16. doi: 10.1123/jsr.2024-0009 38897578

[57] Sánchez-González JL, Navarro-López V, Cañada-Sánchez P, Juárez-Vela R, de Viñaspre-Hernández RR, Varela-Rodríguez S. Efficacy of different intensities of percutaneous electrolysis for musculoskeletal pain: A systematic review and meta-analysis. Front Med (Lausanne). 2023;10:1101447. doi: 10.3389/fmed.2023.1101447 36817790 PMC9932994

[58] Lian Ø, Scott A, Engebretsen L, Bahr R, Duronio V, Khan K. Excessive apoptosis in patellar tendinopathy in athletes. Am J Sports Med. 2007;35(4):605–11. doi: 10.1177/0363546506295702 17244903

[59] Carrà G, Lingua MF, Maffeo B, Taulli R, Morotti A. P53 vs NF-κB: the role of nuclear factor-kappa B in the regulation of p53 activity and vice versa. Cell Mol Life Sci. 2020;77(22):4449–58. doi: 10.1007/s00018-020-03524-9 32322927 PMC11104960

[60] Alfredson H, Pietilä T, Lorentzon R. Chronic Achilles tendinitis and calf muscle strength. Am J Sports Med. 1996;24(6):829–33. doi: 10.1177/036354659602400620 8947407

[61] Fredberg U. Local corticosteroid injection in sport: review of literature and guidelines for treatment. Scand J Med Sci Sports. 1997;7(3):131–9. doi: 10.1111/j.1600-0838.1997.tb00129.x 9200316

[62] Yusuf S, Reddy S, Ounpuu S, Anand S. Global burden of cardiovascular diseases: part I: general considerations, the epidemiologic transition, risk factors, and impact of urbanization. Circulation. 2001;104(22):2746–53. doi: 10.1161/hc4601.099487 11723030

[63] Zhang X, Eliasberg CD, Rodeo SA. Mitochondrial dysfunction and potential mitochondrial protectant treatments in tendinopathy. Ann N Y Acad Sci. 2021;1490(1):29–41. doi: 10.1111/nyas.14599 33843069

[64] Zhang X, Wada S, Zhang Y, Chen D, Deng X-H, Rodeo SA. Assessment of Mitochondrial Dysfunction in a Murine Model of Supraspinatus Tendinopathy. J Bone Joint Surg Am. 2021;103(2):174–83. doi: 10.2106/JBJS.20.00385 32941310

