# Supplementary material for: Cytoarchitectural modifications and antiinflammatory strategies in tendinopathy recovery
Source: PLoS One. 2025 Nov 3;20(11):e0335977. doi: 10.1371/journal.pone.0335977 (PMC12582488; doi:10.1371/journal.pone.0335977)
Supplement: S1 File — File containing the original Western blot images supporting the results presented in the manuscript. (PDF) [file pone.0335977.s001.pdf]

Treatment nomenclature:

C: healthy control

DC: diseased control

EPI: intratissue percutaneous electrolysis

HT: hydroxytyrosol

MA: maslinic acid

AA: amino acids (glycine + aspartate)

EPI+HT: EPI combined with hydroxytyrosol

EPI+MA: EPI combined with maslinic acid

EPI+AA: EPI combined with amino acids (glycine + aspartate)

GST-Phase A (Figure 5)

Biorad Image lab, (Software 6.1 Windows)

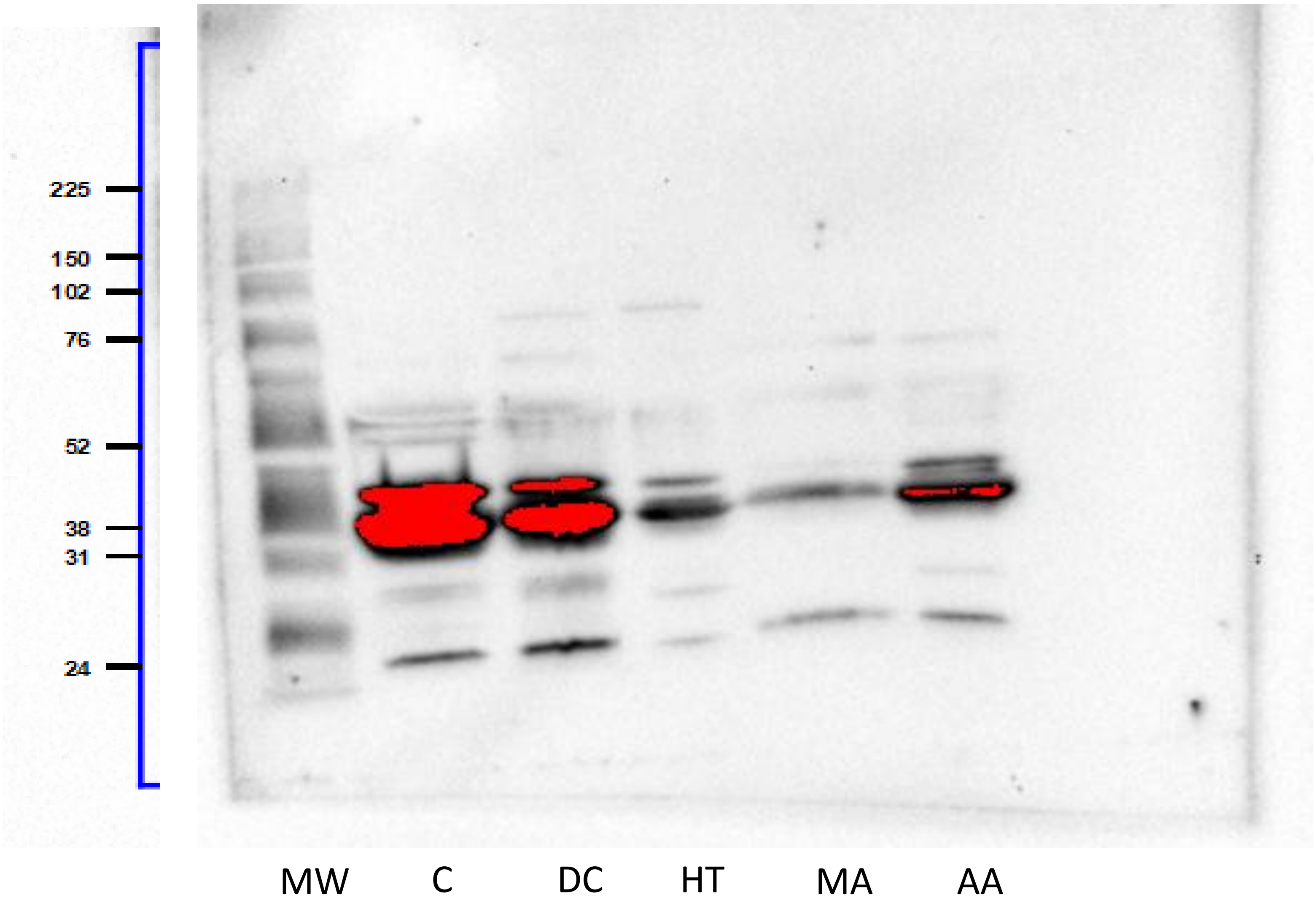

ACTIN (GST)-Phase A

Biorad Image lab, (Software 6.1 Windows)

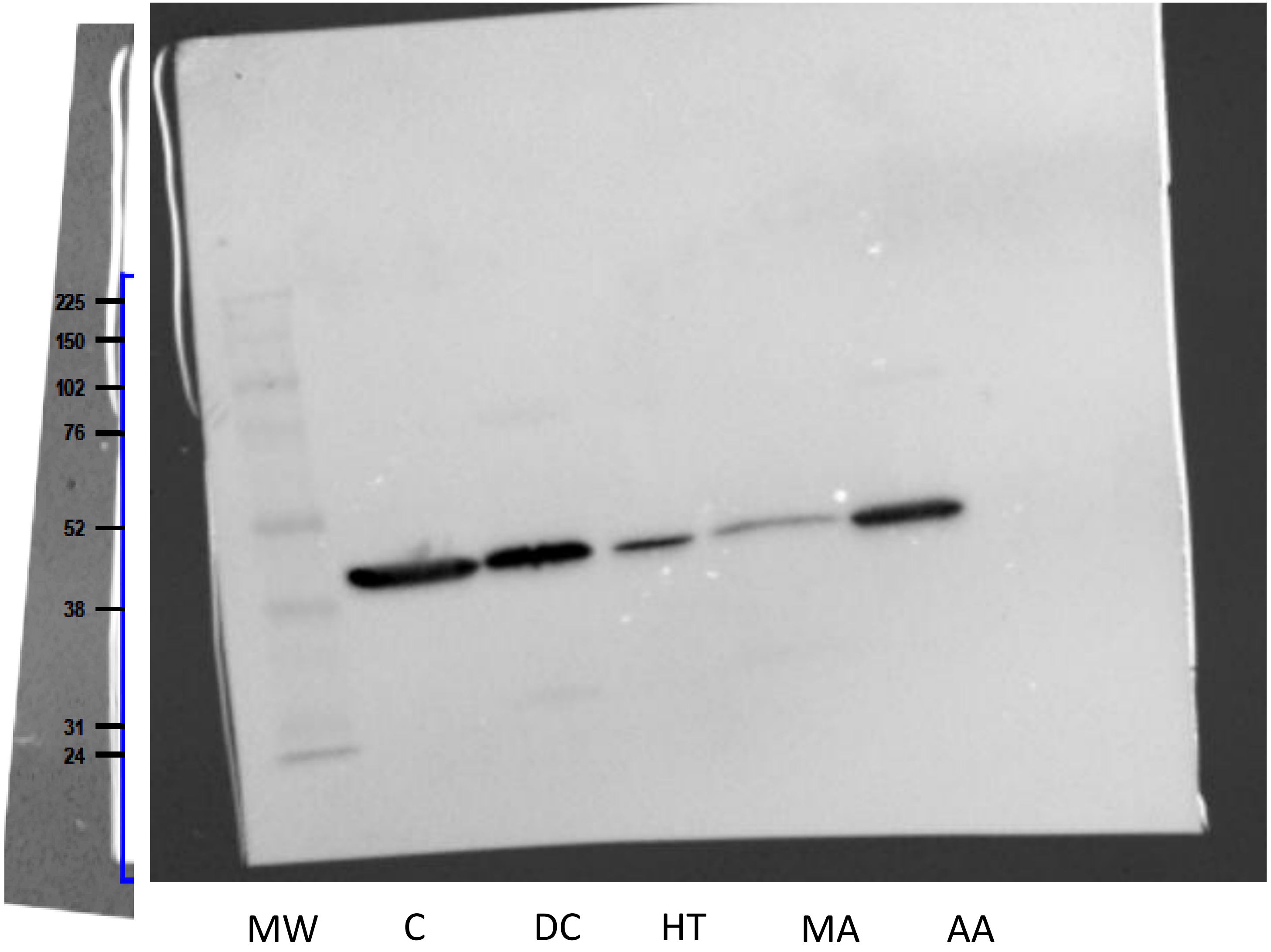

GST-Phase B

Biorad Image lab, (Software 6.1 Windows)

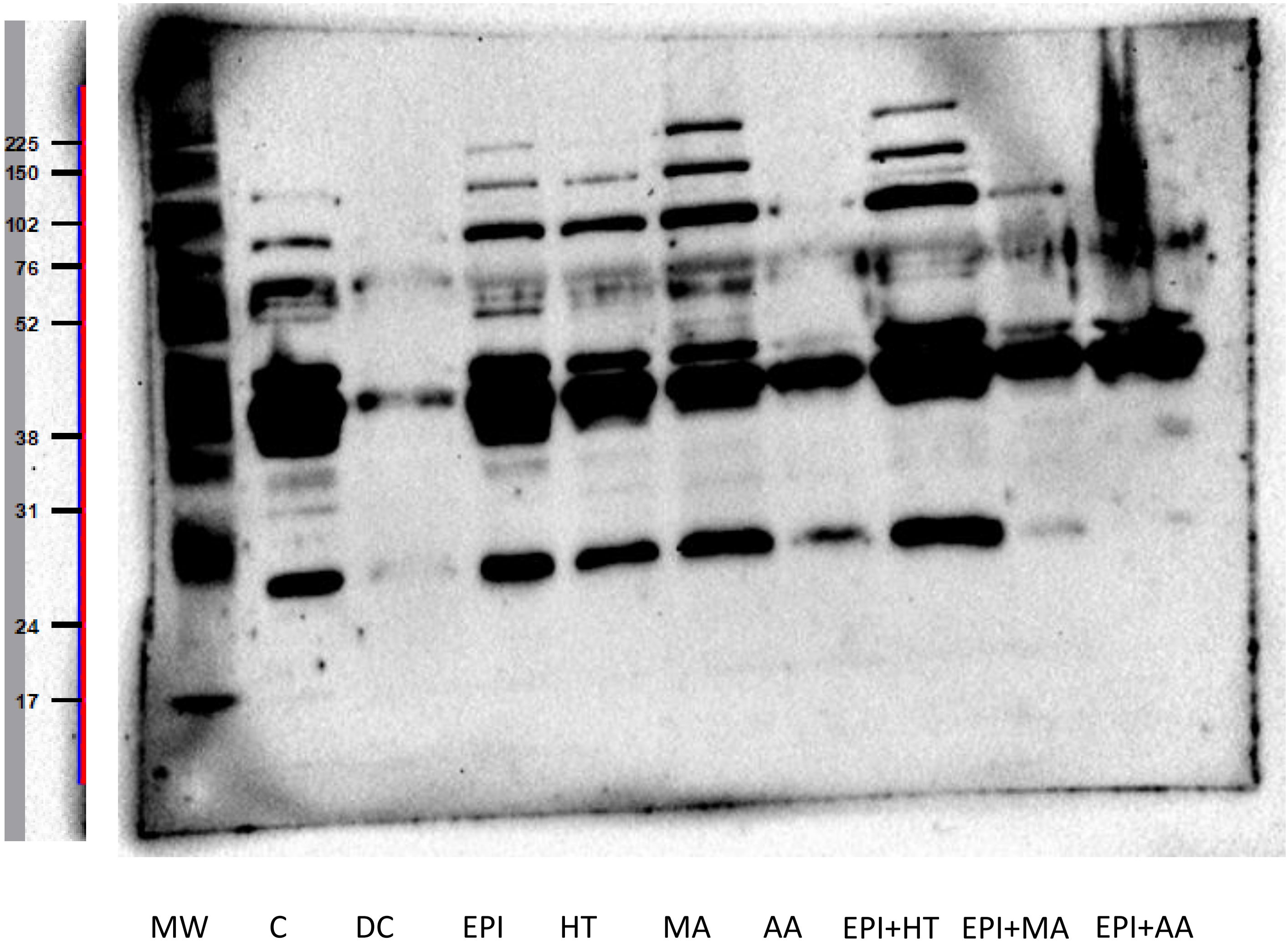

ACTIN (GST)-Phase B

Biorad Image lab, (Software 6.1 Windows)

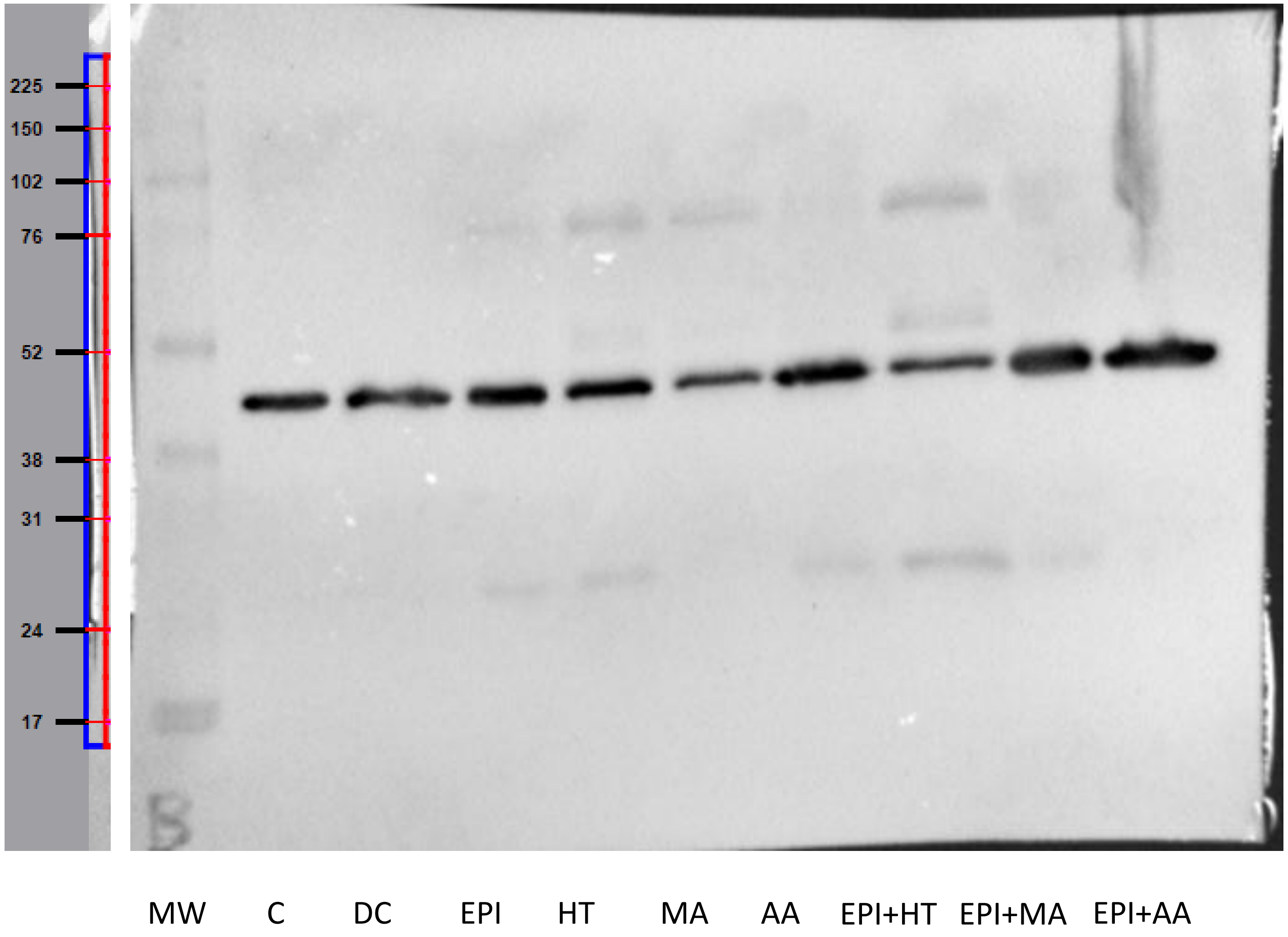

GST-Phase C

Biorad Image lab, (Software 6.1 Windows)

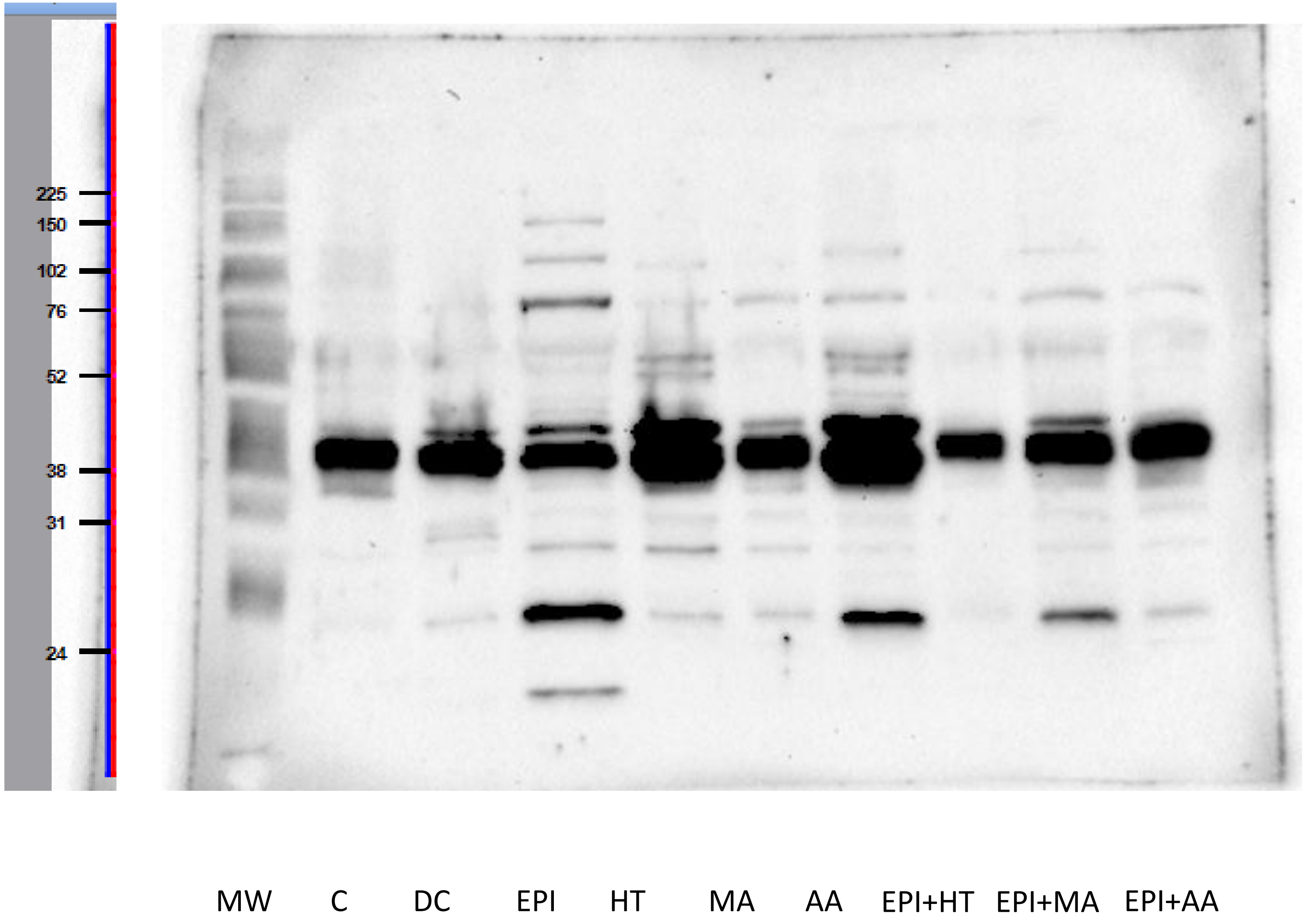

ACTIN (GST)-Phase C

Biorad Image lab, (Software 6.1 Windows)

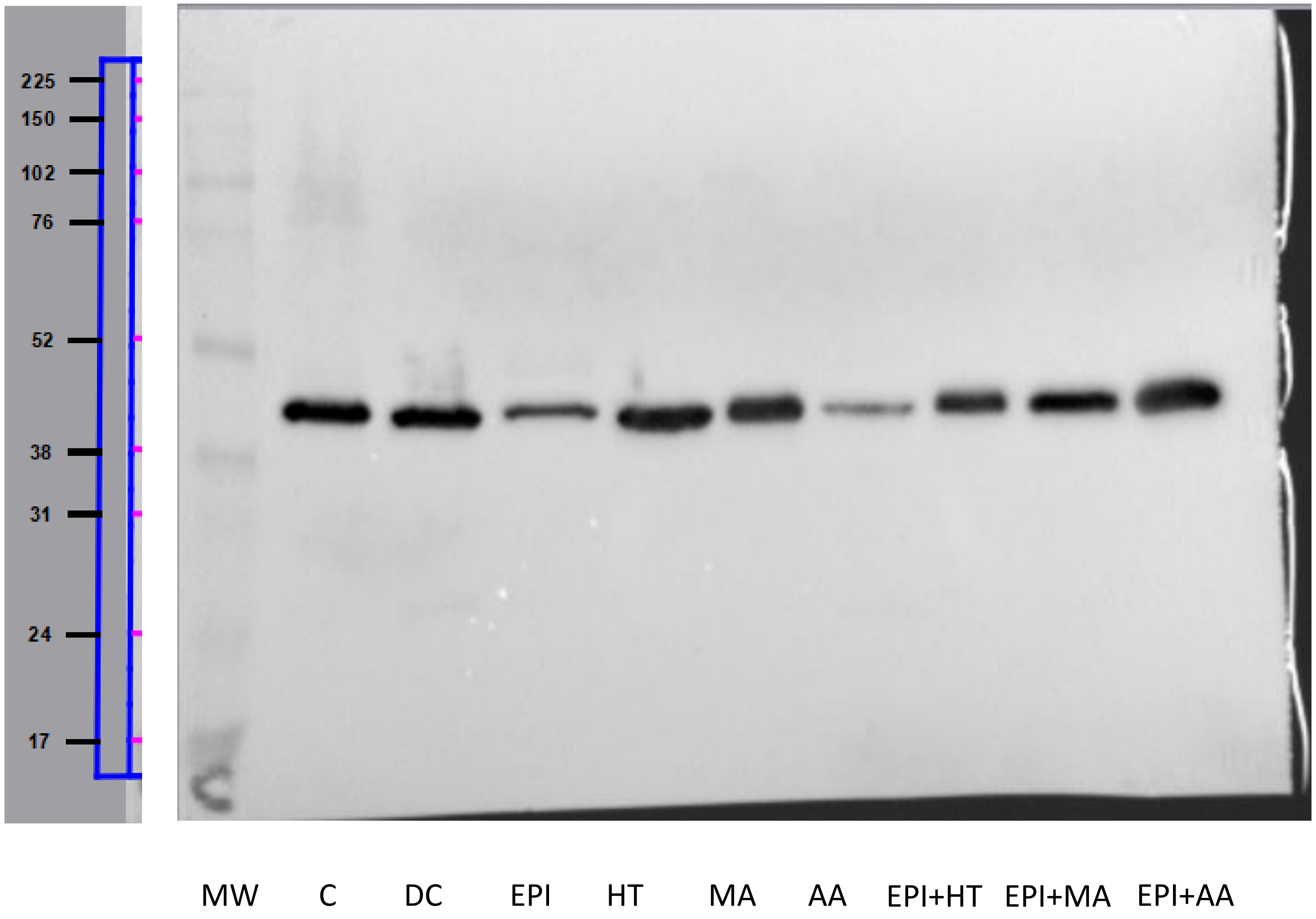

GST-Phase D

Biorad Image lab, (Software 6.1 Windows)

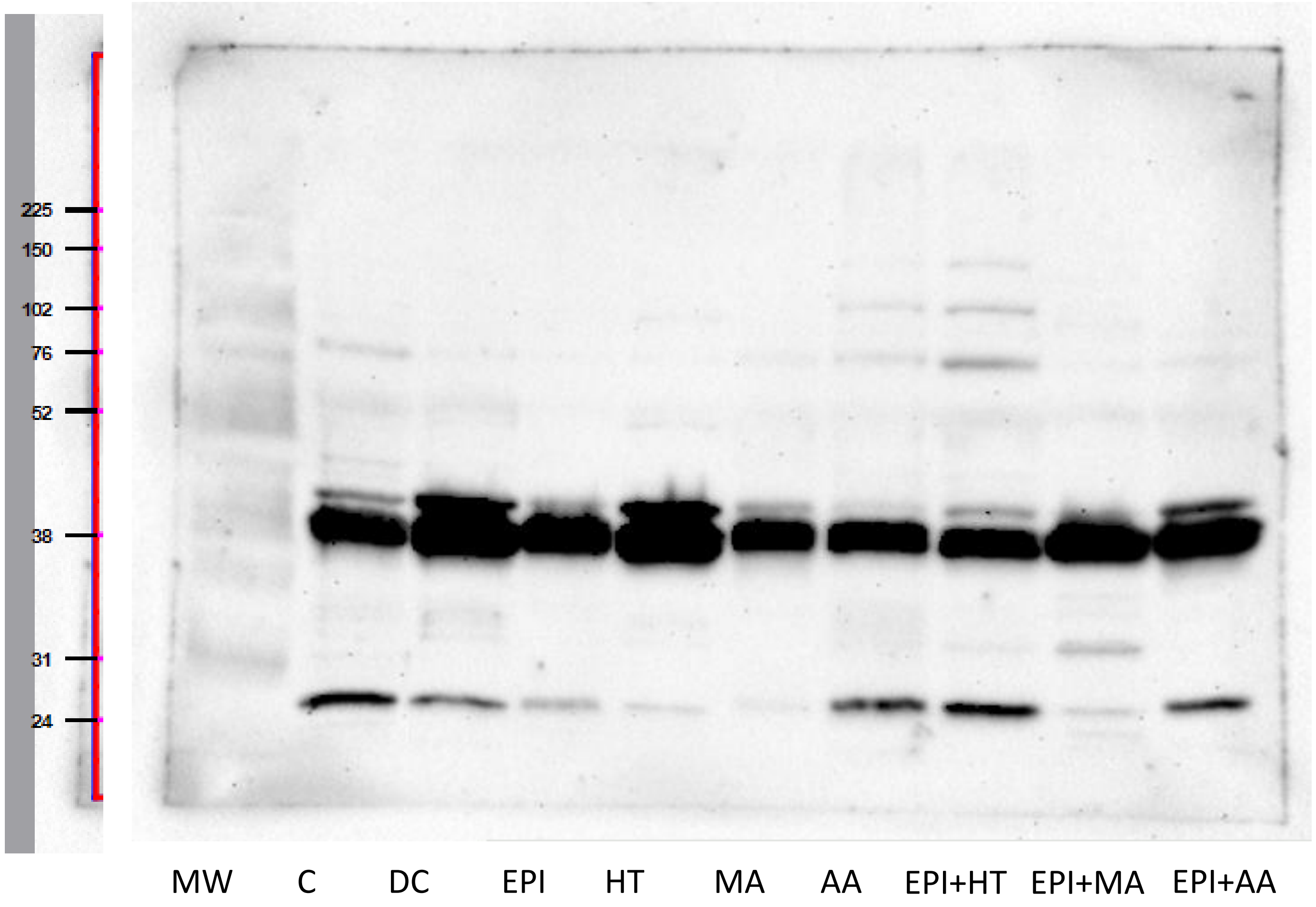

ACTIN (GST)-Phase D

Biorad Image lab, (Software 6.1 Windows)

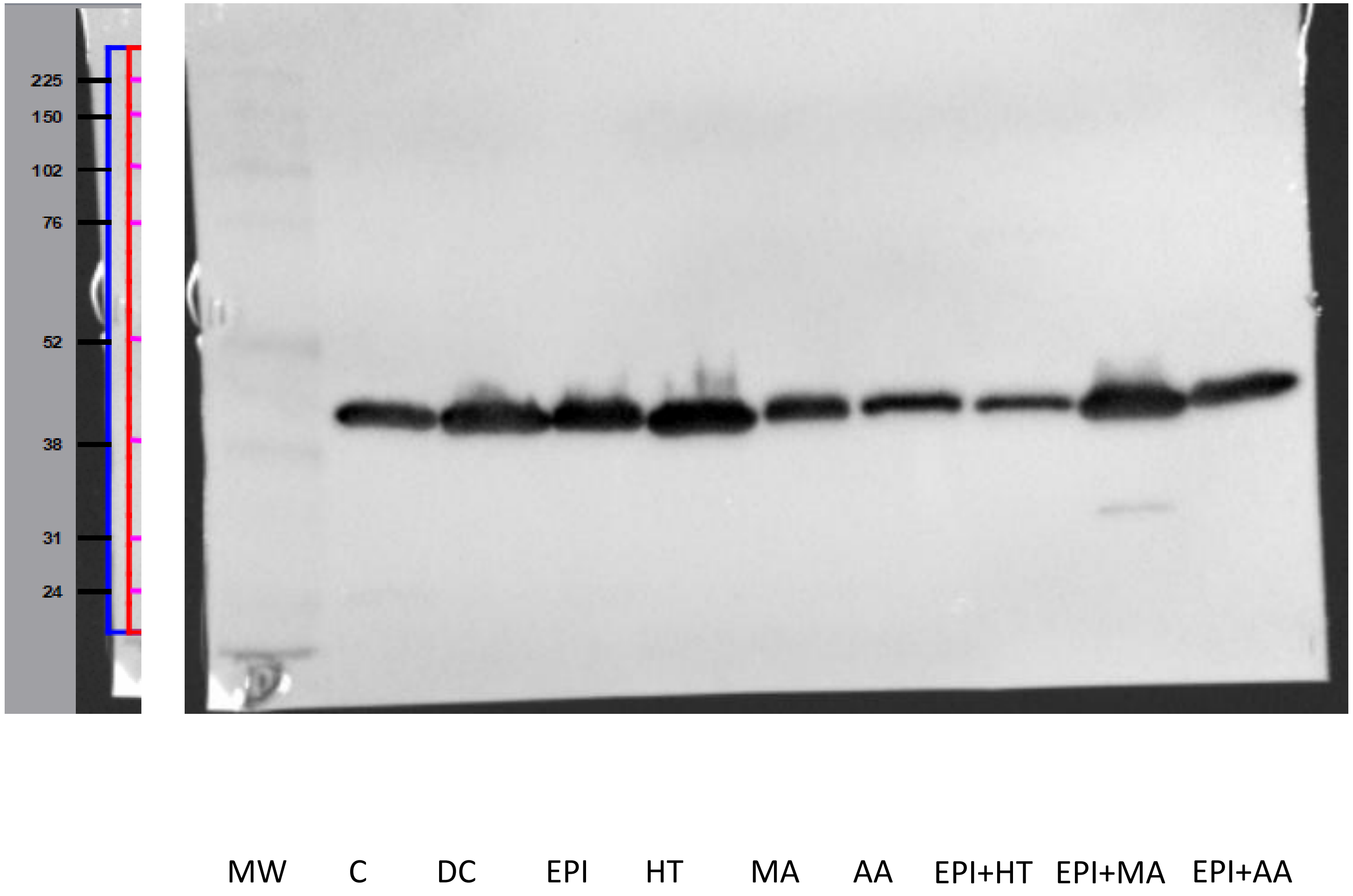

HSP60-Phase A

Biorad Image lab, (Software 6.1 Windows)

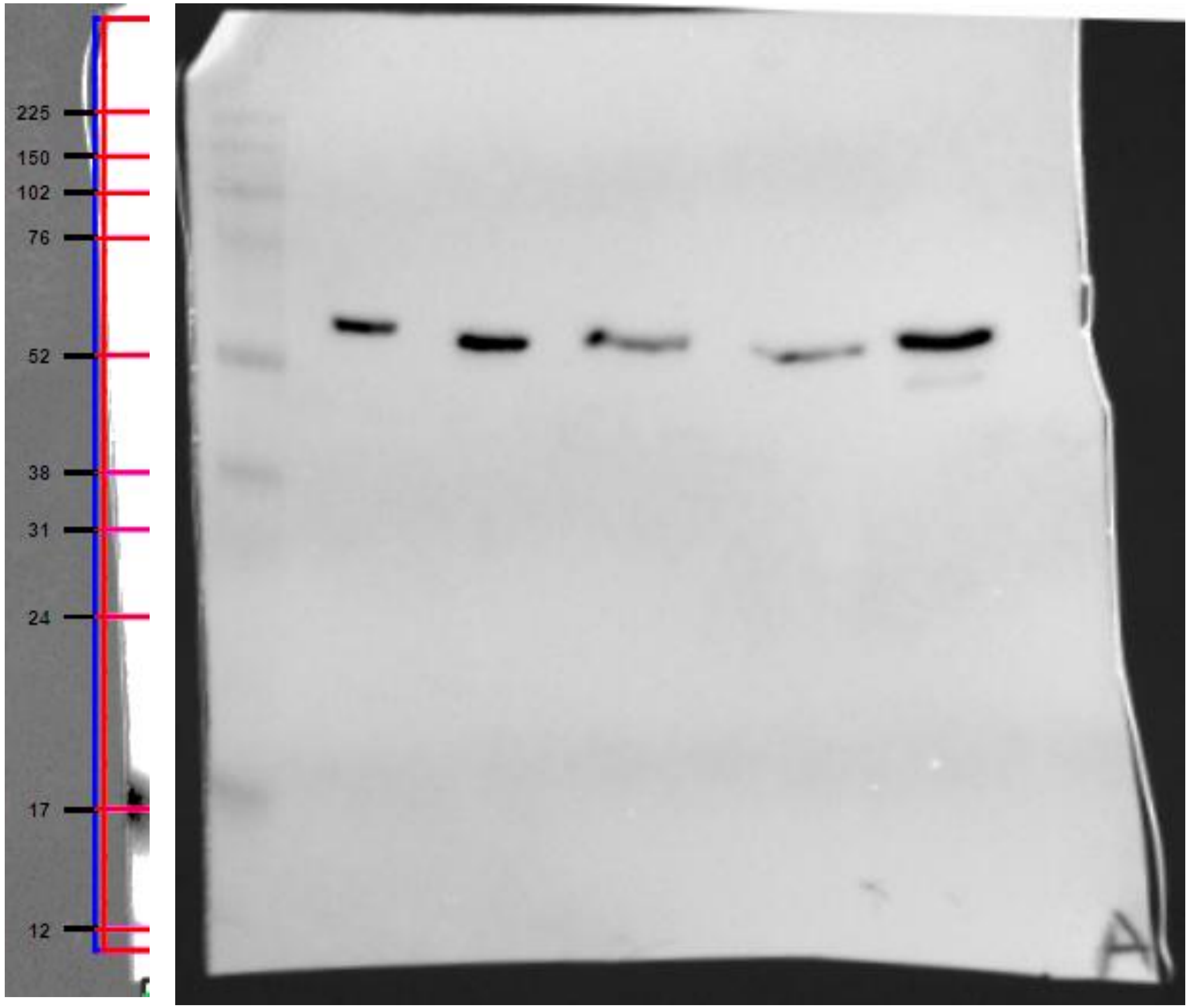

MW C DC HT MA AA

ACTIN (HSP60)-Phase A

Biorad Image lab, (Software 6.1 Windows)

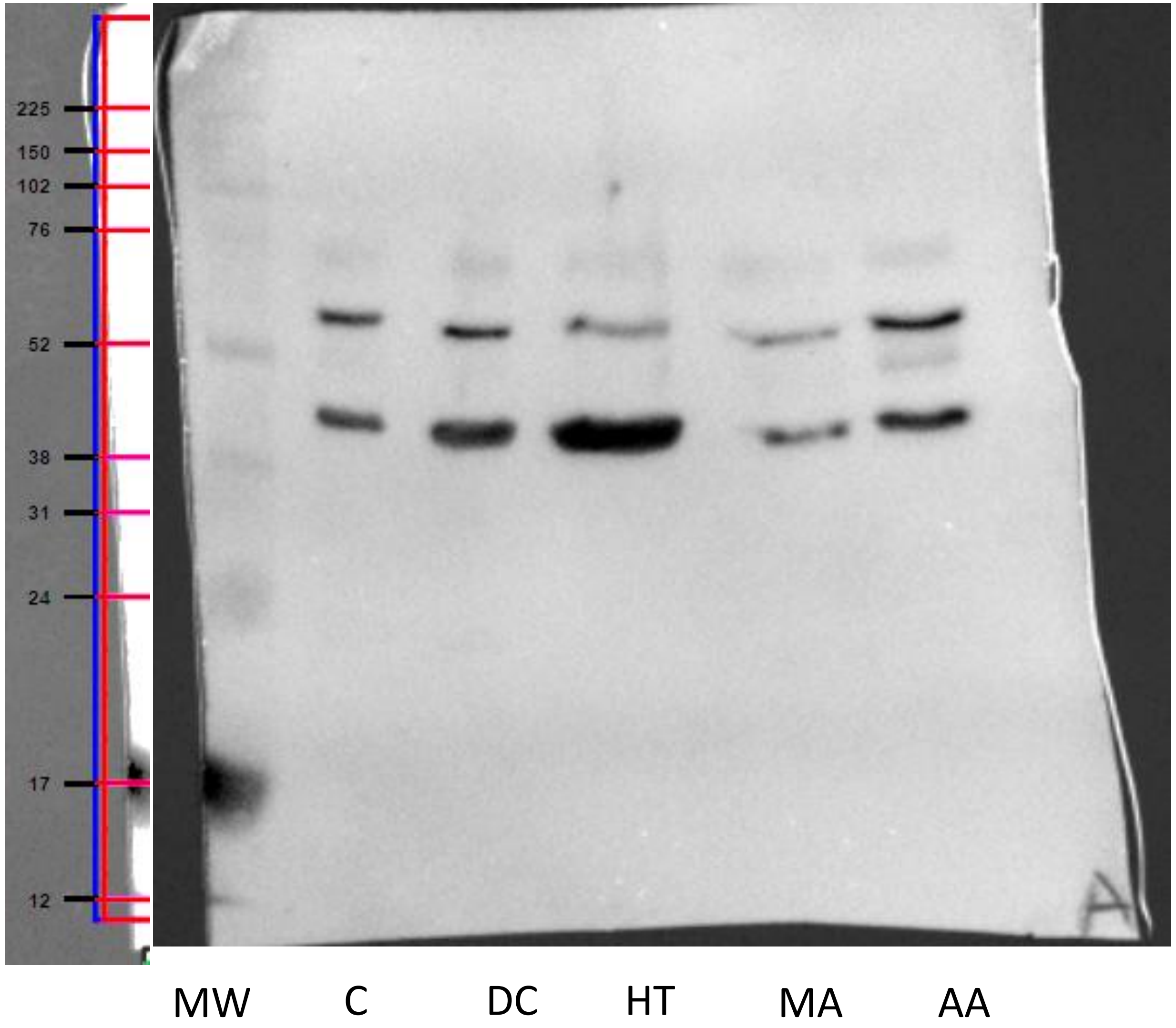

HSP60-Phase B

Biorad Image lab, (Software 6.1 Windows)

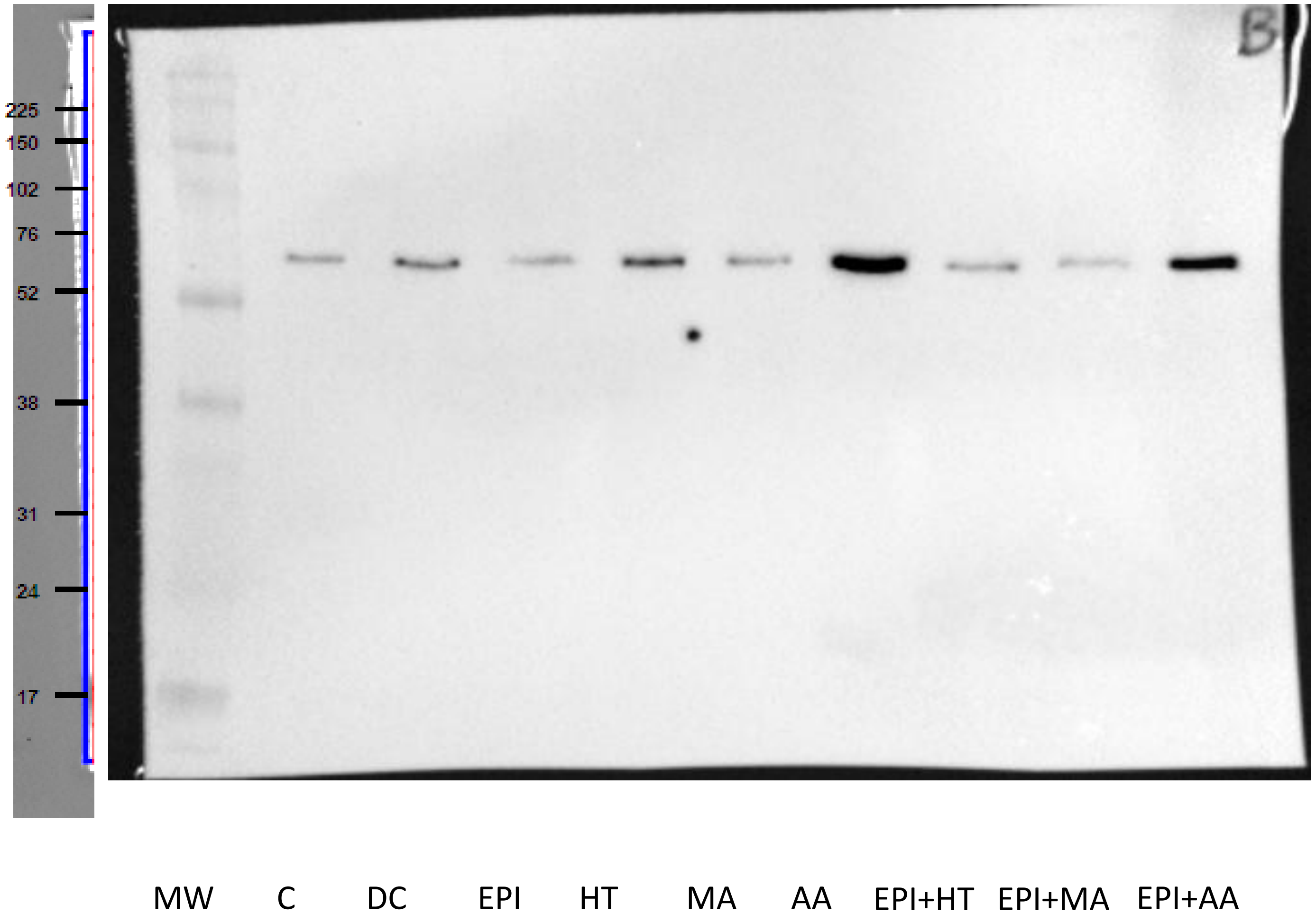

ACTIN (HSP60)-Phase B

Biorad Image lab, (Software 6.1 Windows)

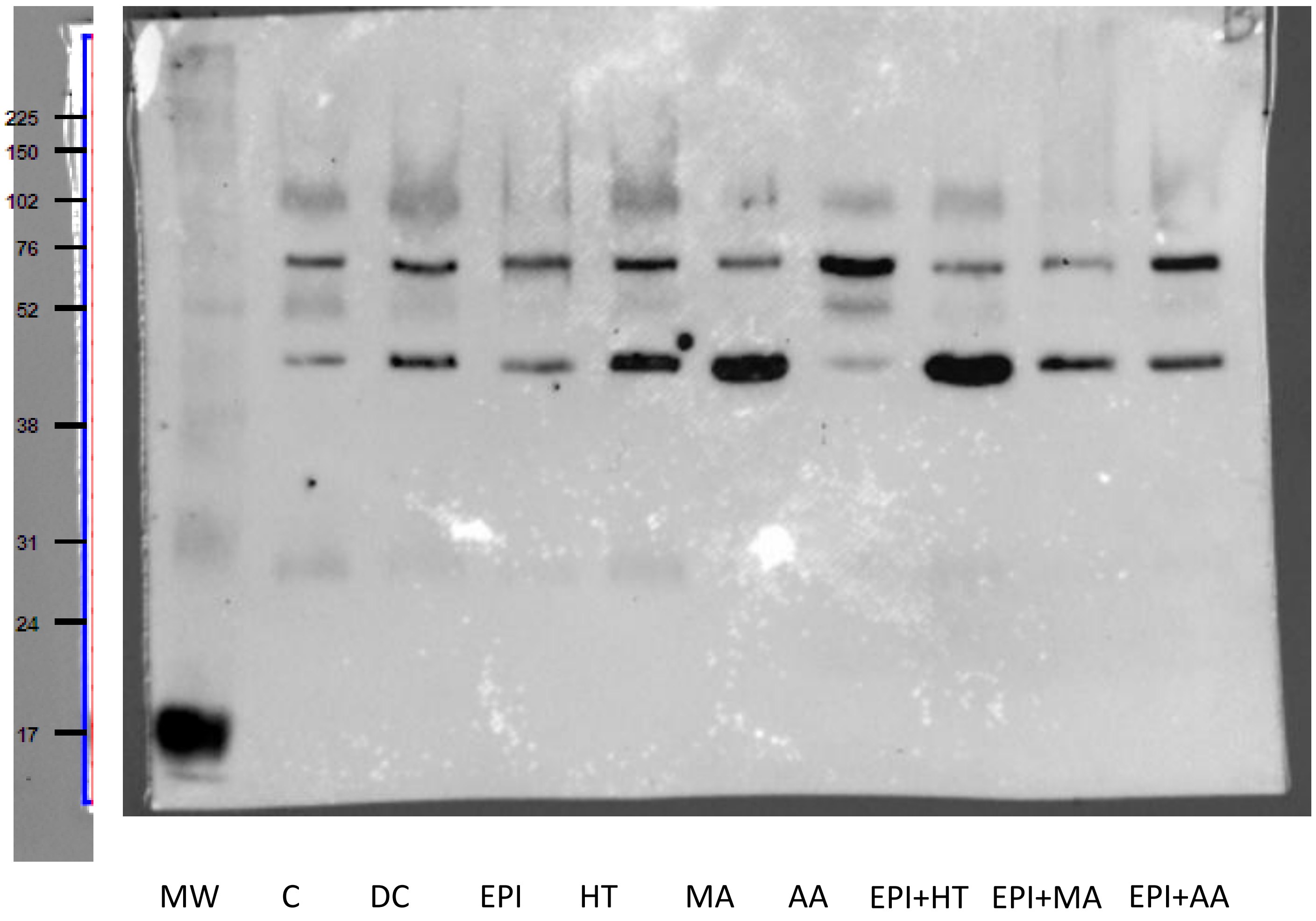

HSP60-Phase C

Biorad Image lab, (Software 6.1 Windows)

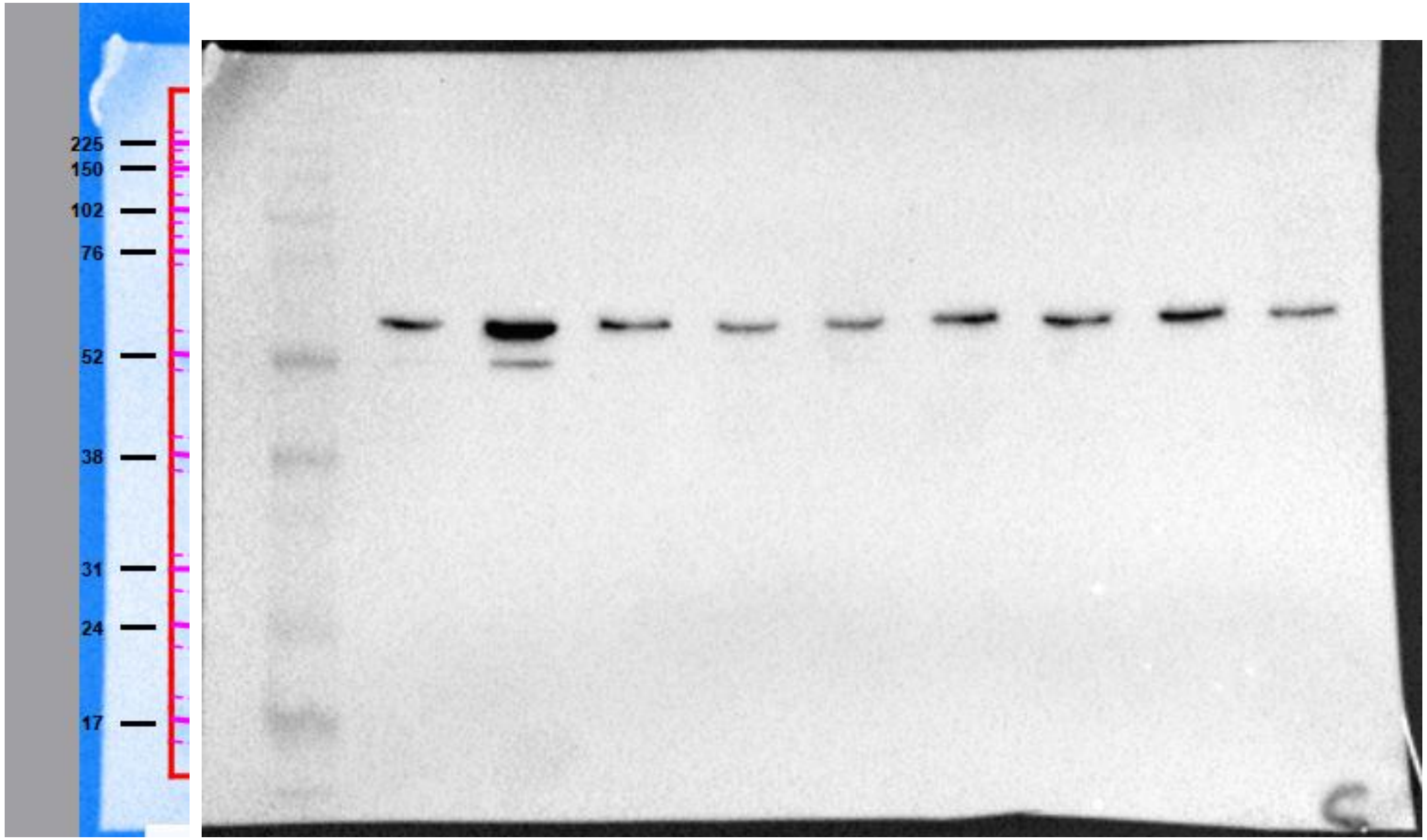

MW C DC EPI HT MA AA EPI+HT EPI+MA EPI+AA

ACTIN (HSP60)-Phase C

Biorad Image lab, (Software 6.1 Windows)

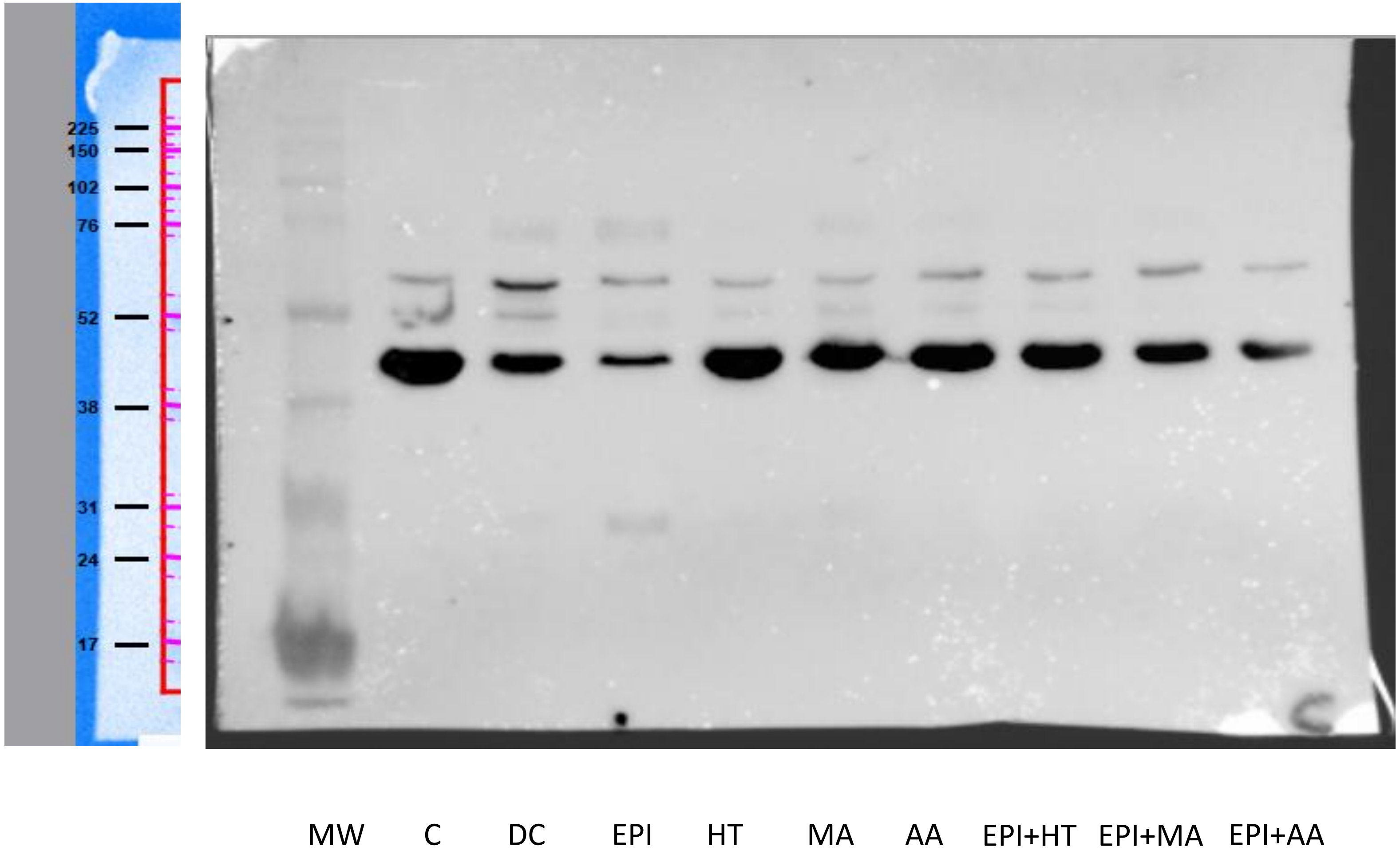

HSP60-Phase D

Biorad Image lab, (Software 6.1 Windows)

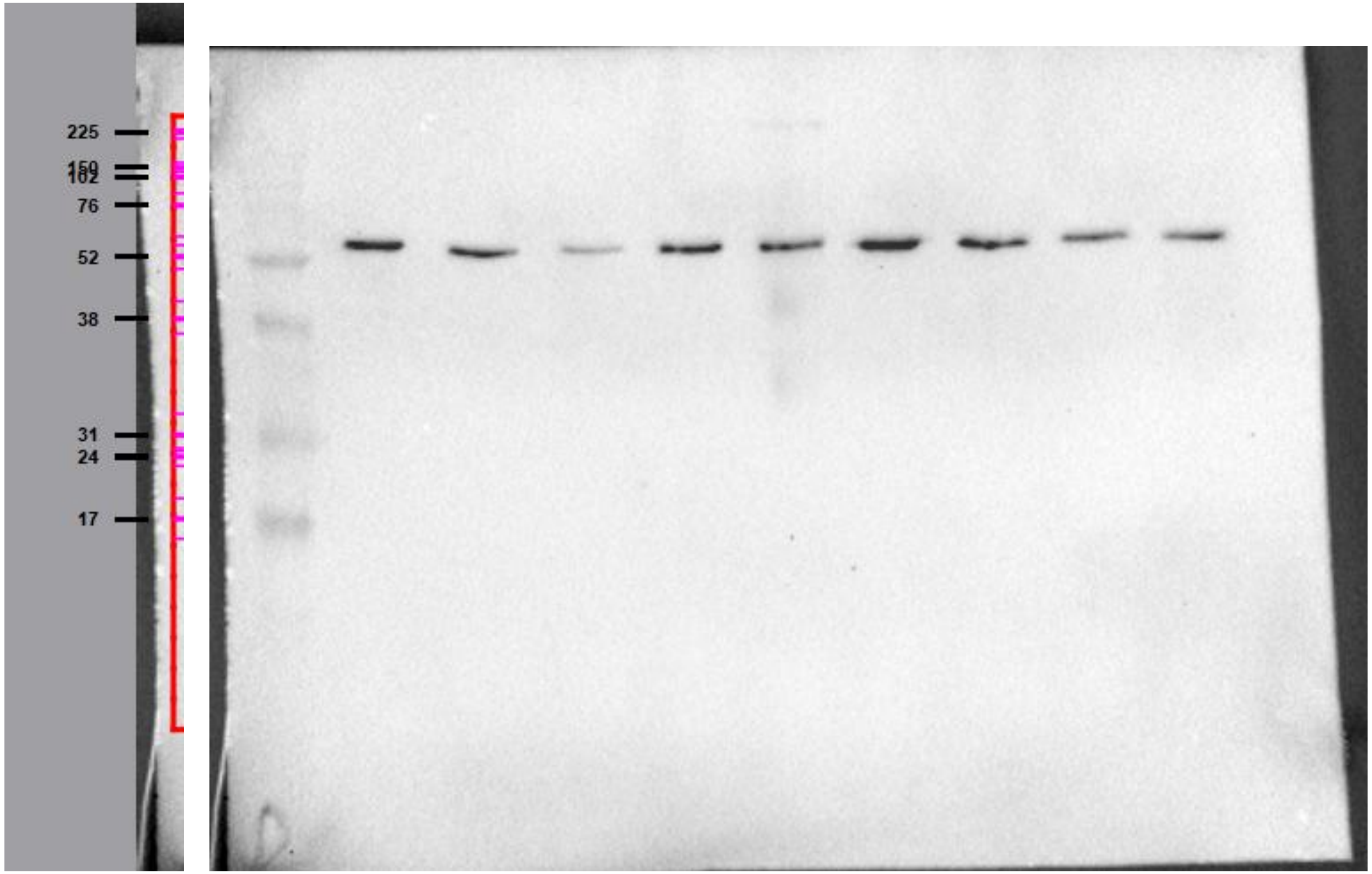

MW C DC EPI HT MA AA EPI+HT EPI+MA EPI+AA

ACTIN (HSP60)-Phase D

Biorad Image lab, (Software 6.1 Windows)

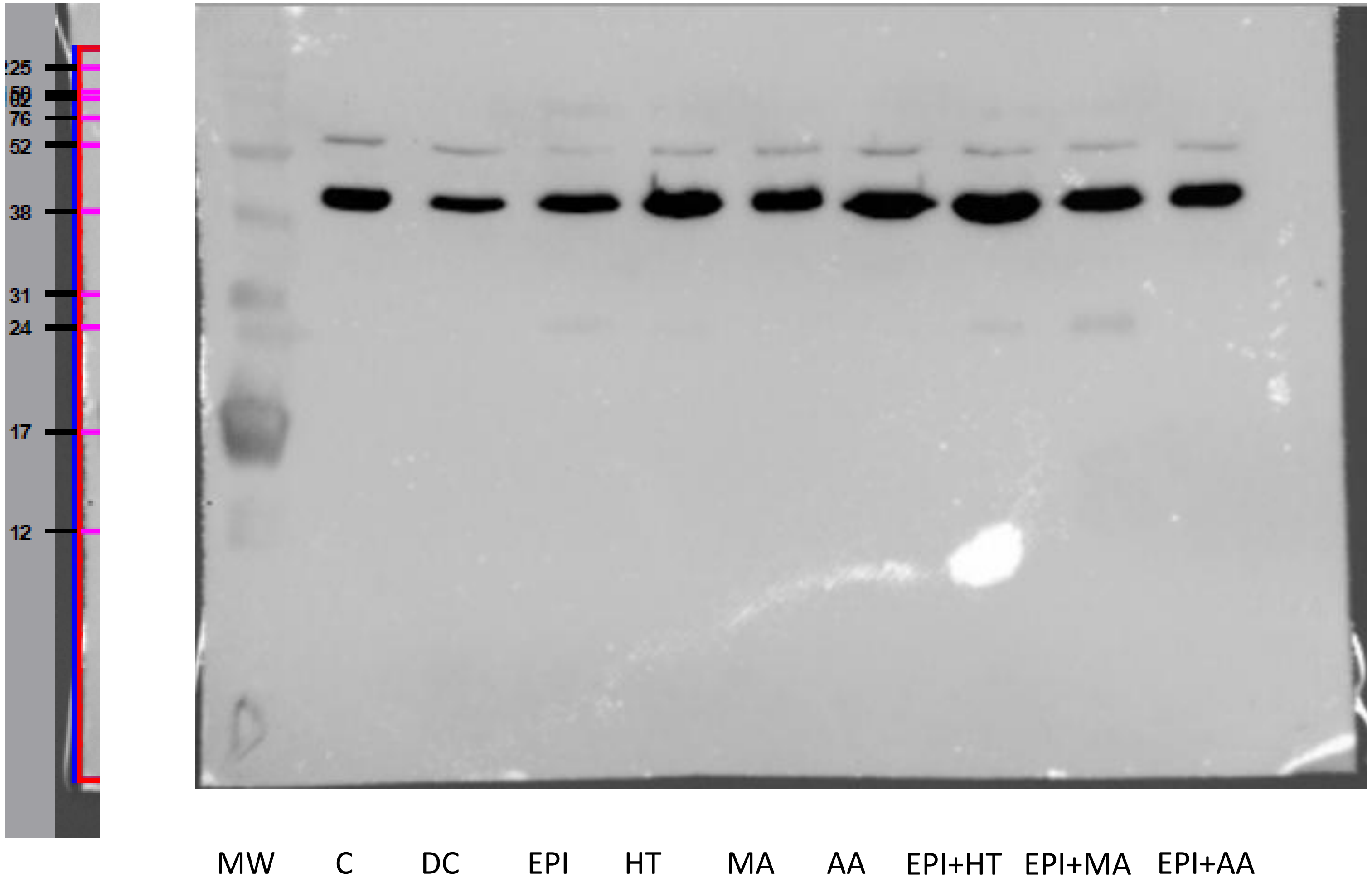

JNK-Phase A

Biorad Image lab, (Software 6.1 Windows)

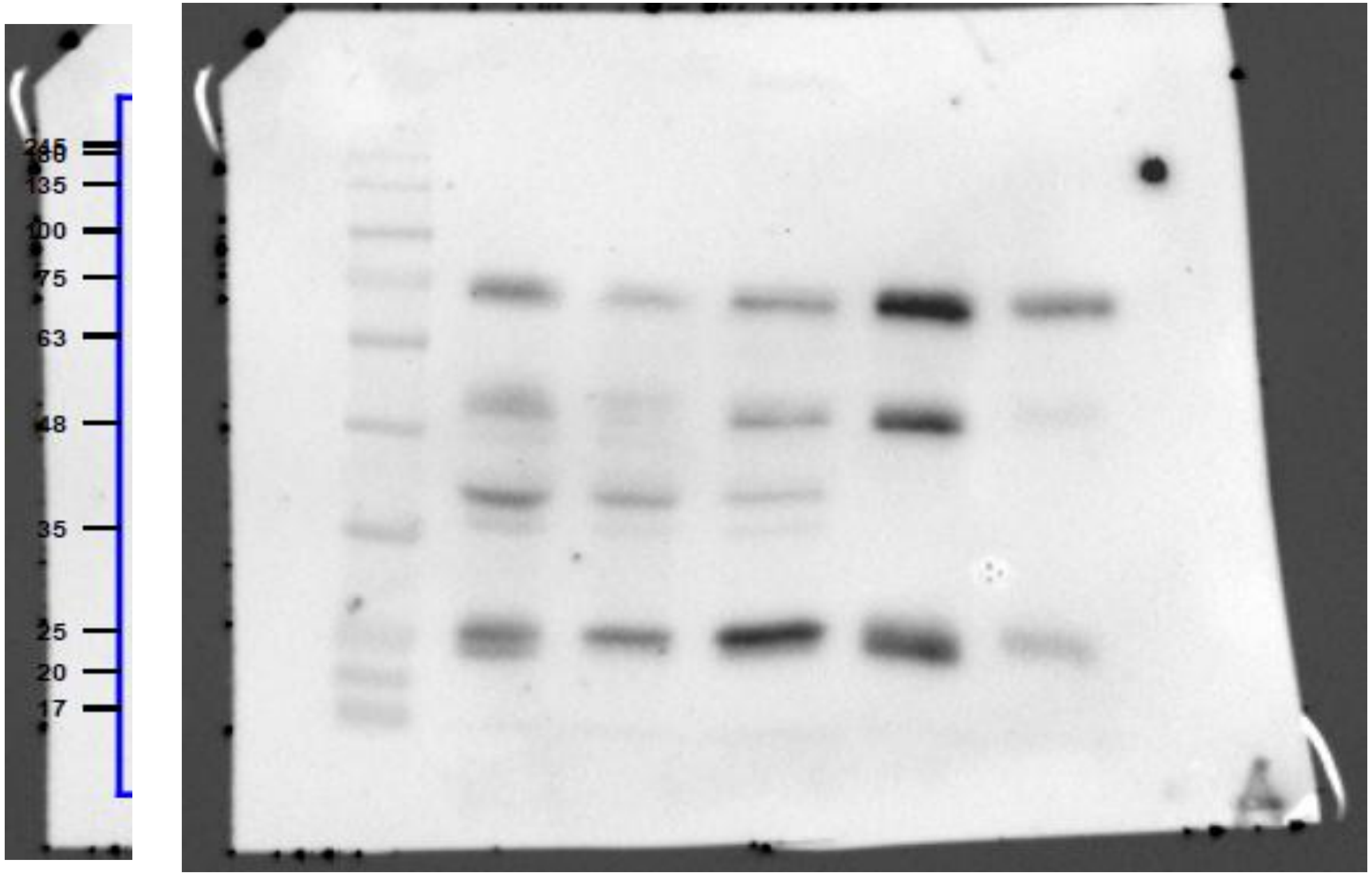

MW C DC HT MA AA

ACTIN (JNK)-Phase A

Biorad Image lab, (Software 6.1 Windows)

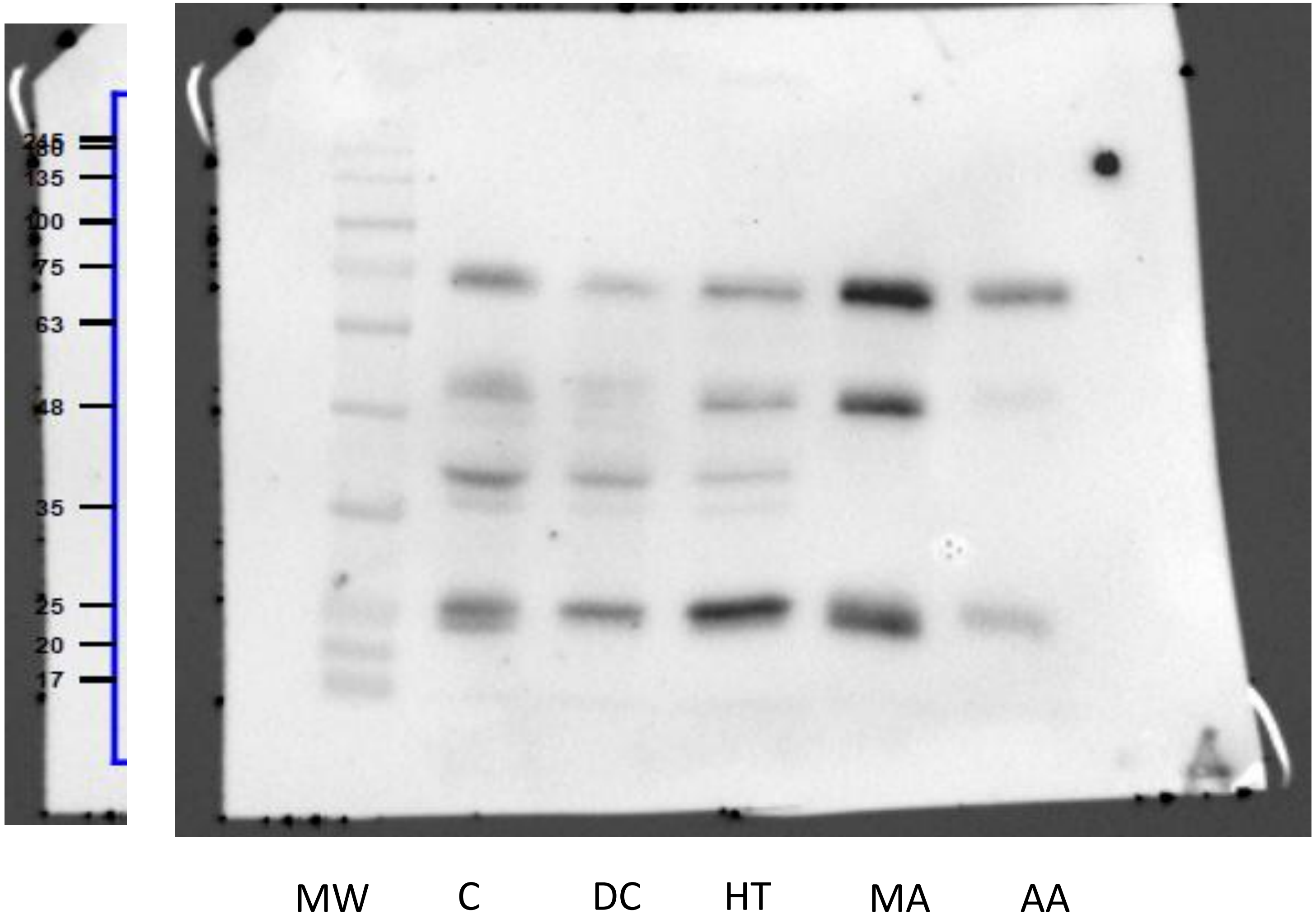

JNK-Phase B

Biorad Image lab, (Software 6.1 Windows)

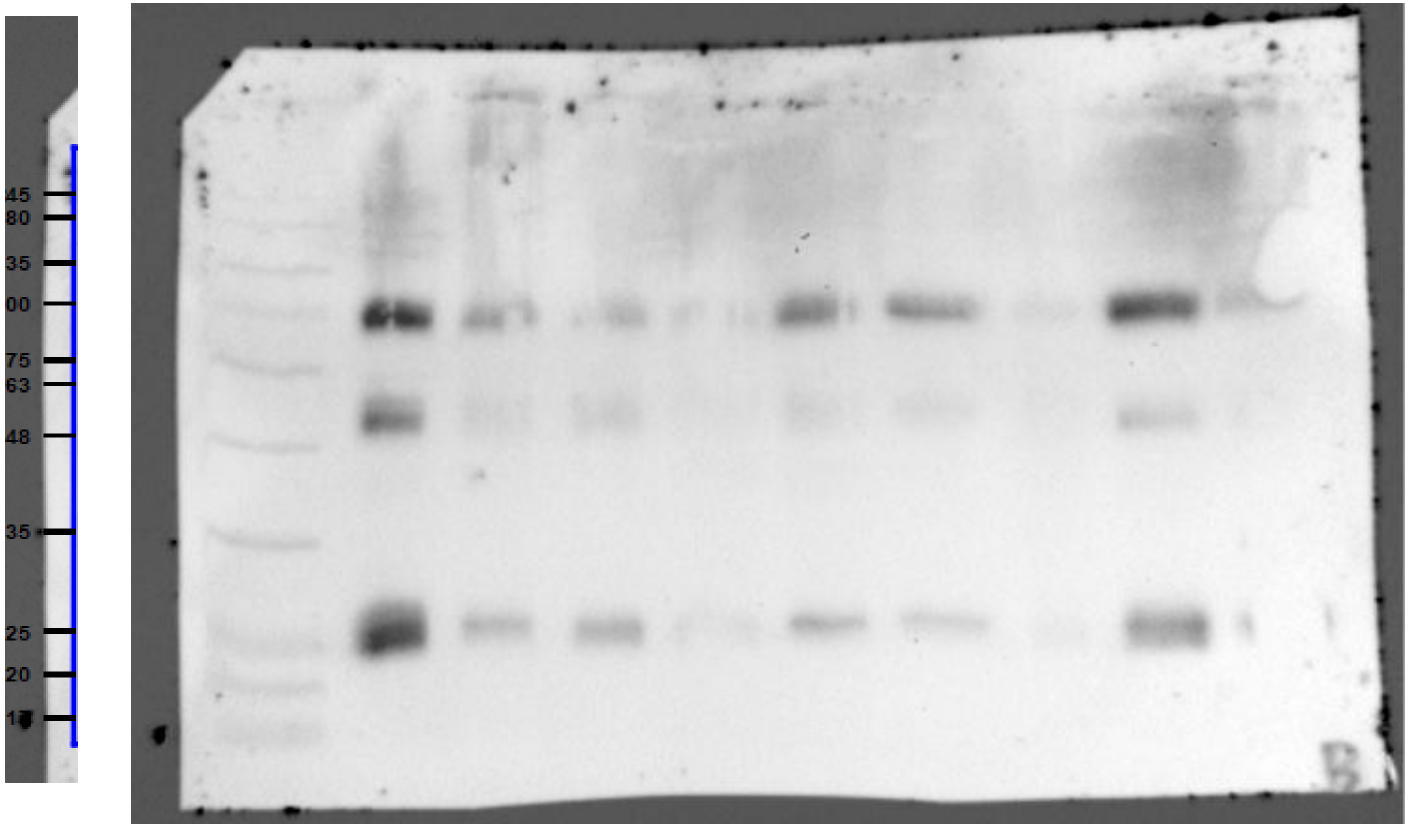

MW C DC EPI HT MA AA EPI+HT EPI+MA EPI+AA

ACTIN (JNK)-Phase B

Biorad Image lab, (Software 6.1 Windows)

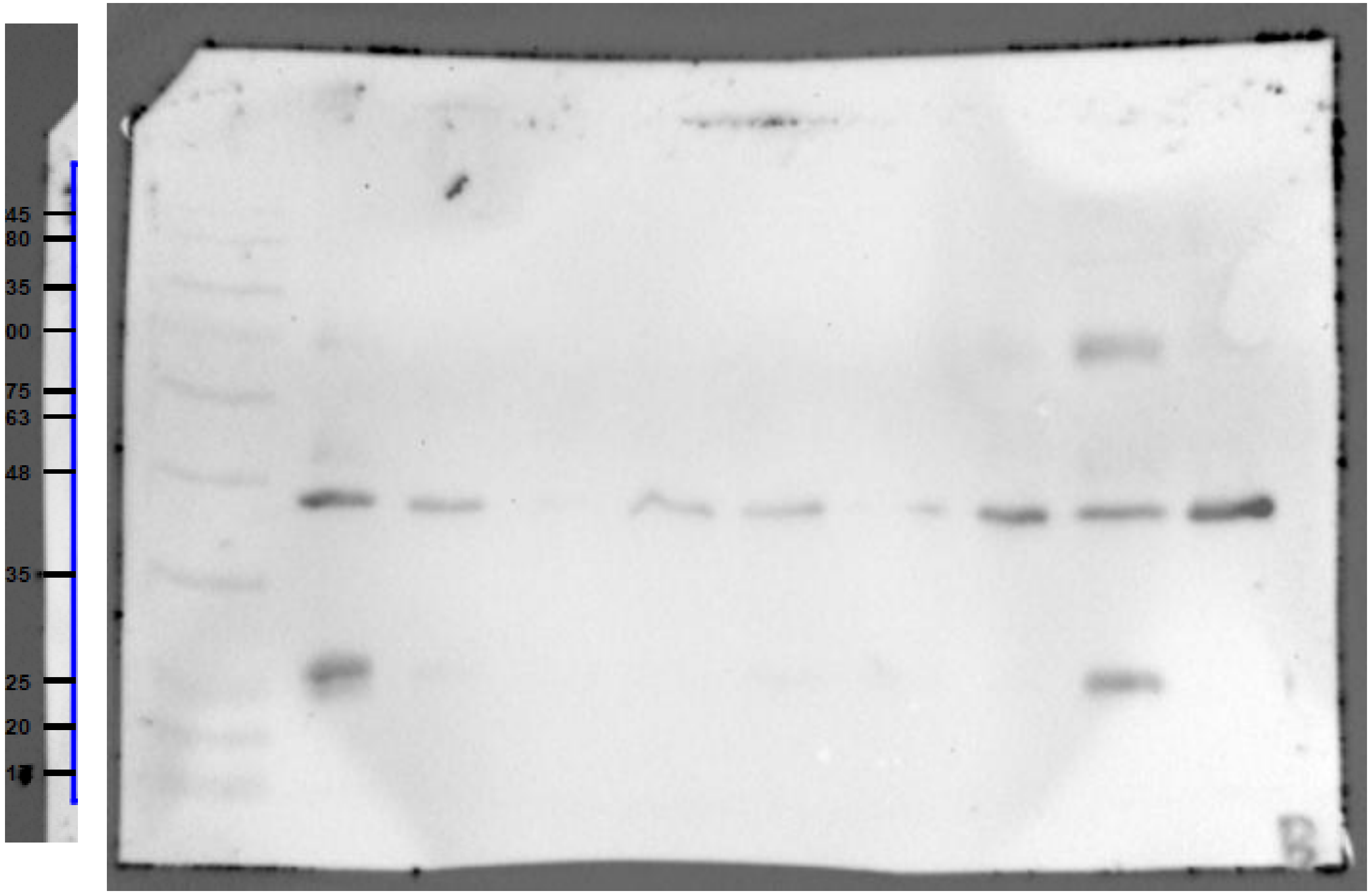

MW C DC EPI HT MA AA EPI+HT EPI+MA EPI+AA

JNK-Phase C

Biorad Image lab, (Software 6.1 Windows)

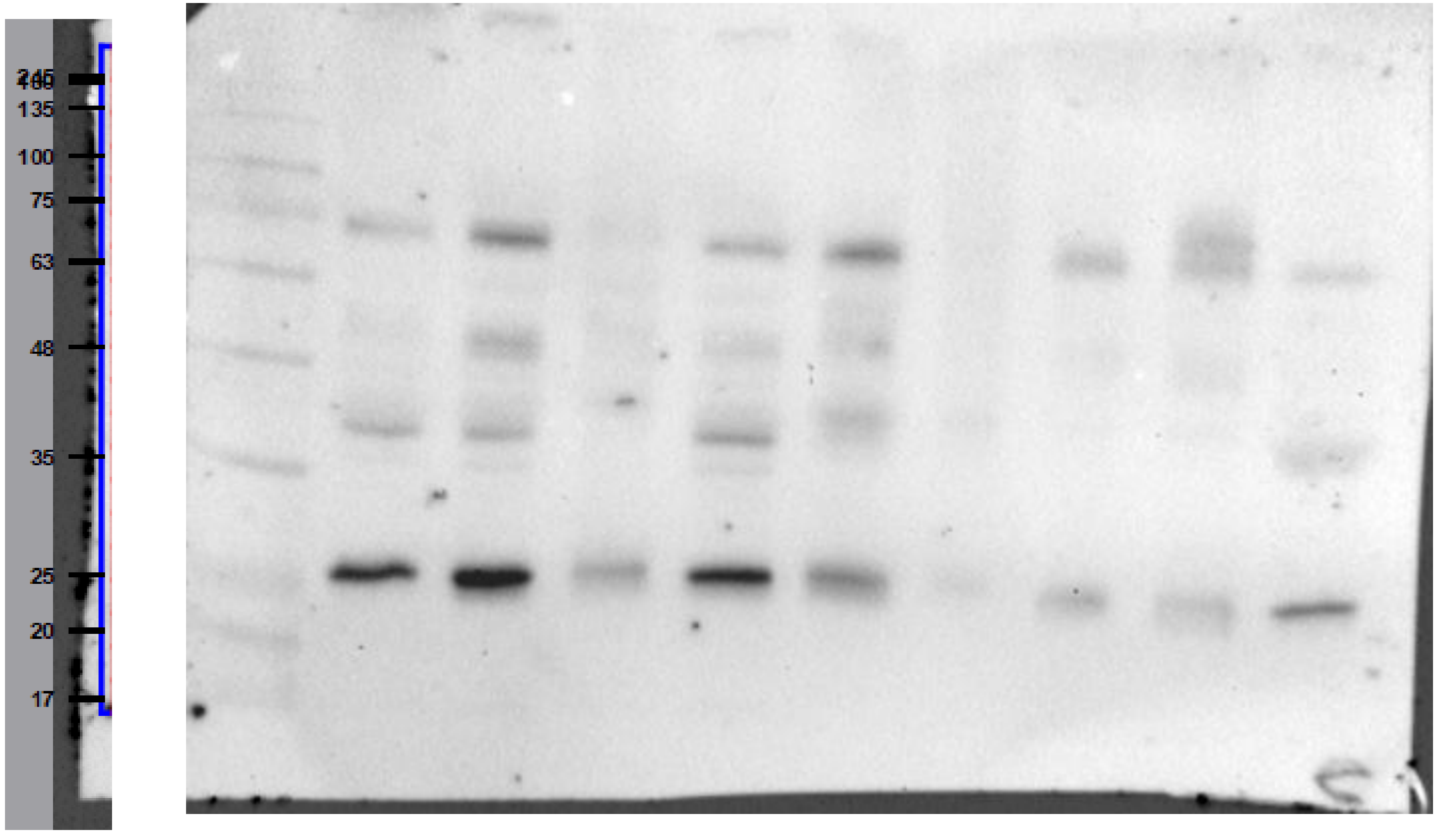

MW C DC EPI HT MA AA EPI+HT EPI+MA EPI+AA

ACTIN (JNK)-Phase C

Biorad Image lab, (Software 6.1 Windows)

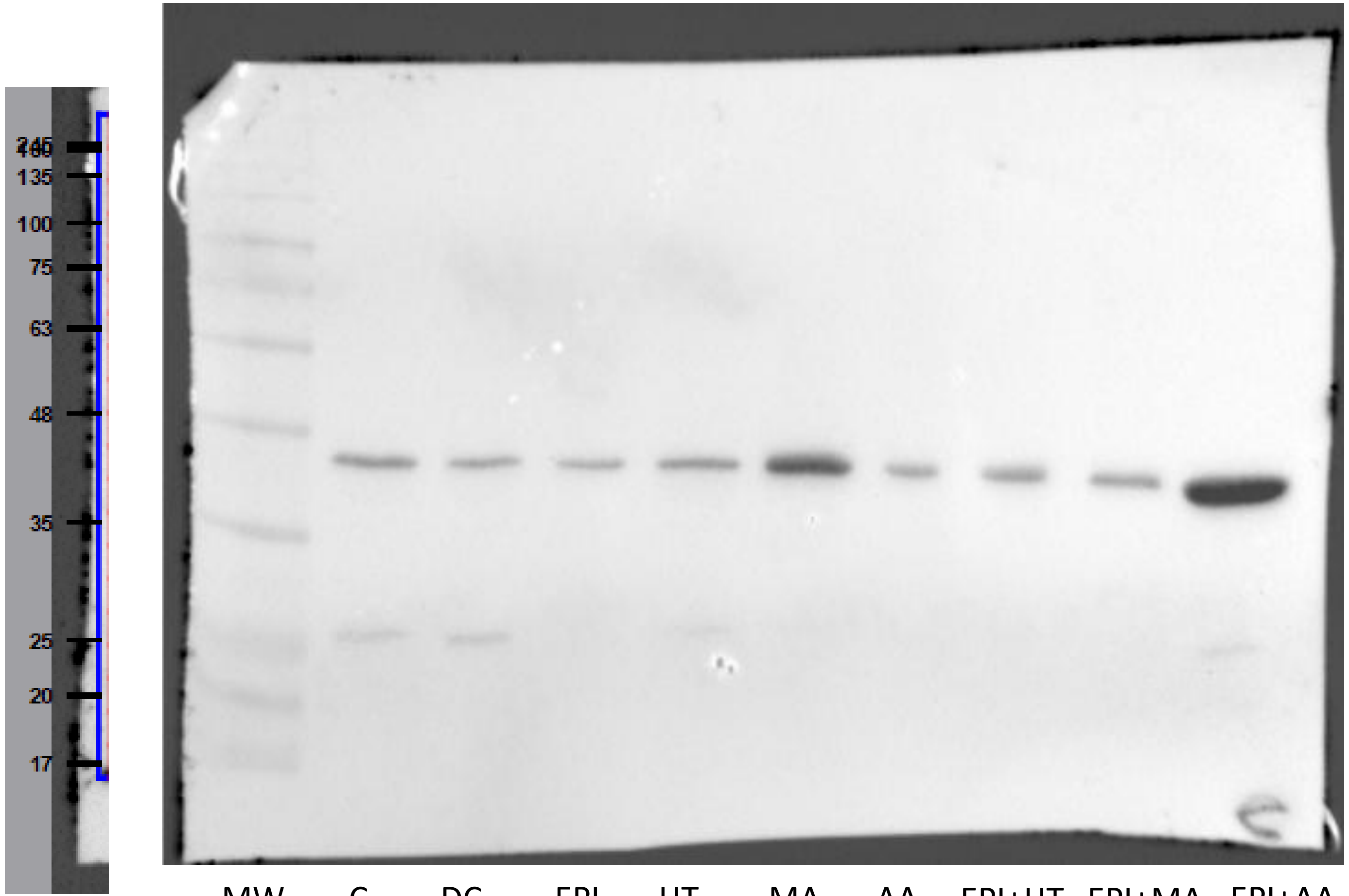

MW C DC EPI HT MA AA EPI+HT EPI+MA EPI+AA

JNK-Phase D

Biorad Image lab, (Software 6.1 Windows)

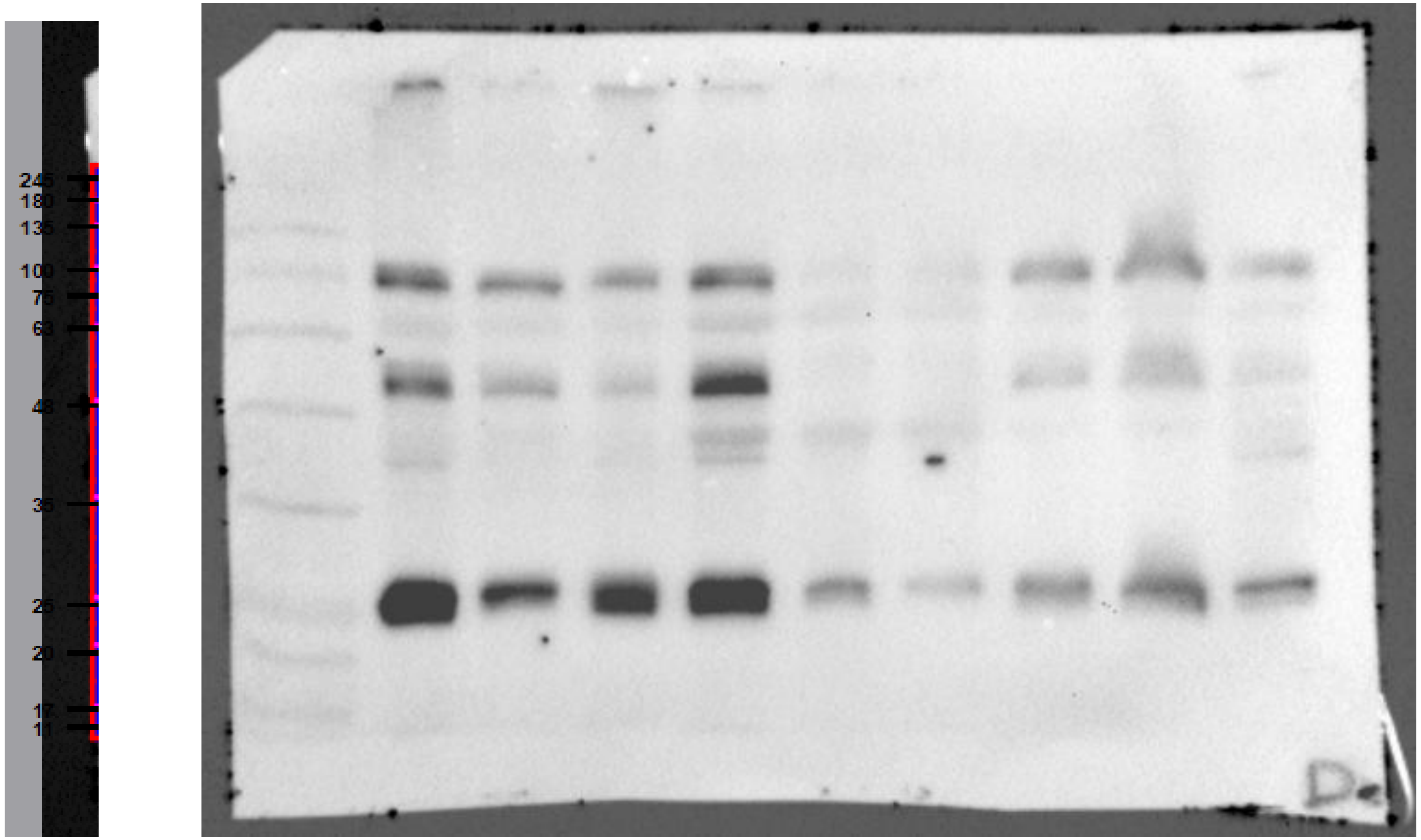

MW C DC EPI HT MA AA EPI+HT EPI+MA EPI+AA

ACTIN (JNK)-Phase D

Biorad Image lab, (Software 6.1 Windows)

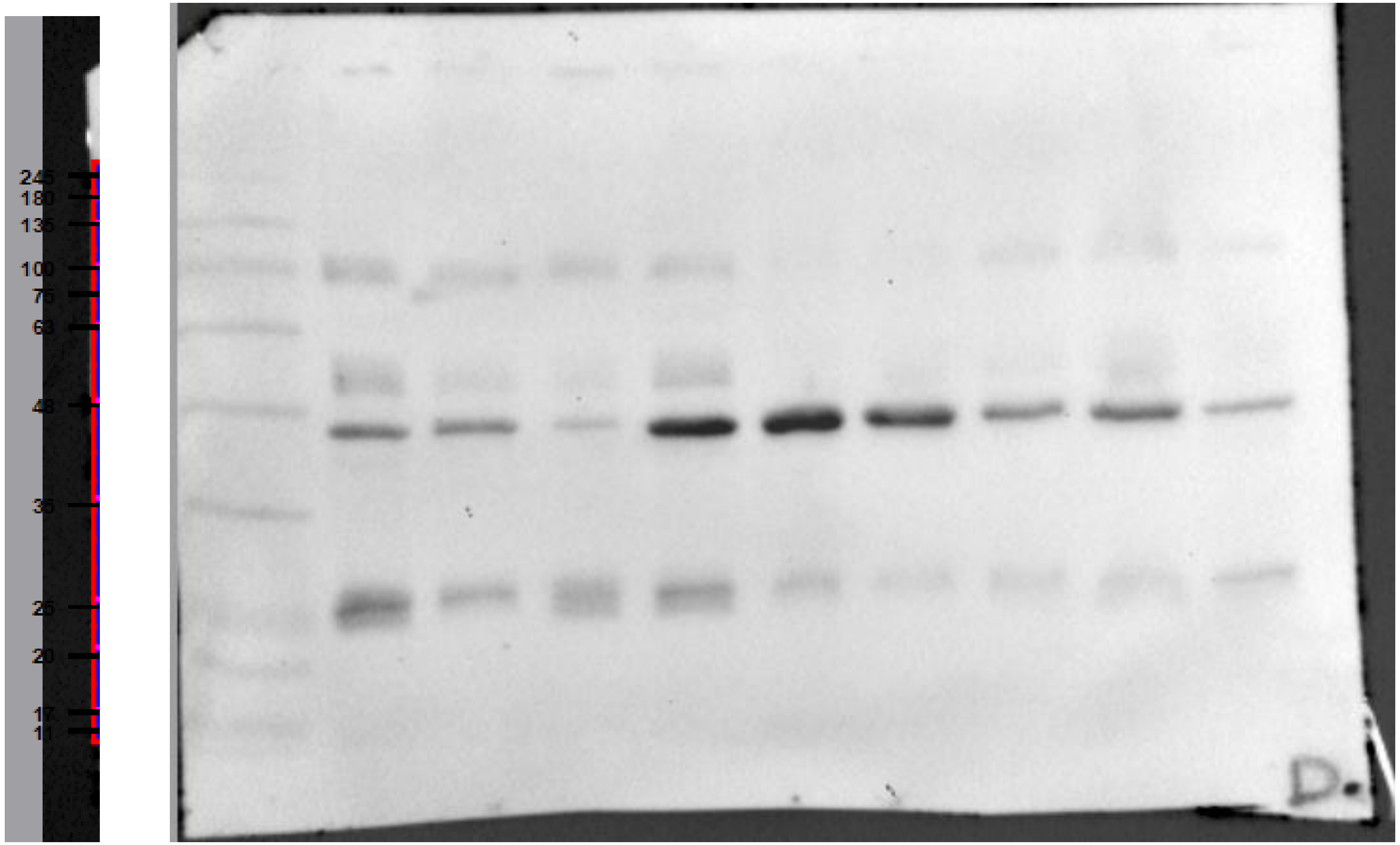

MW C DC EPI HT MA AA EPI+HT EPI+MA EPI+AA

NFkB-Phase A

Biorad Image lab, (Software 6.1 Windows)

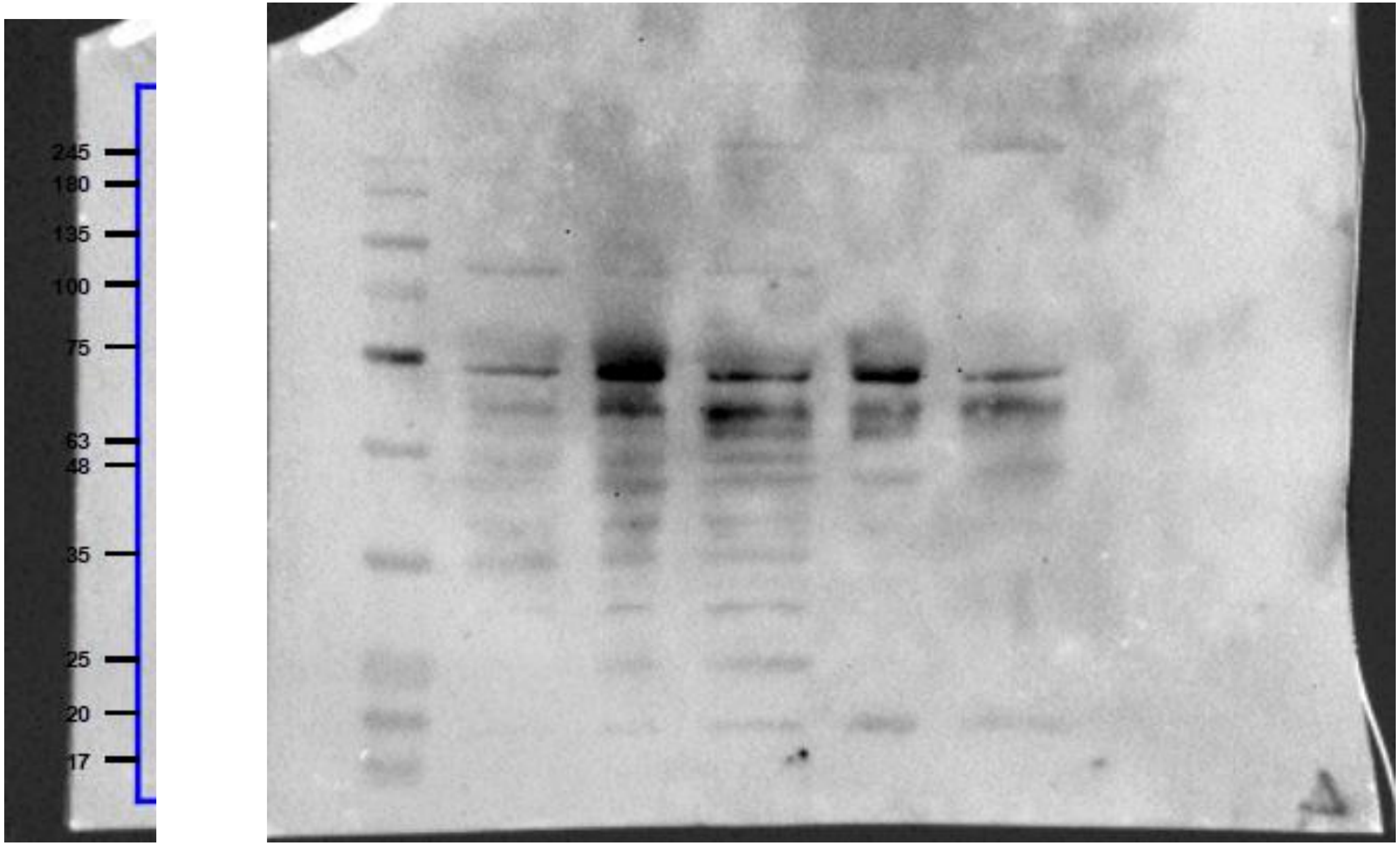

MW C DC HT MA AA

ACTIN (NFkB)-Phase A

Biorad Image lab, (Software 6.1 Windows)

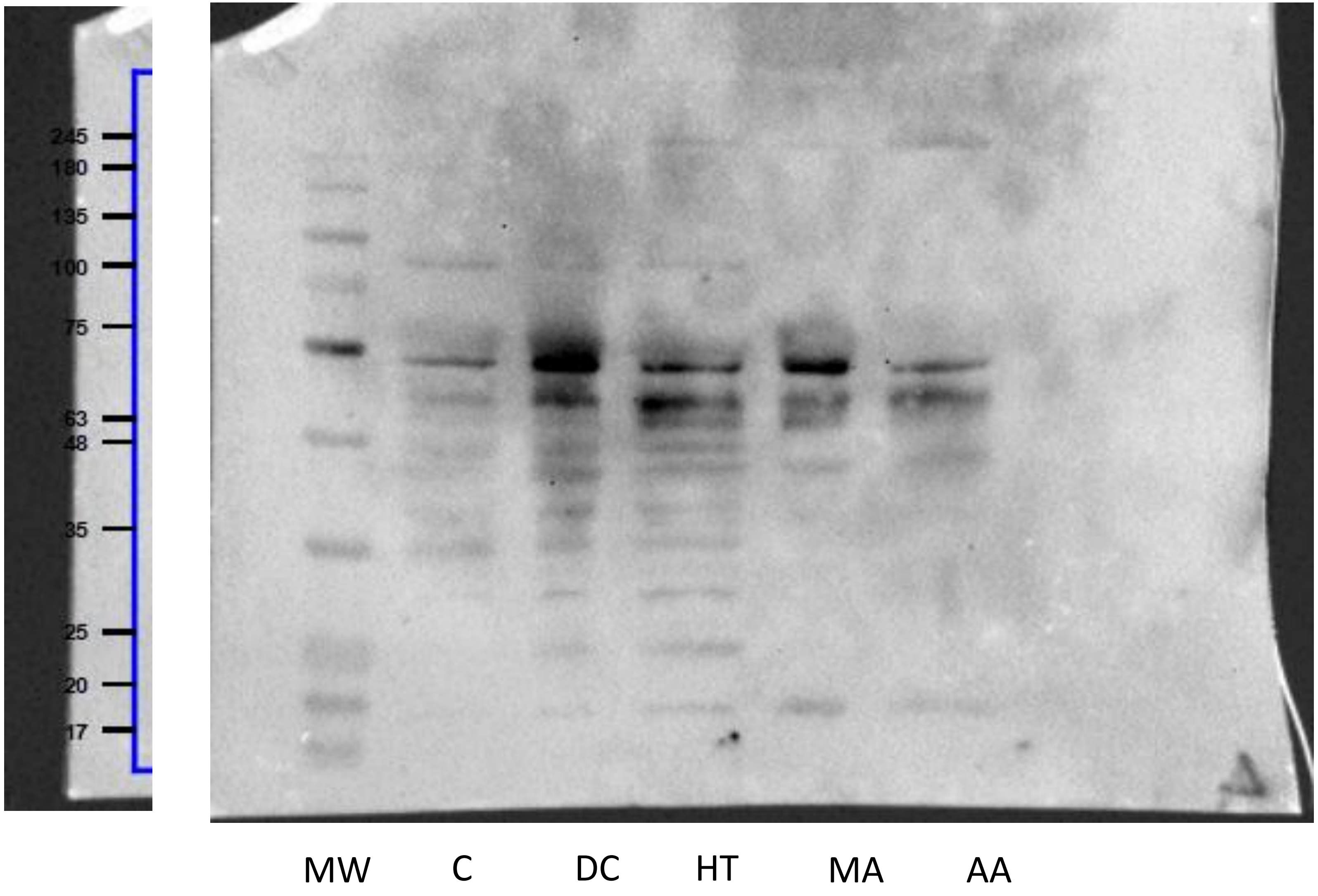

NFkB-Phase B

Biorad Image lab, (Software 6.1 Windows)

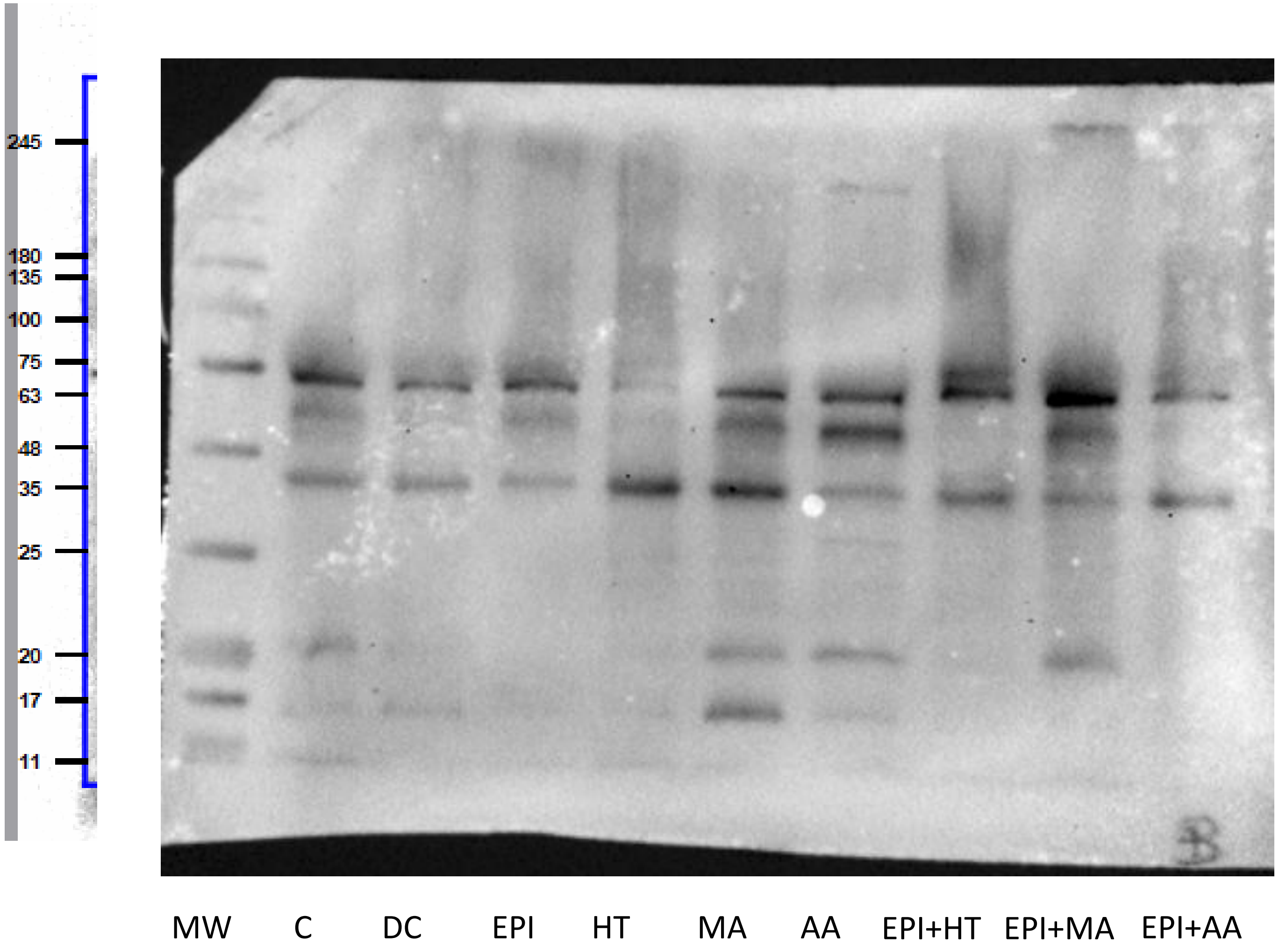

ACTIN (NFkB)-Phase B

Biorad Image lab, (Software 6.1 Windows)

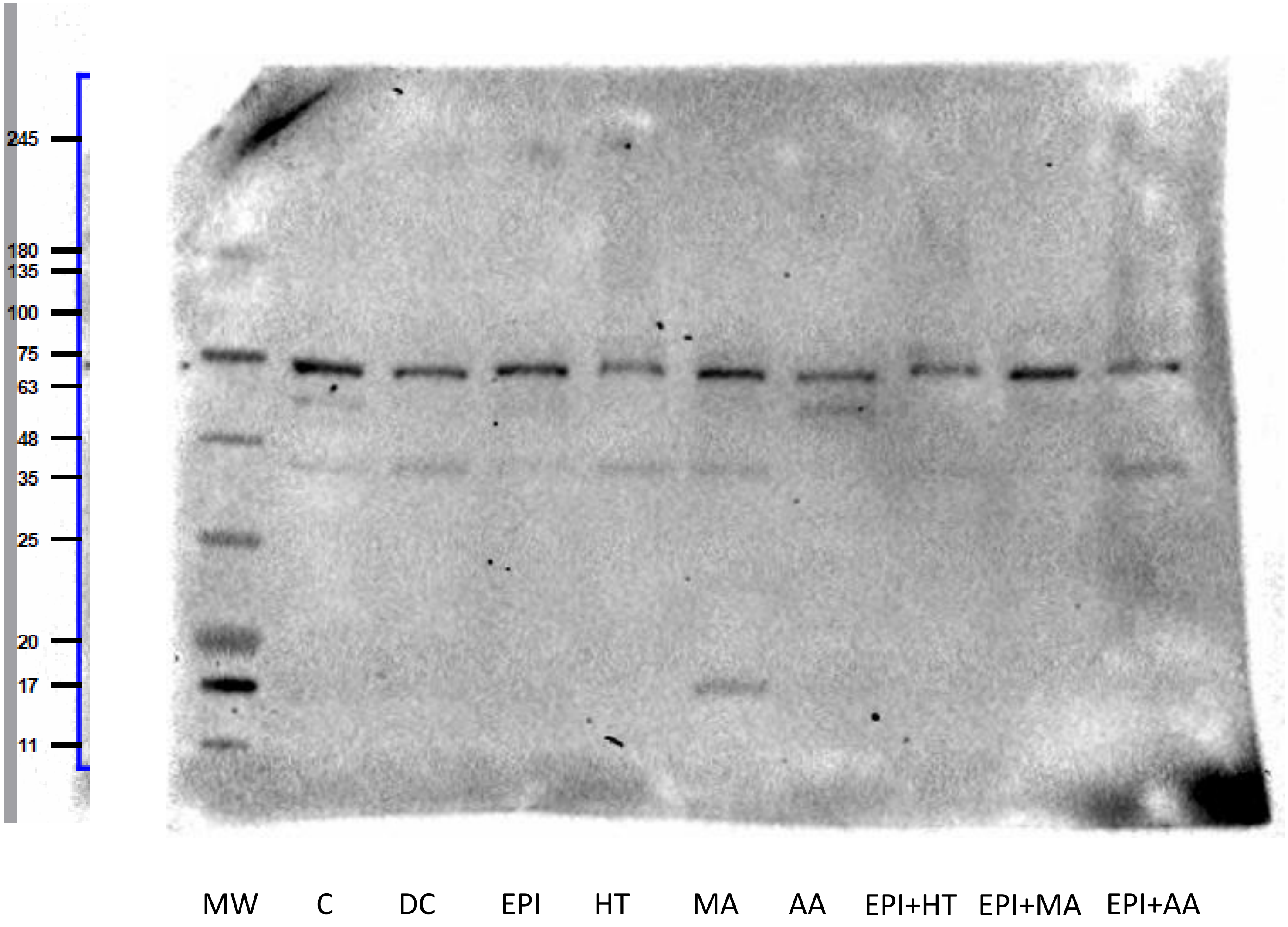

NFkB-Phase C

Biorad Image lab, (Software 6.1 Windows)

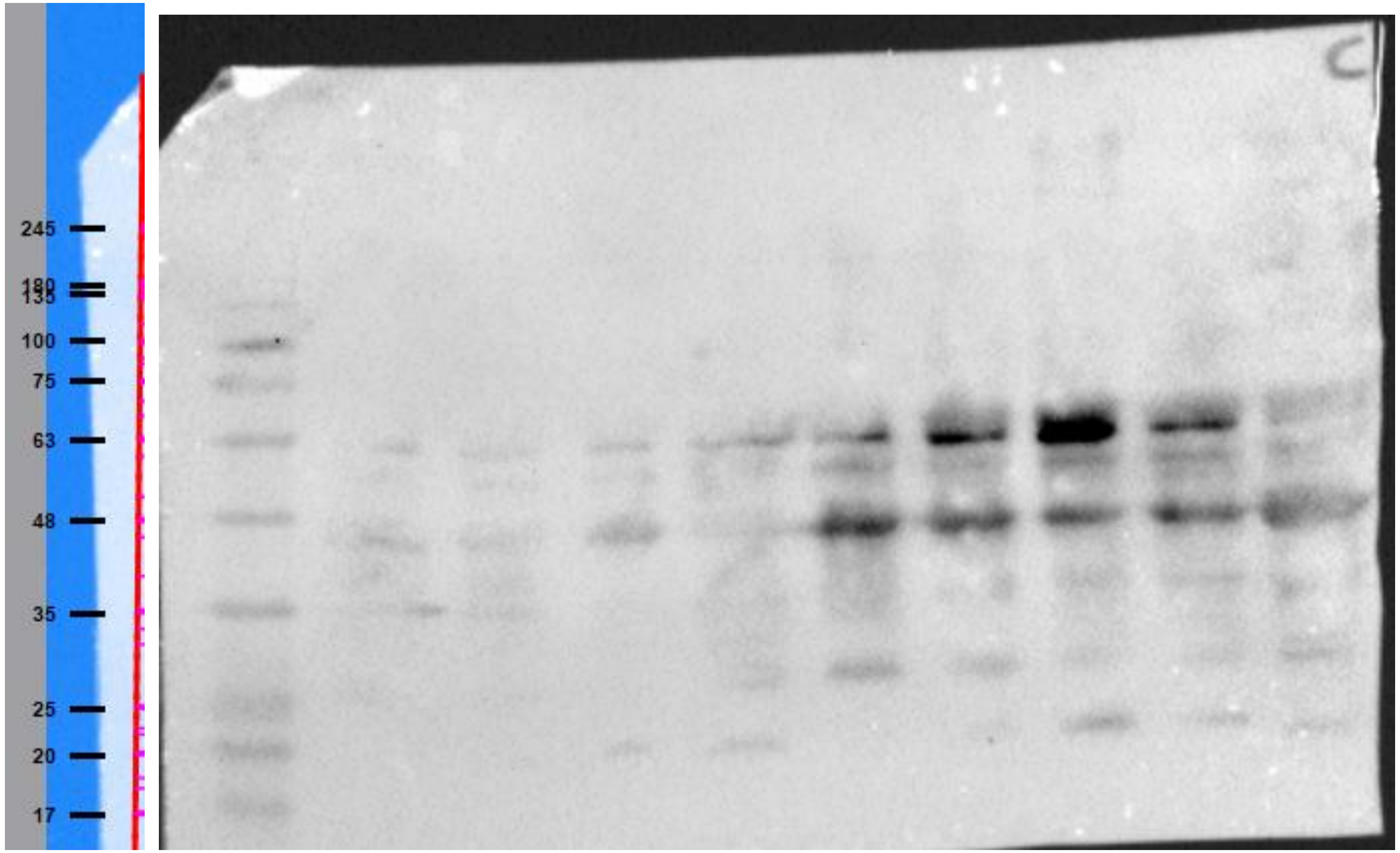

MW C DC EPI HT MA AA EPI+HT EPI+MA EPI+AA

ACTIN (NFkB)-Phase C

Biorad Image lab, (Software 6.1 Windows)

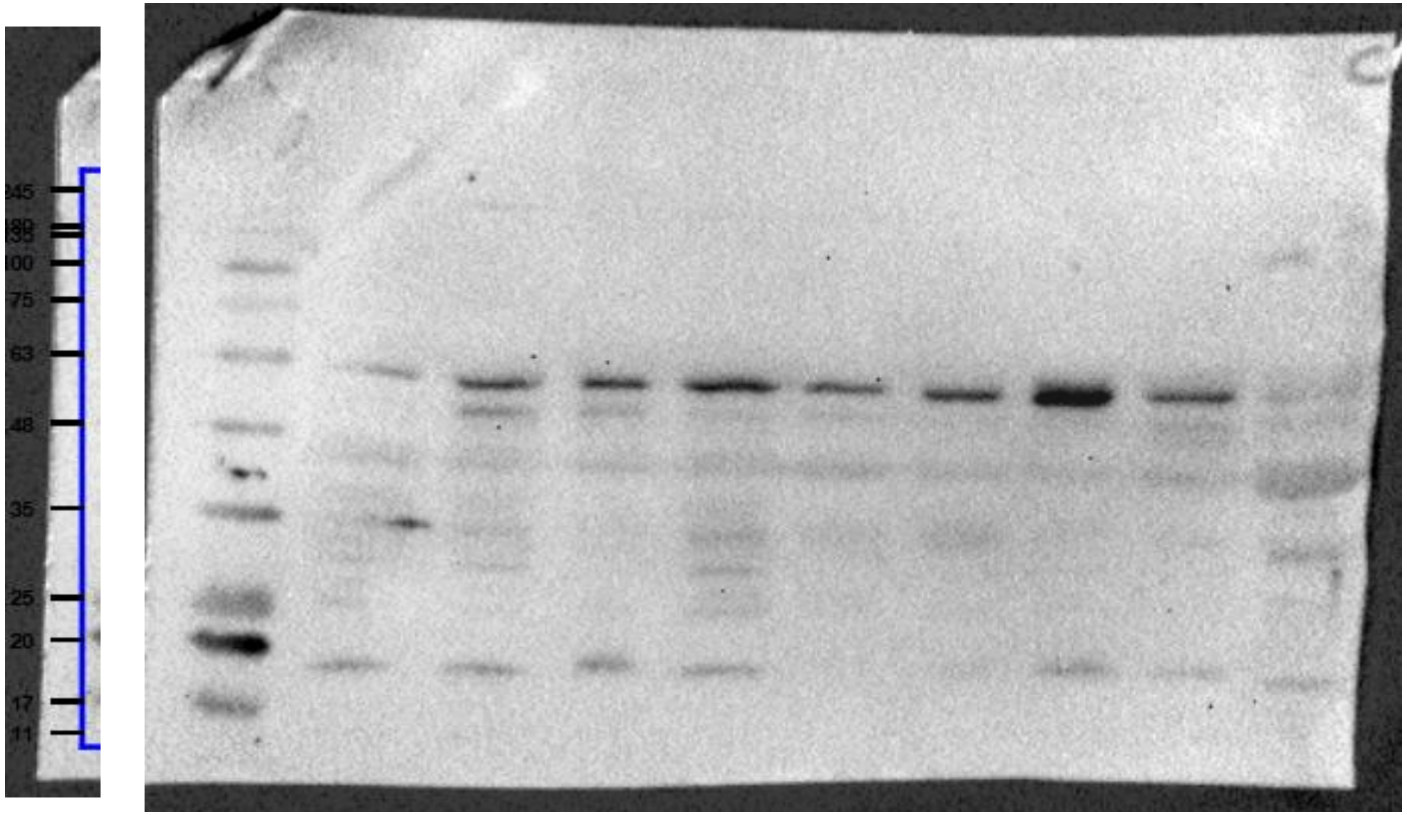

MW C DC EPI HT MA AA EPI+HT EPI+MA EPI+AA

NFkB-Phase D

Biorad Image lab, (Software 6.1 Windows)

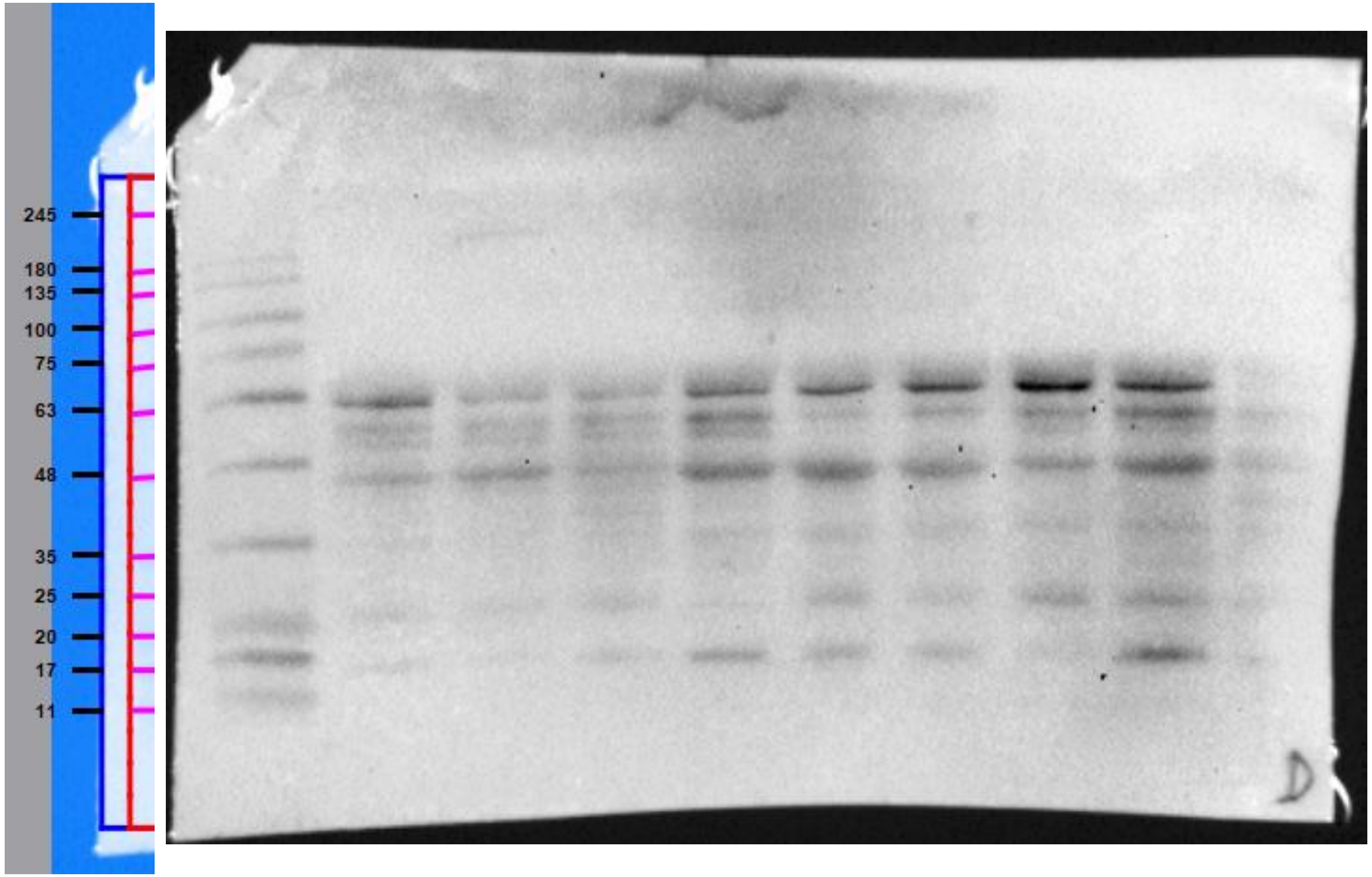

MW C DC EPI HT MA AA EPI+HT EPI+MA EPI+AA

ACTIN (NFkB)-Phase D

Biorad Image lab, (Software 6.1 Windows)

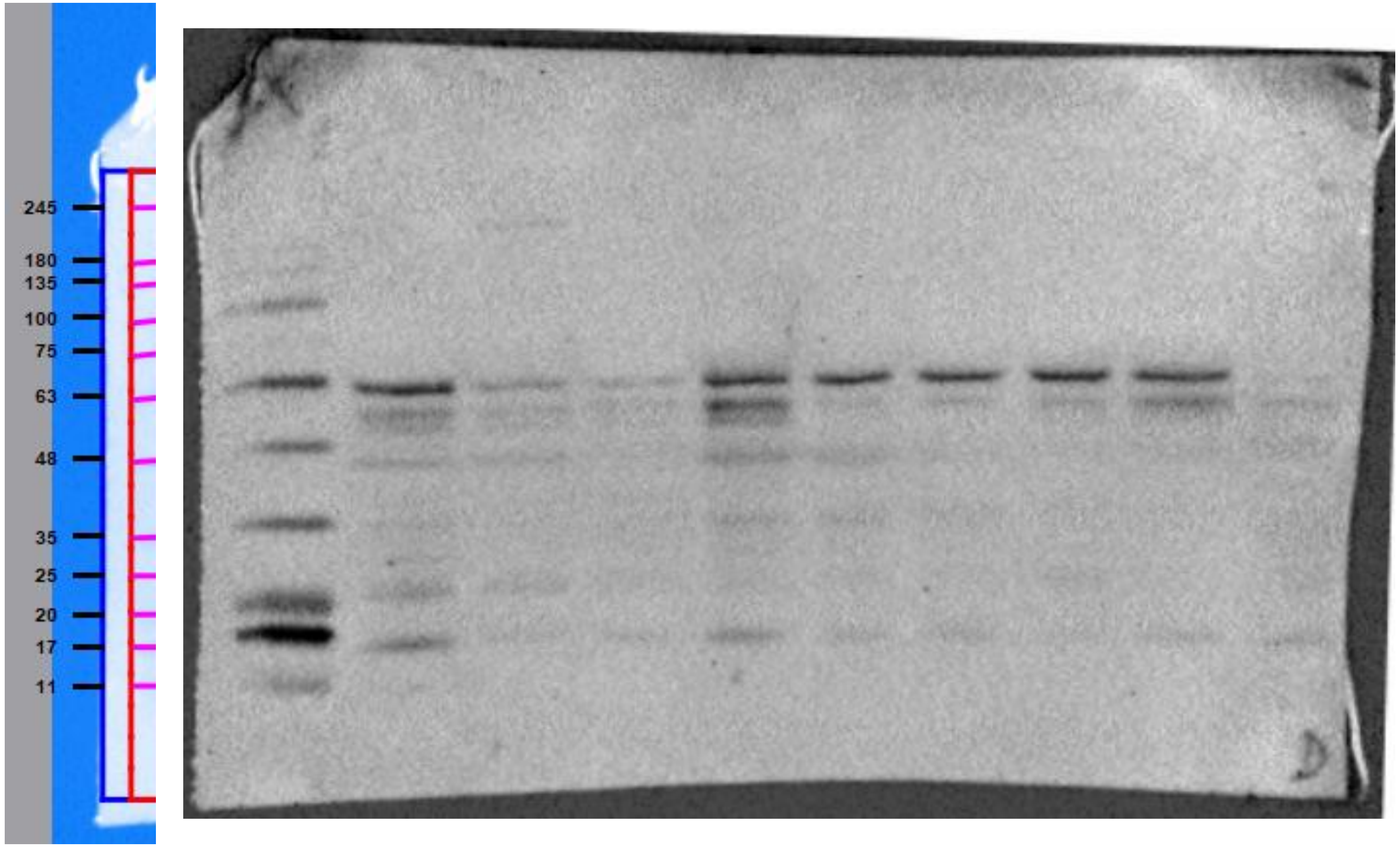

MW C DC EPI HT MA AA EPI+HT EPI+MA EPI+AA

PPAR-Phase A

Biorad Image lab, (Software 6.1 Windows)

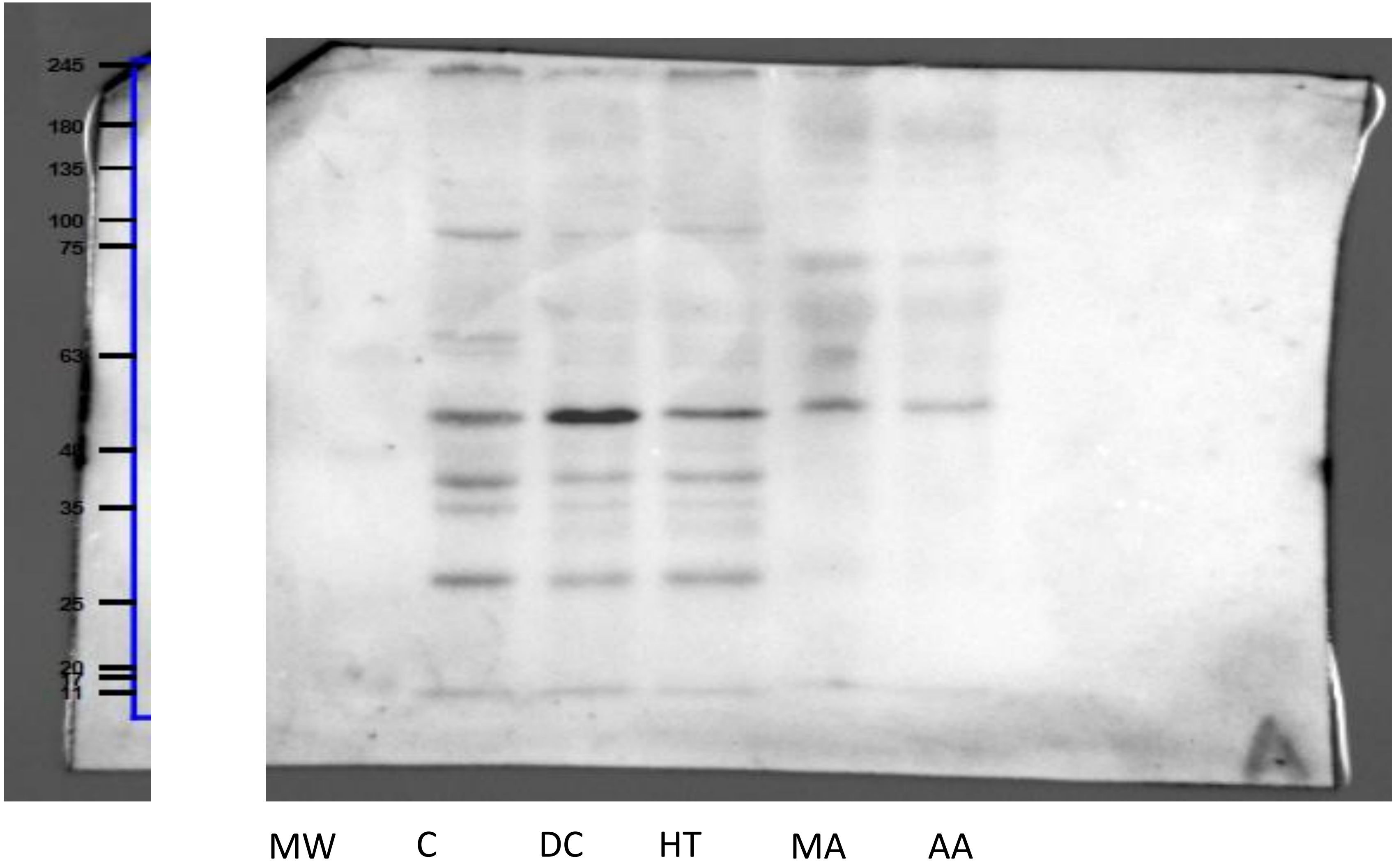

ACTIN (PPAR)-Phase A

Biorad Image lab, (Software 6.1 Windows)

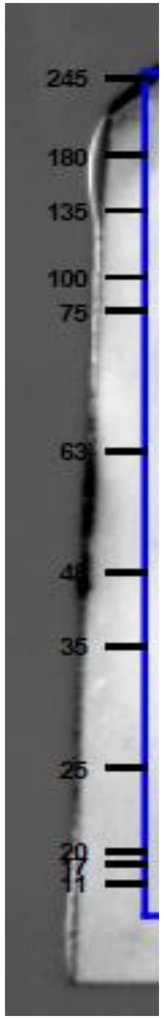

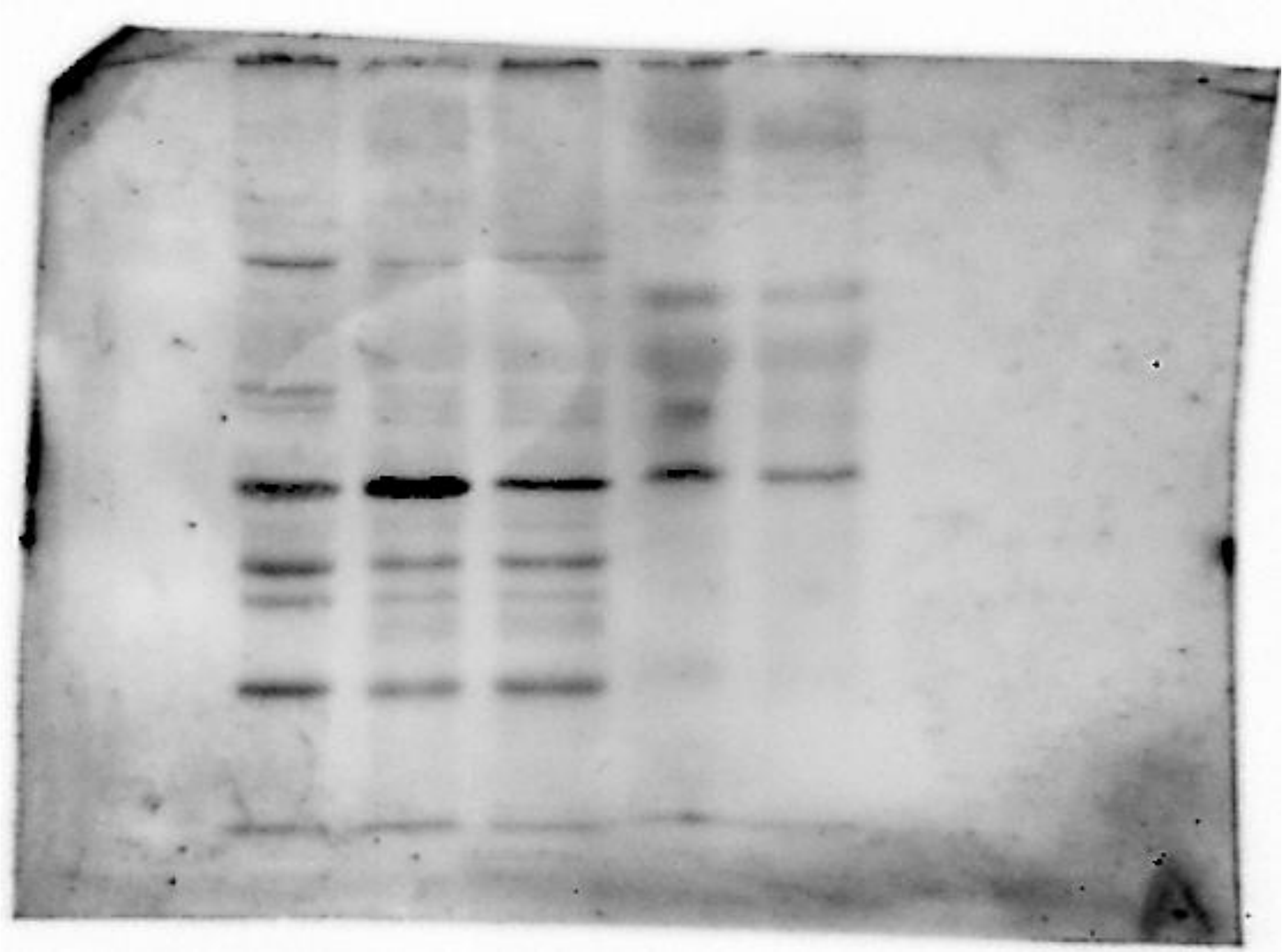

MW C DC HT MA AA

PPAR-Phase B

Biorad Image lab, (Software 6.1 Windows)

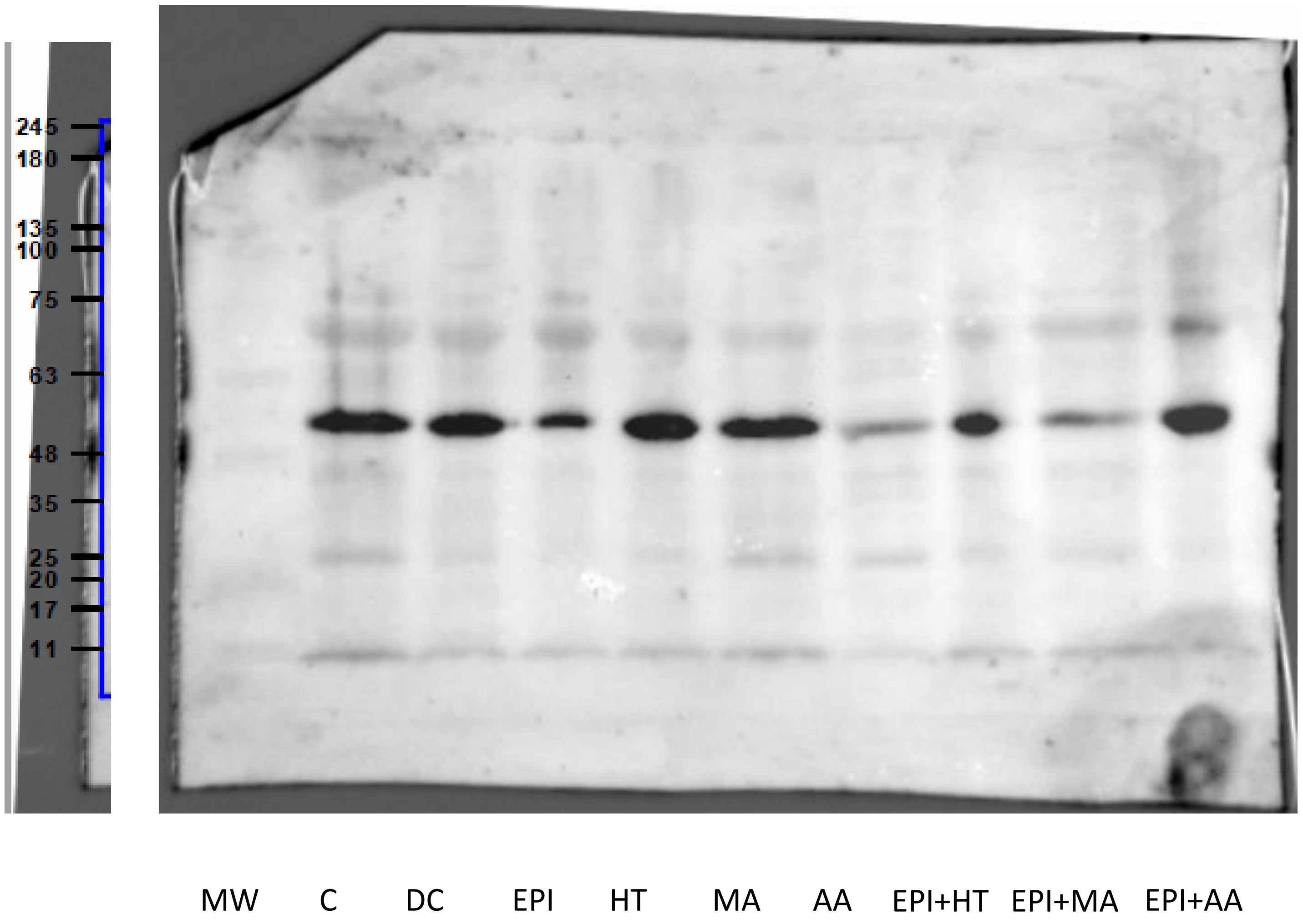

ACTIN (PPAR)-Phase B

Biorad Image lab, (Software 6.1 Windows)

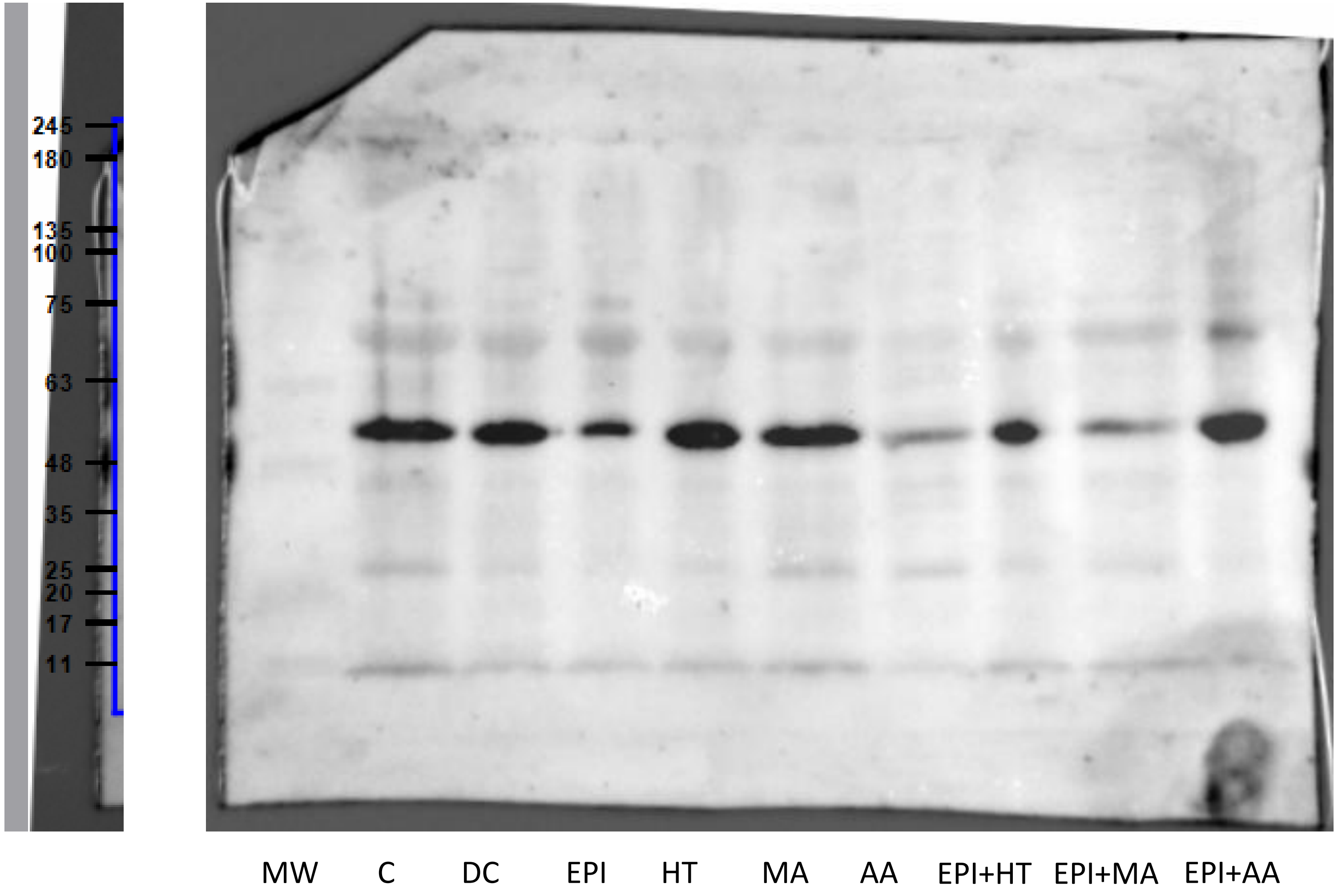

PPAR-Phase C

Biorad Image lab, (Software 6.1 Windows)

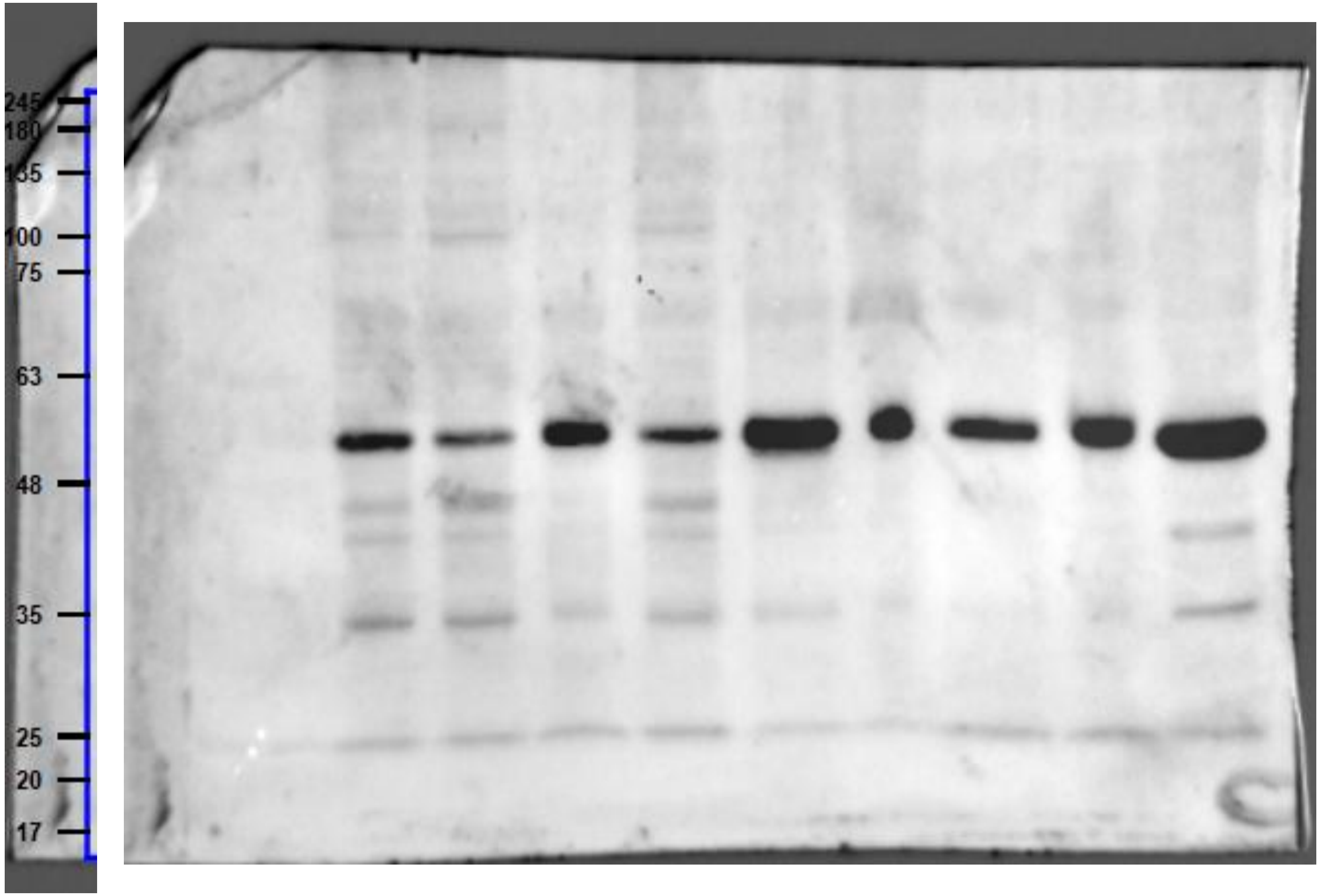

MW C DC EPI HT MA AA EPI+HT EPI+MA EPI+AA

ACTIN (PPAR)-Phase C

Biorad Image lab, (Software 6.1 Windows)

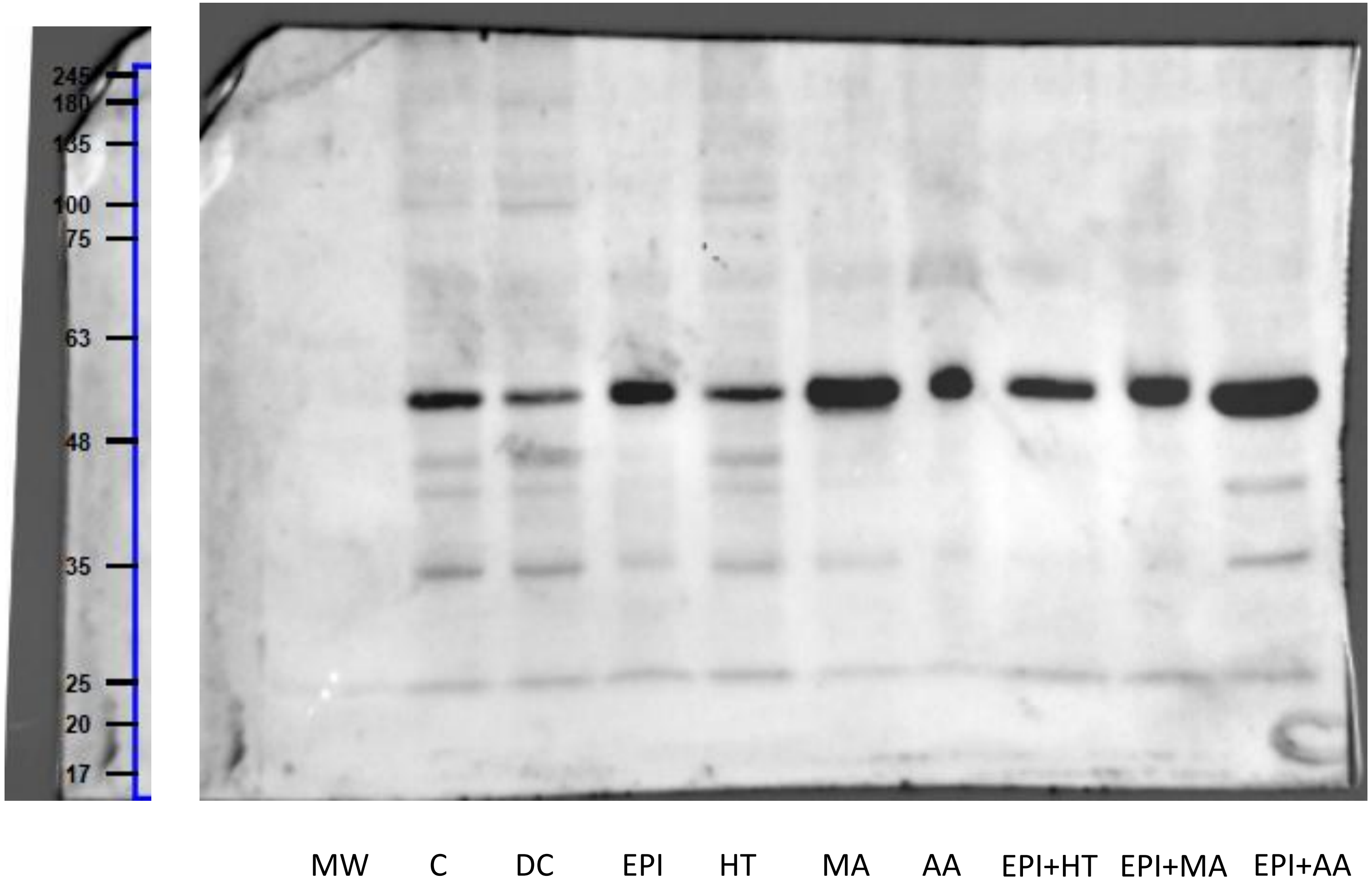

PPAR-Phase D

Biorad Image lab, (Software 6.1 Windows)

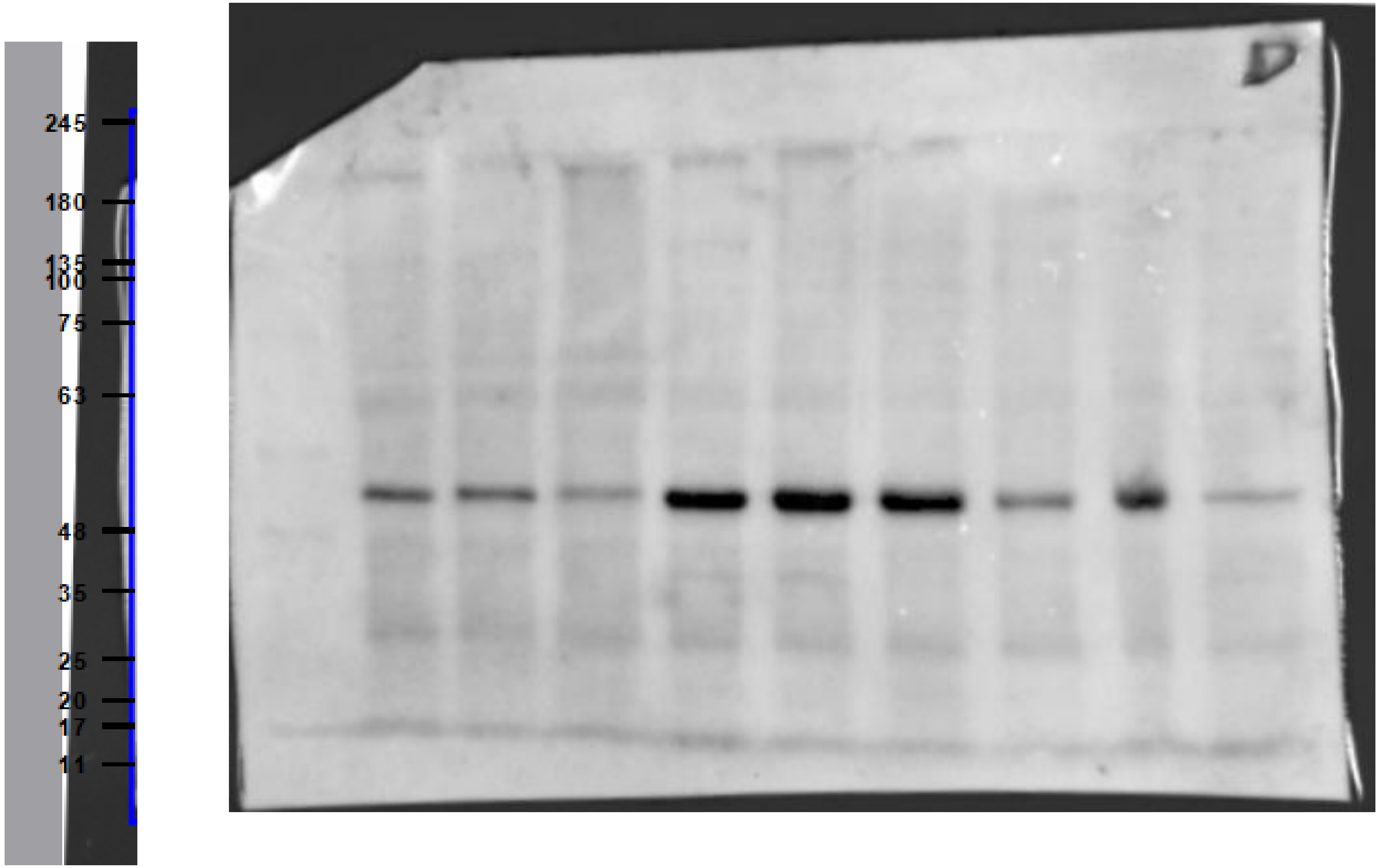

MW C DC EPI HT MA AA EPI+HT EPI+MA EPI+AA

ACTIN (PPAR)-Phase D

Biorad Image lab, (Software 6.1 Windows)

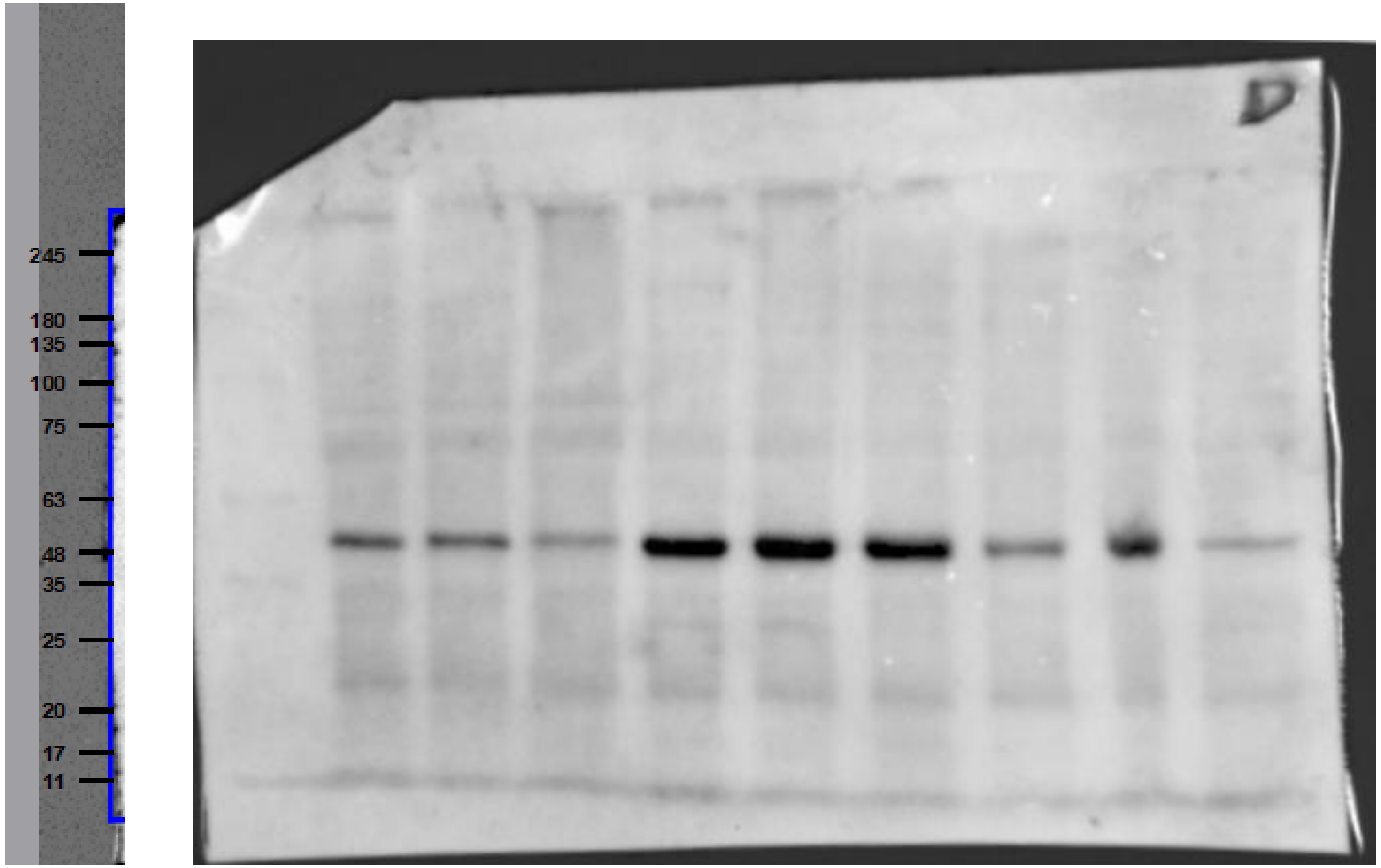

MW C DC EPI HT MA AA EPI+HT EPI+MA EPI+AA

p53-Phase A

Biorad Image lab, (Software 6.1 Windows)

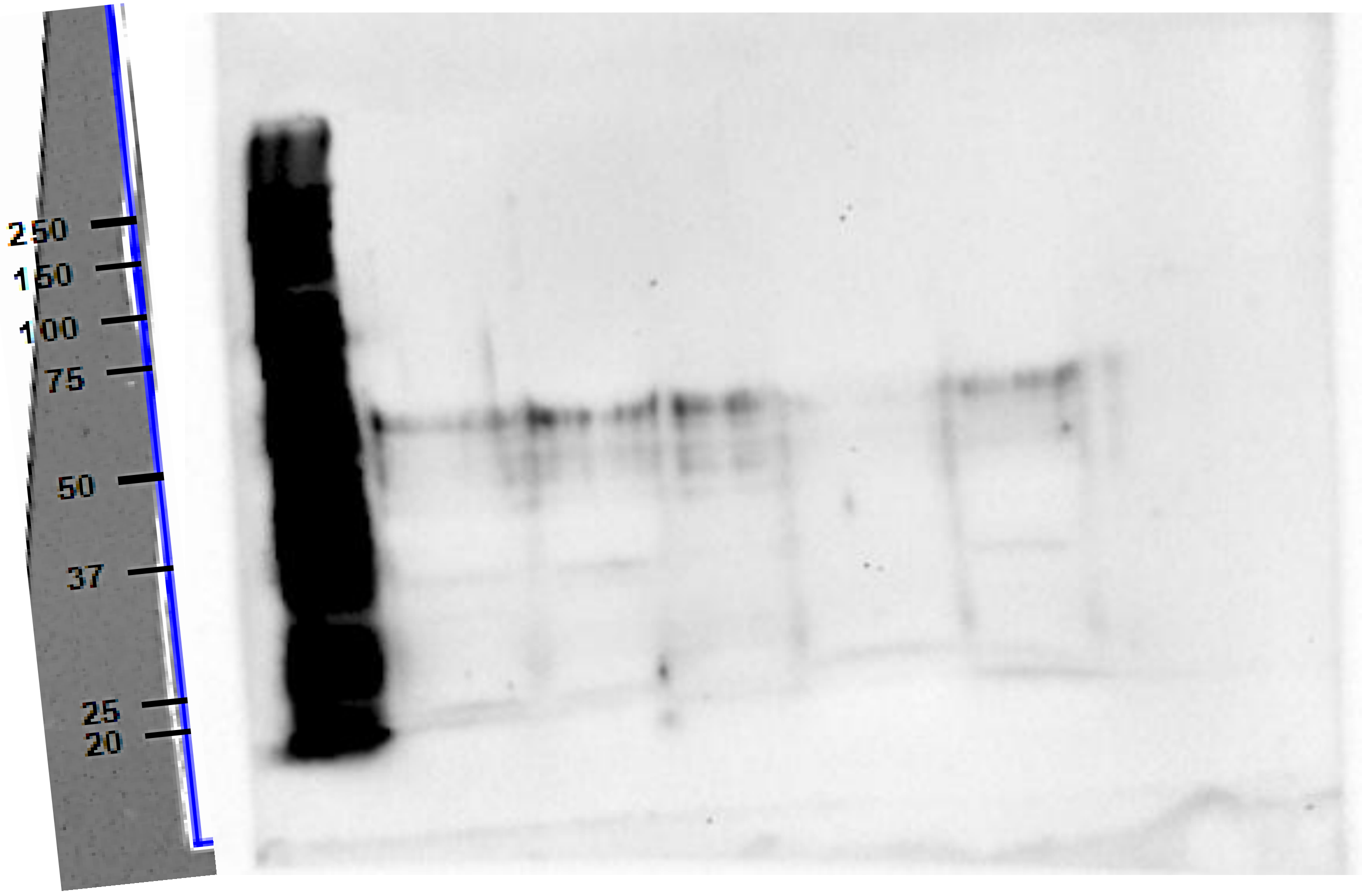

MW C DC HT MA AA

ACTIN (p53)-Phase A

Biorad Image lab, (Software 6.1 Windows)

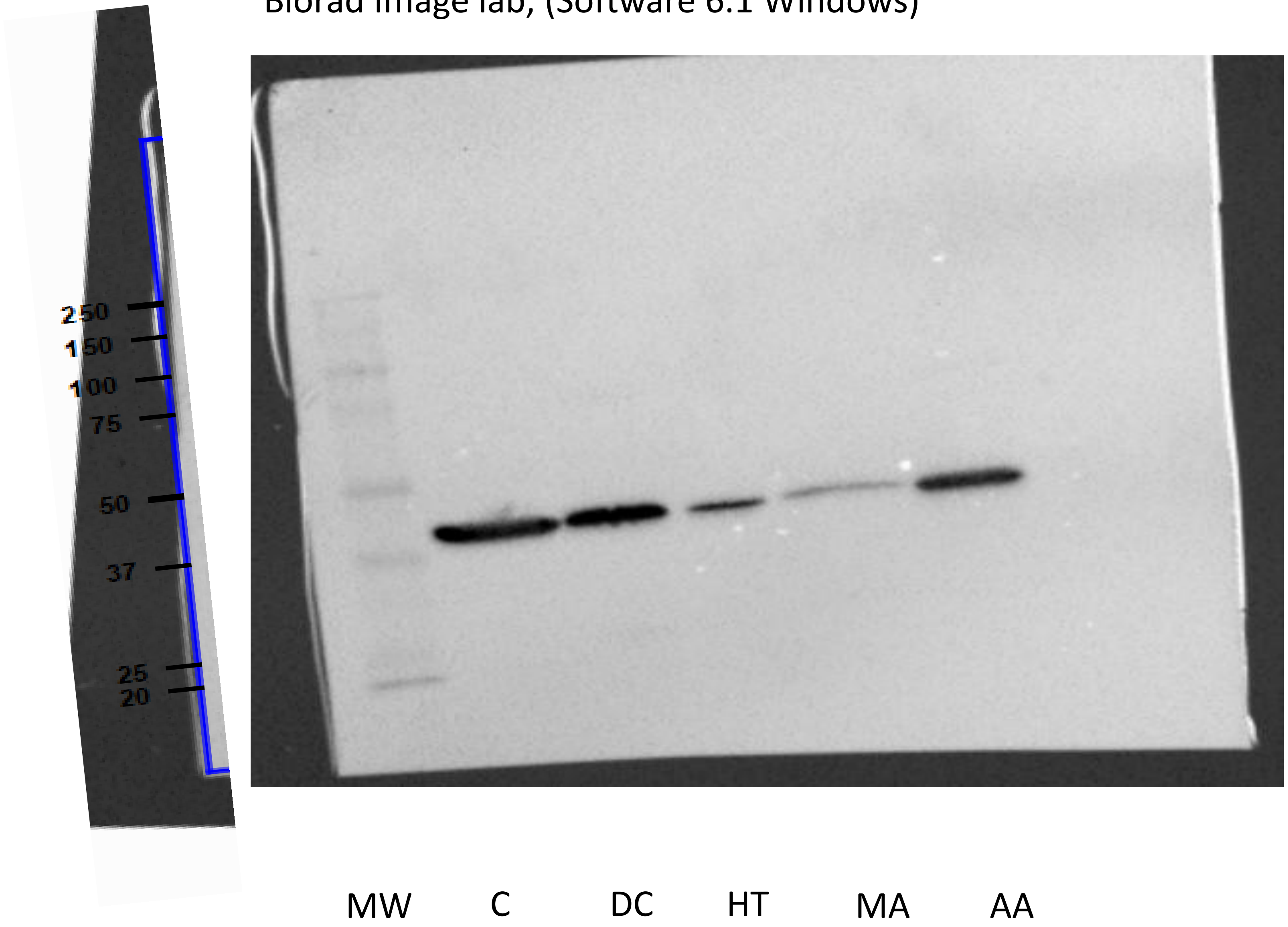

p53-Phase B

Biorad Image lab, (Software 6.1 Windows)

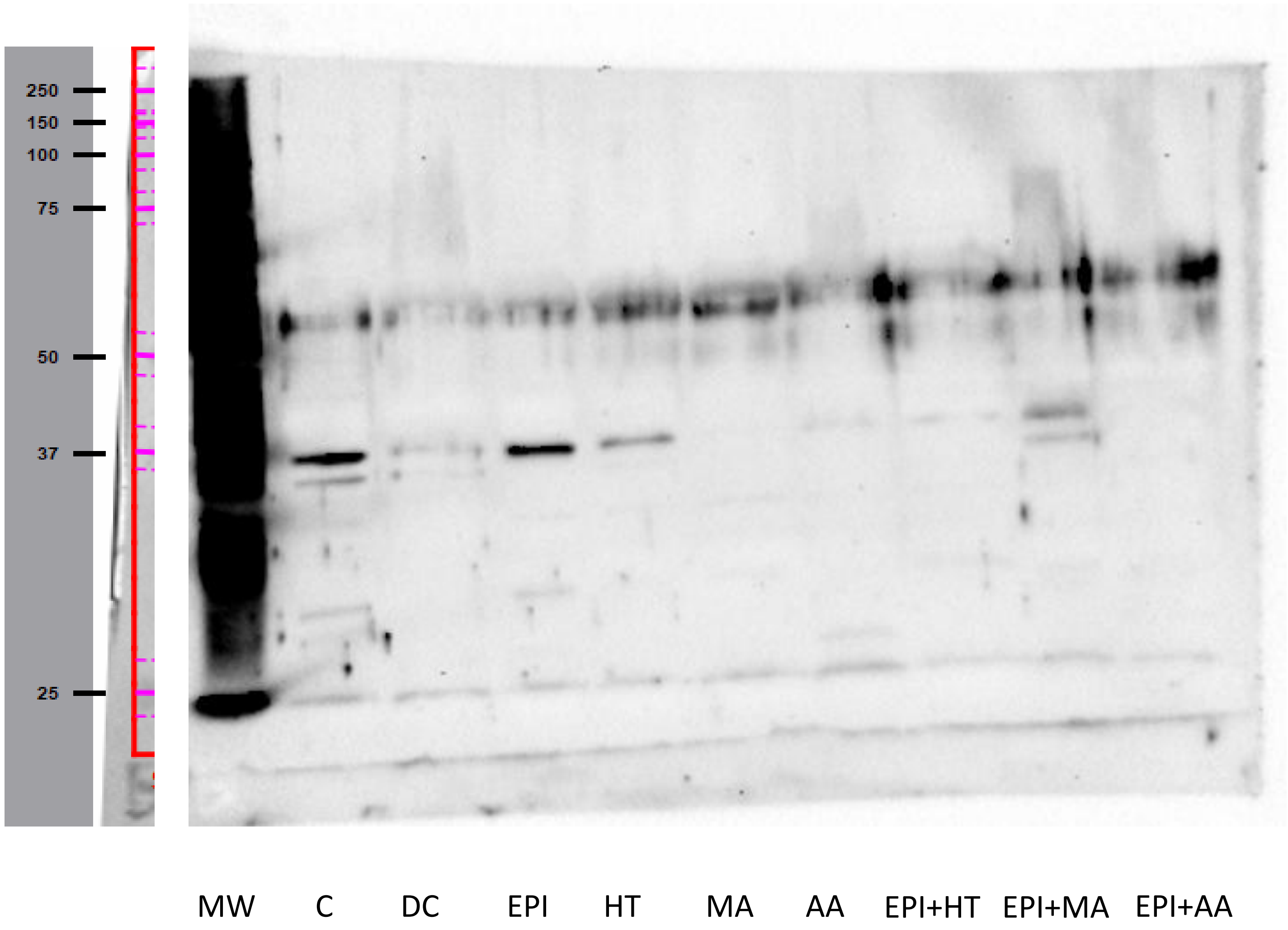

ACTIN (p53)-Phase B

Biorad Image lab, (Software 6.1 Windows)

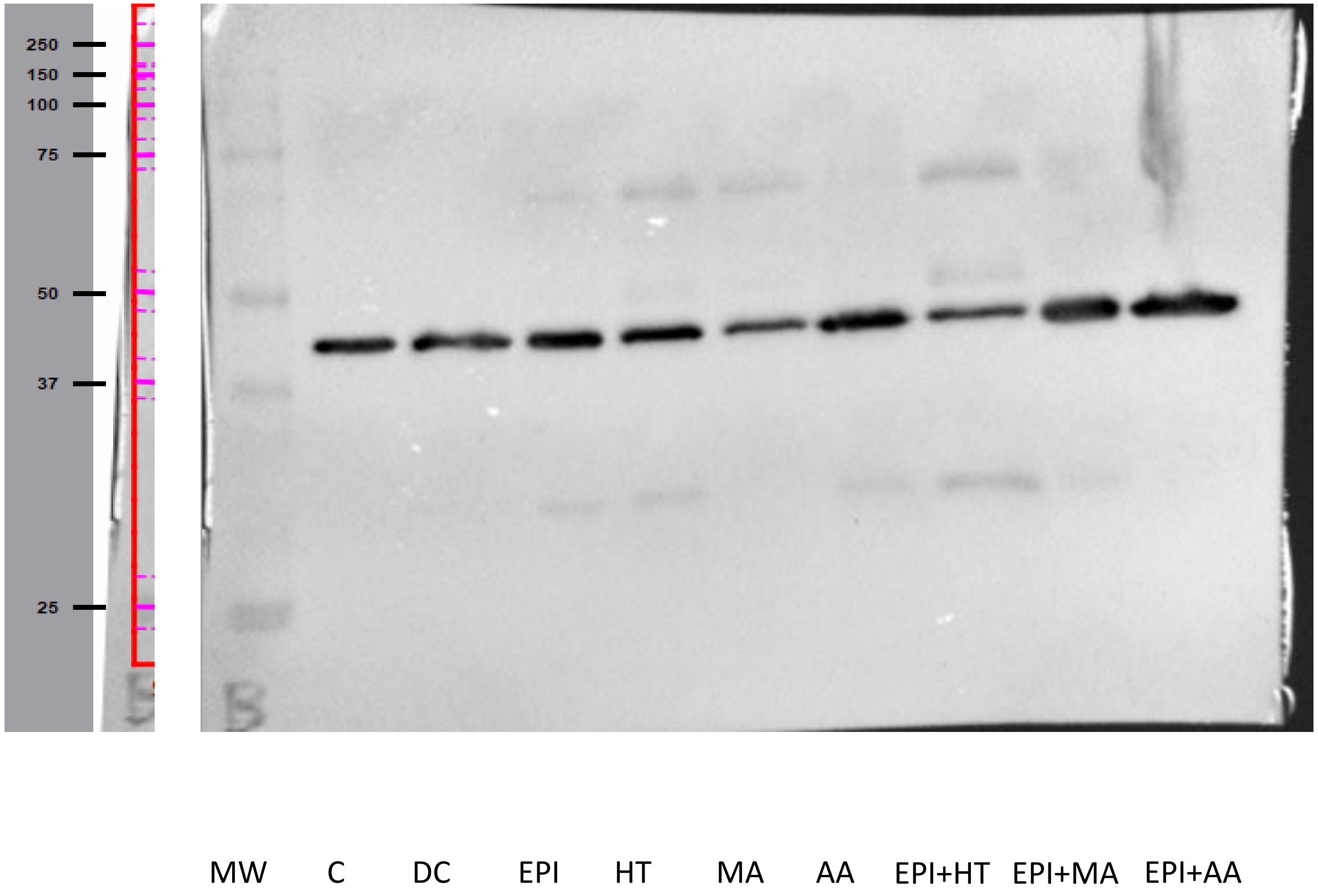

p53-Phase C

Biorad Image lab, (Software 6.1 Windows)

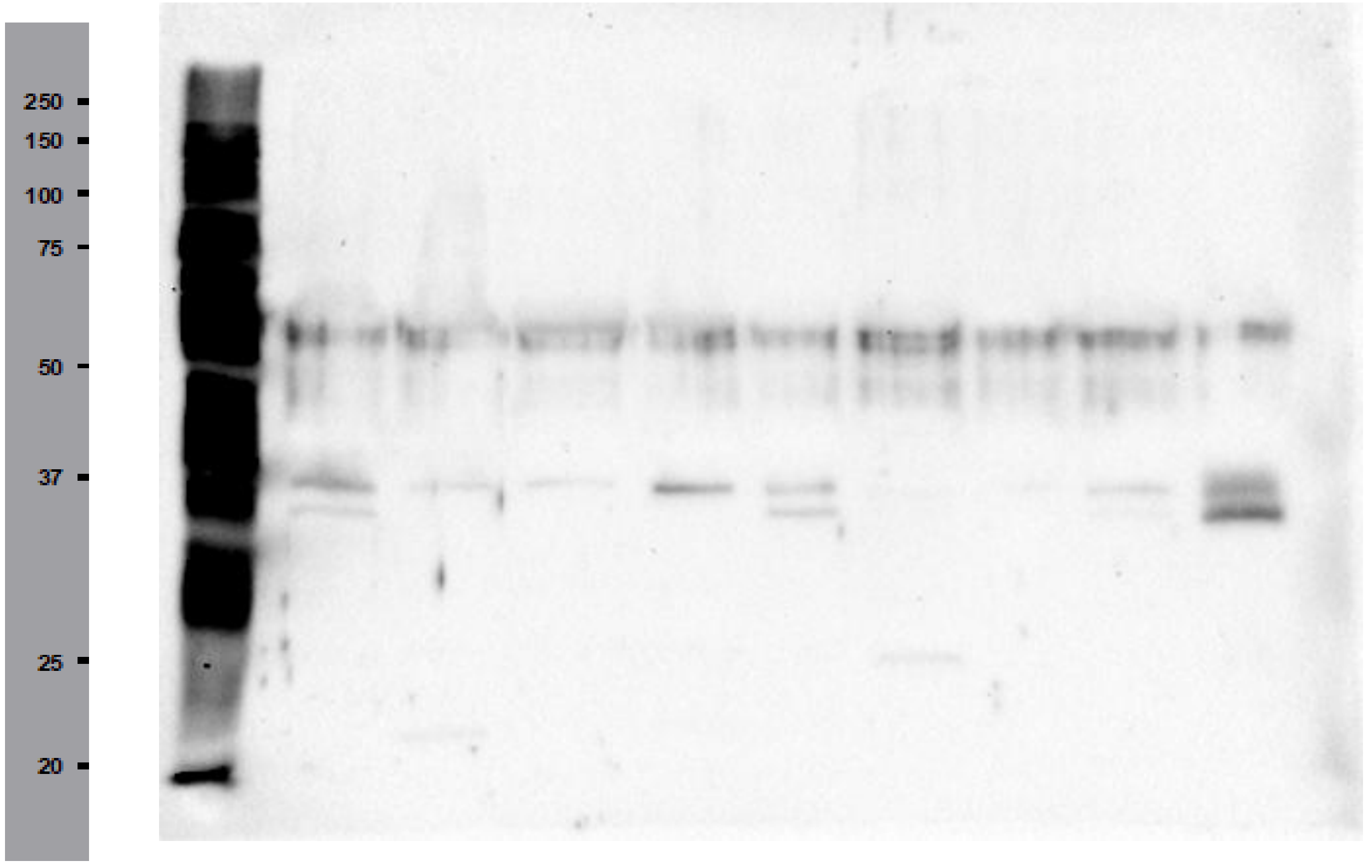

ACTIN (p53)-Phase C

Biorad Image lab, (Software 6.1 Windows)

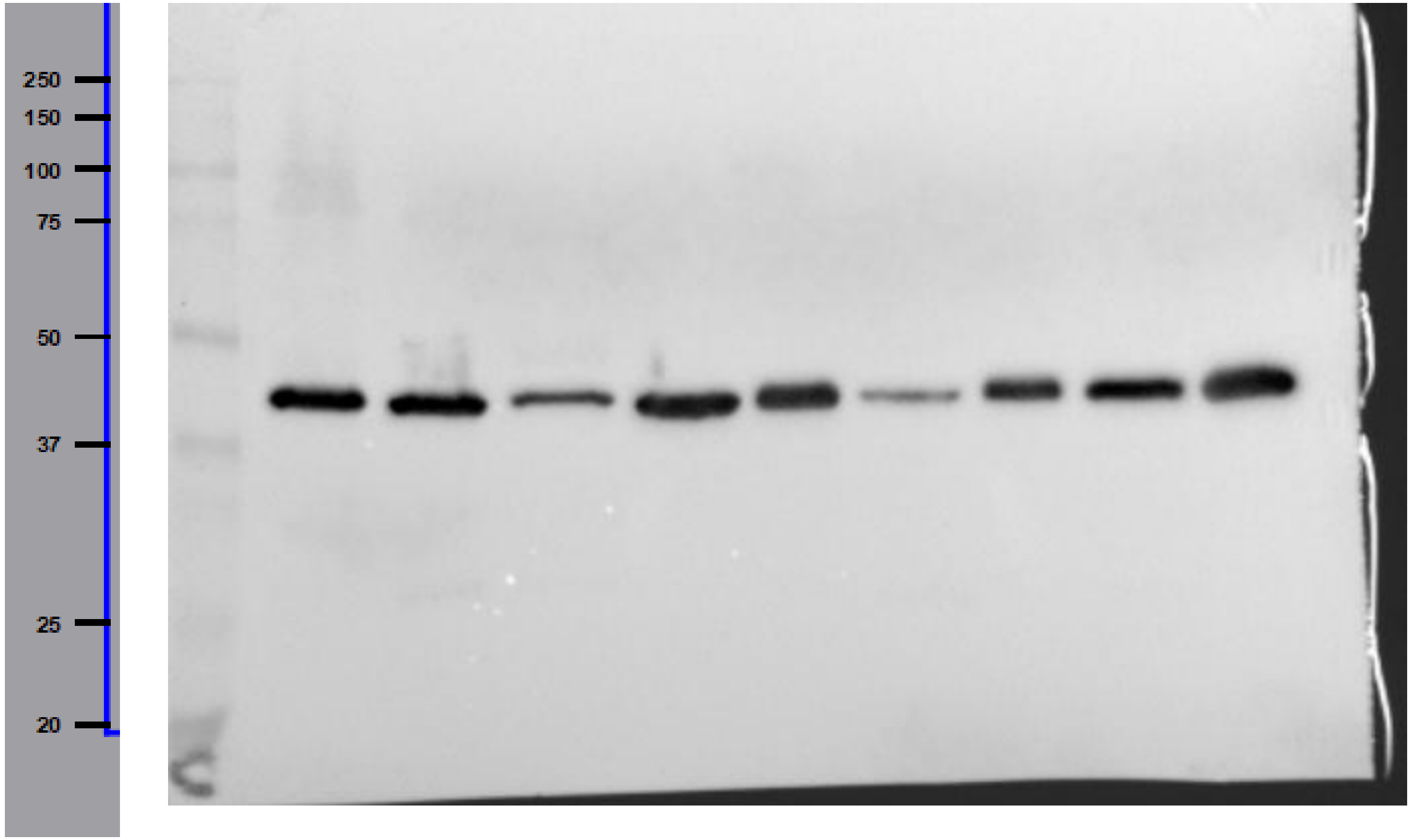

MW C DC EPI HT MA AA EPI+HT EPI+MA EPI+AA

p53-Phase D

Biorad Image lab, (Software 6.1 Windows)

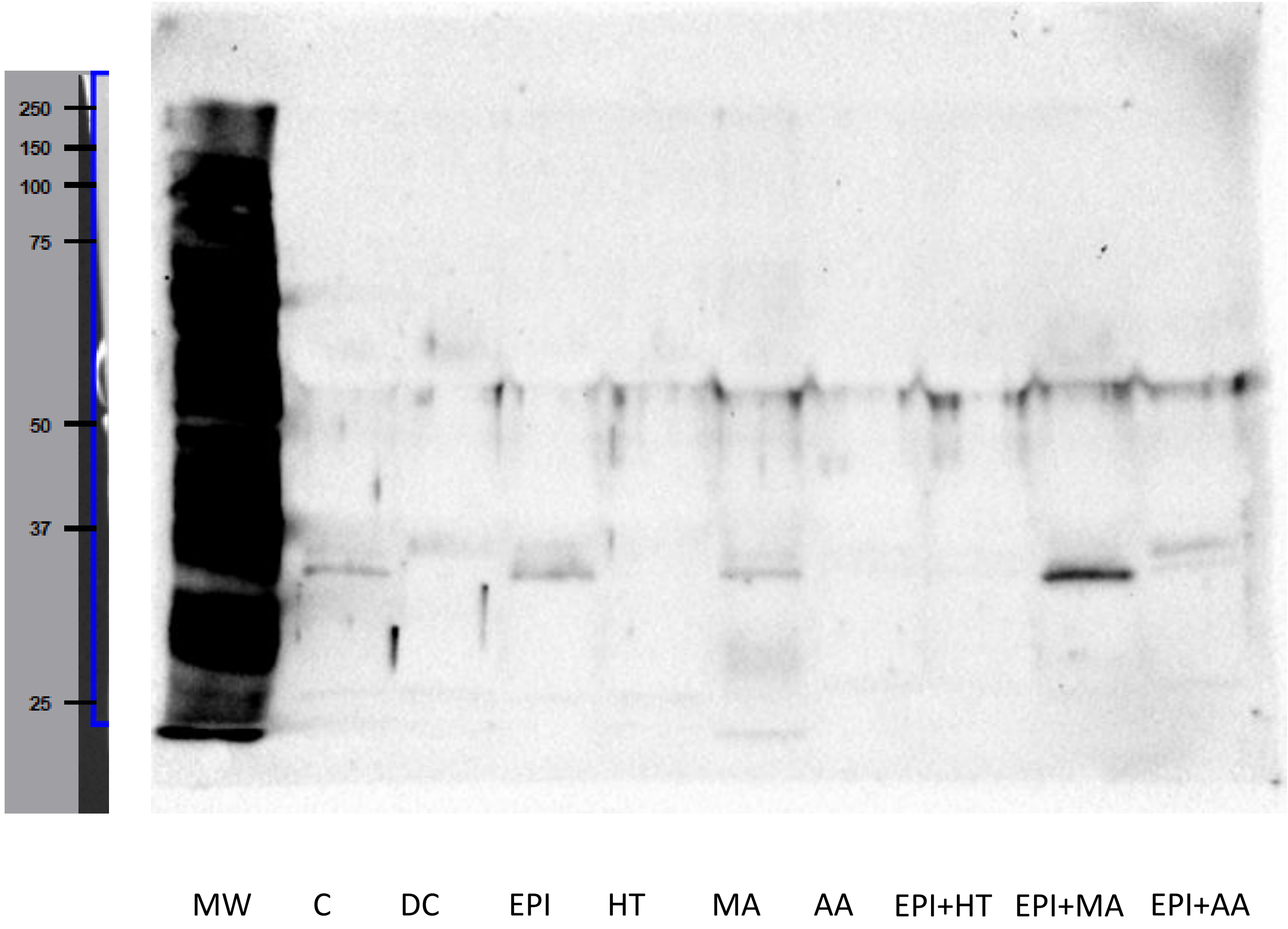

ACTIN (p53)-Phase D

Biorad Image lab, (Software 6.1 Windows)

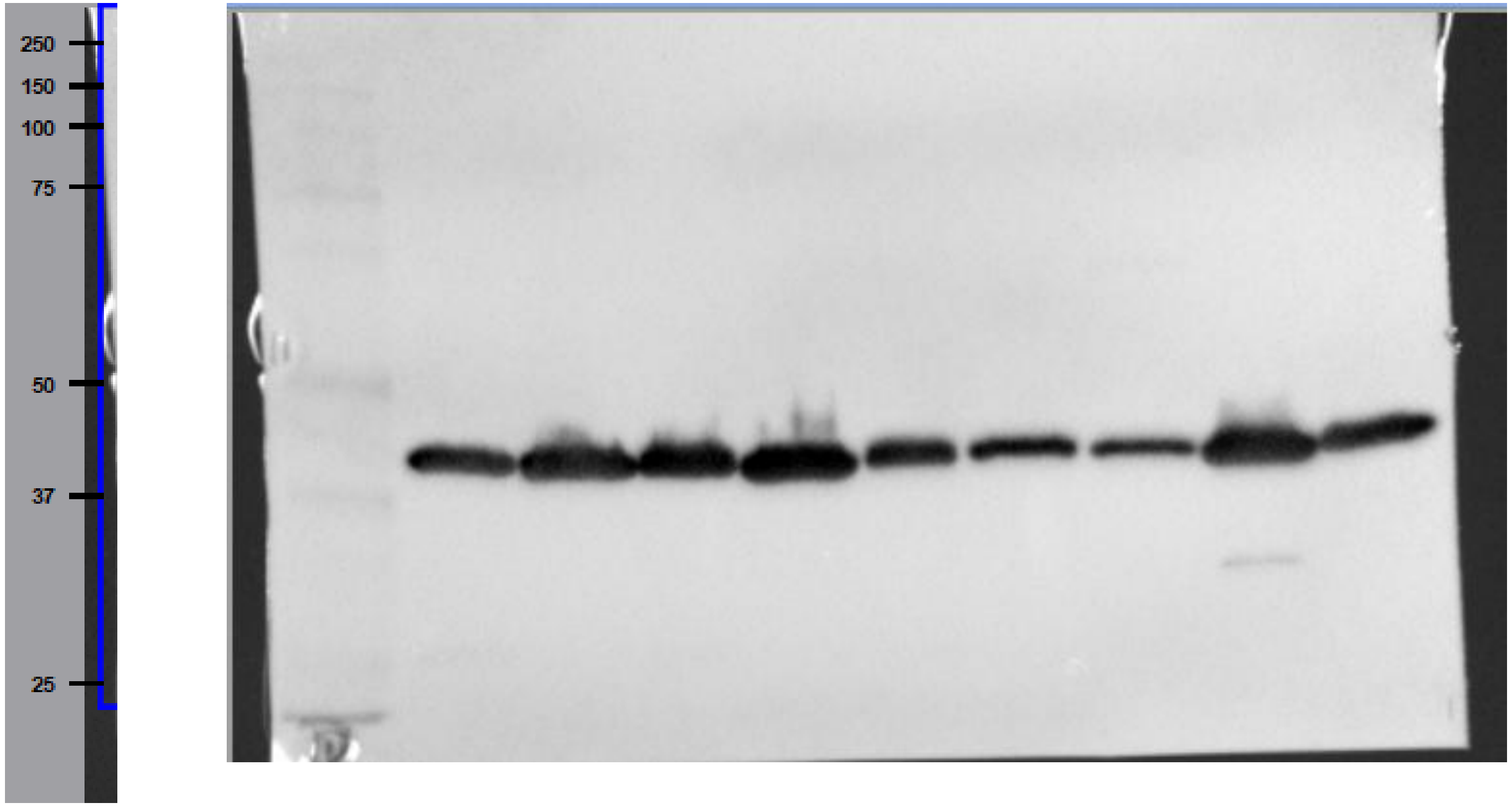

MW C DC EPI HT MA AA EPI+HT EPI+MA EPI+AA
